# Outcomes of the 340B Drug Pricing Program: A Scoping Review

**DOI:** 10.1001/jamahealthforum.2023.3716

**Published:** 2023-11-22

**Authors:** Ryan P. Knox, Junyi Wang, William B. Feldman, Aaron S. Kesselheim, Ameet Sarpatwari

**Affiliations:** 1 Harvard-MIT Center for Regulatory Science, Harvard Medical School, Boston, Massachusetts; 2 Program On Regulation, Therapeutics, And Law (PORTAL), Division of Pharmacoepidemiology and Pharmacoeconomics, Department of Medicine, Brigham and Women’s Hospital and Harvard Medical School, Boston, Massachusetts; 3 Division of Pulmonary and Critical Care Medicine, Department of Medicine, Brigham and Women’s Hospital and Harvard Medical School, Boston, Massachusetts

## Abstract

**Importance:**

The 340B Drug Pricing Program requires manufacturers to offer discounted drug prices to support safety net hospitals and clinics (covered entities) providing care to low-income populations. Amid expansion, the program has received criticism and calls for reform.

**Objective:**

To assess the literature on the foundations of and outcomes associated with the 340B program.

**Evidence Review:**

The databases searched in this scoping review included PubMed, Embase, EconLit, National Bureau of Economic Research (NBER), Westlaw, the Department of Health and Human Services Office of the Inspector General (HHS-OIG) website, the Government Accountability Office (GAO) website, and Google in February 2023 for peer-reviewed literature, legal publications, opinion pieces, and government agency and committee reports related to the 340B program.

**Findings:**

Among a collected 900 documents, 289 met inclusion criteria: 83 articles from PubMed, 12 articles from Embase, 2 articles from EconLit, 1 article from NBER, 28 articles from Westlaw, 23 legislative history documents, 103 documents from Google, 11 GAO reports, and 26 HHS-OIG reports. Included literature pertained to 4 stakeholders in the 340B program: covered entities, pharmacies, pharmaceutical manufacturers, and patients. This literature showed that hospitals, clinics, and pharmacies generated revenue and manufacturers have forgone revenue from 340B discounted drugs. Audits of covered entities found low rates of compliance with 340B program requirements, whereas mixed evidence was uncovered on how covered entities used their 340B revenue, with some studies suggesting use to expand health care services for low-income populations and others to acquire physician practices and open sites in higher-income neighborhoods. These studies were hampered by a lack of transparency and reporting on the use of 340B revenue. Studies revealed patient benefits from access to expanded health care services, but there was mixed evidence on patient cost savings. Although the review identified considerable research on 340B hospitals, pharmacies, and patients, less research was found evaluating the 340B program’s effect on nonhospital covered entities, drug pricing, and racial and ethnic minority groups.

**Conclusions and Relevance:**

In this scoping review of the 340B program, we found that the 340B program was associated with financial benefits for hospitals, clinics, and pharmacies; improved access to health care services for patients; and substantial costs to manufacturers. Increased transparency regarding the use of 340B program revenue and strengthened rulemaking and enforcement authority for the Health Resources and Services Administration would support compliance and help ensure the 340B program achieves its intended purposes.

## Key Points

**Question:**

How has the 340B Drug Pricing Program affected the US health care system?

**Findings:**

This scoping review found evidence that the 340B program was associated with revenue to hospitals, clinics, and pharmacies; expanded services for patients; and costs to pharmaceutical manufacturers. The study found mixed evidence that 340B revenue funded health care specifically for low-income populations.

**Meaning:**

The 340B program has benefited hospitals, clinics, pharmacies, and patients, but its expansion has led to calls for reform.

## Teaser

This scoping review assesses the literature on the foundations of and outcomes associated with the 340B Drug Pricing Program in the US health care system.

## Introduction

The 340B Drug Pricing Program was created in 1992 to support safety net hospitals and clinics caring for low-income and underserved populations by discounting the cost of outpatient drugs.^1^ The revenue from dispensing these discounted drugs allows these entities to reach more patients, provide more health care services and programs, and subsidize uncompensated care.

The origins of the 340B program stem from the establishment of the Medicaid Drug Rebate Program in 1990, which requires manufacturers to pay statutory rebates on drugs purchased by state Medicaid programs. These rebates include a best price discount to ensure that Medicaid pays no more than the lowest price paid by commercial insurers.^2^ Many safety net hospitals and federally funded clinics had previously received substantial discounts on drugs purchased directly from manufacturers.^3,4^ However, after the enactment of the Medicaid Drug Rebate Program, manufacturers ceased offering these discounts, reportedly because they would be included in best price calculations.^5^ The resulting higher prices strained the budgets of hospitals and clinics, which then reduced their ability to provide health care services.^3,5^

**Figure 1.**
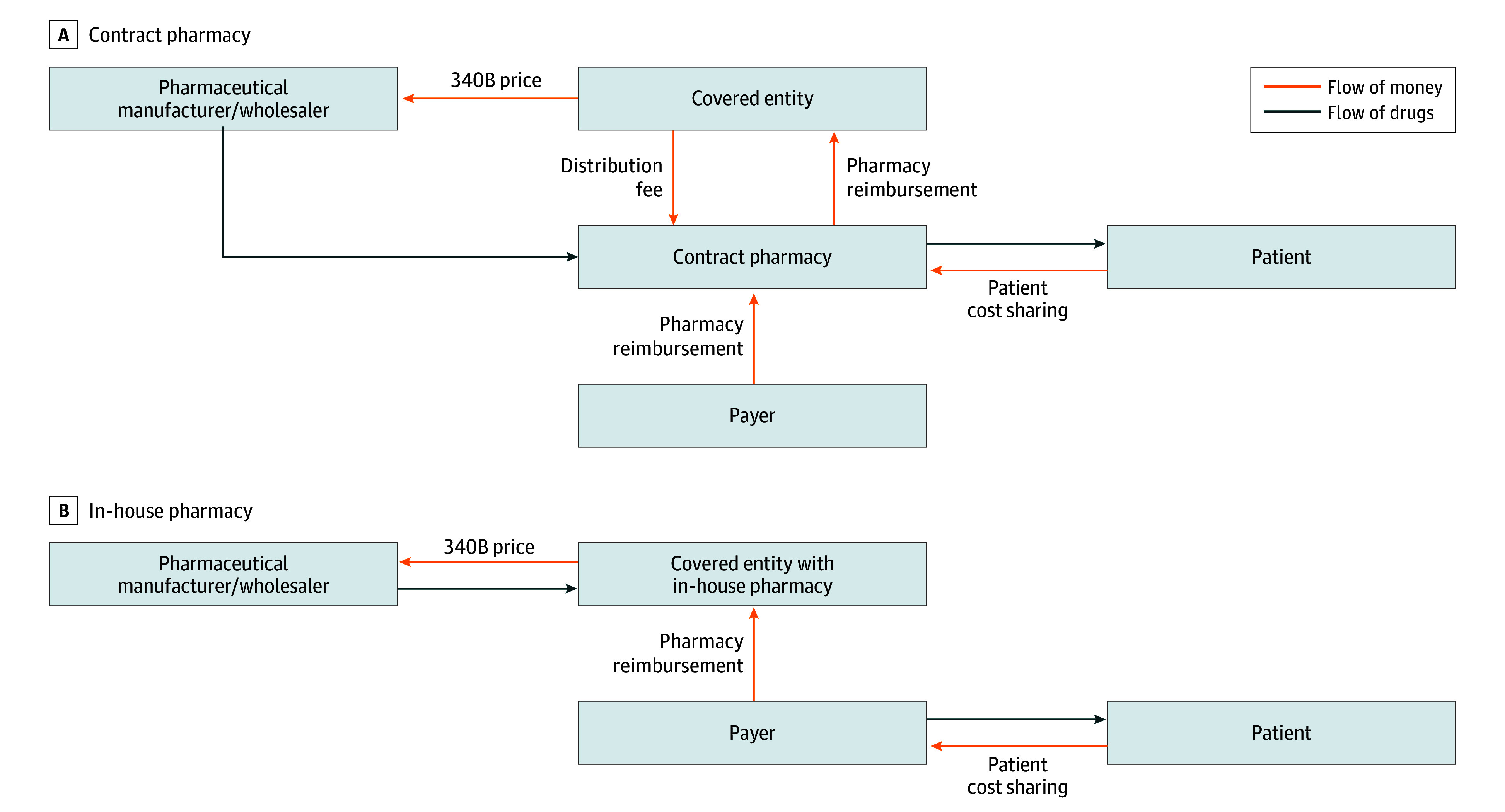
Flow of Money and Drugs in the 340B Drug Pricing Program

The 340B program, enacted in response to these events, requires manufacturers participating in Medicaid to sell drugs at discounts to eligible clinics and hospitals, called “covered entities,”^6^ and permits these entities to charge nondiscounted prices to all payers (Figure 1), generating revenue that could be used to subsidize health care services and operations.^5^ Discounts are based on the average manufacturer price of the drug, or the average price wholesalers and retail pharmacies pay manufacturers for drugs distributed at retail pharmacies.^7^ The 340B discounted price is equal to the average manufacturer price minus the average Medicaid rebate for a unit of that drug during the preceding quarter.^8^ The discount is approximately 20% to 50%,^9^ but can be higher because manufacturers of brand-name drugs subject to substantial price hikes over many years—such as adalimumab (Humira) and some insulins—are required to provide additional Medicaid rebates for price increases exceeding inflation.^10,11,12^

Authority over the 340B program was vested with the Department of Health and Human Services (HHS), which delegated authority to the Health Resources and Services Administration (HRSA) (Table). Initially, 13 categories of covered entities could participate, primarily federal grantee clinics and disproportionate share hospitals caring for many low-income patients and Medicaid patients. Congress added children’s hospitals in 2005 and critical access hospitals, free-standing cancer hospitals, rural referral centers, and sole community hospitals in 2010 (Box).

**Table.** Key Terms and Definitions

Term	Definition
340B Statute	42 USC Section 256b
Child sites	Off-site outpatient clinics associated with covered entities
CMS	Centers for Medicare and Medicaid Services, the agency within the US Department of Health and Human Services responsible for overseeing Medicare and Medicaid programs
Contract pharmacies	Retail pharmacies that contract with covered entities to dispense drugs to patients
Covered entities	Hospitals and clinics eligible to participate in the 340B Drug Pricing Program
Diversion	Dispensing a drug purchased at a 340B discount to an individual who is not a patient of a covered entity; prohibited by the 340B statute
Duplicate discounting	When a manufacturer both (1) sells a drug to a covered entity at a 340B discount and (2) pays a Medicaid rebate to the state Medicaid program on that same drug; prohibited by the 340B statute
Federal grantees	Safety net clinics eligible to participate in the 340B Drug Pricing Program based on receiving certain federal grants
GAO	Government Accountability Office, an agency that provides auditing and research services to Congress
HHS	US Department of Health and Human Services
HRSA	US Health Resources and Services Administration, the agency within the US Department of Health and Human Services responsible for overseeing the 340B Drug Pricing Program
In-house pharmacies	Pharmacies owned by covered entities

Box. **Categories of Covered Entities**

**Hospital Covered Entities**

Disproportionate share hospitals
Children’s hospitals
Critical access hospitals
Freestanding cancer hospitals
Sole community hospitals

**Federal Grantee Covered Entities**

Federally qualified health centers and look-alikes
Health centers for residents of public housing
Family planning clinics
Clinics receiving grants for outpatient early HIV/AIDS intervention services
State AIDS drug purchasing assistance programs
Black lung clinics
Comprehensive hemophilia diagnostic treatment centers
Native Hawaiian health centers
Urban Indian organizations
Ryan White program grantees
Clinics receiving funds to treat sexually transmitted diseases
Clinics receiving funds to treat tuberculosis
Rural referral centers

The 340B program places 2 key requirements on covered entities’ participation. First, it prohibits covered entities from duplicate discounting, or purchasing a drug at a 340B discount and submitting a claim to Medicaid for reimbursement that results in a rebate paid to the state Medicaid agency. Second, the 340B program bars covered entities from reselling 340B discounted drugs or providing them to patients not receiving care from the covered entity, a practice called “diversion.” Covered entities are subject to audits to ensure compliance with these provisions.

Critics of the 340B program, led by the pharmaceutical industry, have expressed concern about the program’s growth in recent years.^13^ We conducted a scoping review to assess the foundations of and outcomes associated with the 340B program.

## Methods

Our study followed the scoping review methodology set forth by Arskey and O’Malley^14^ and the Preferred Reporting Items for Systematic reviews and Meta-Analyses extension for Scoping Reviews (PRISMA-ScR) checklist.^15^

**Figure 2.**
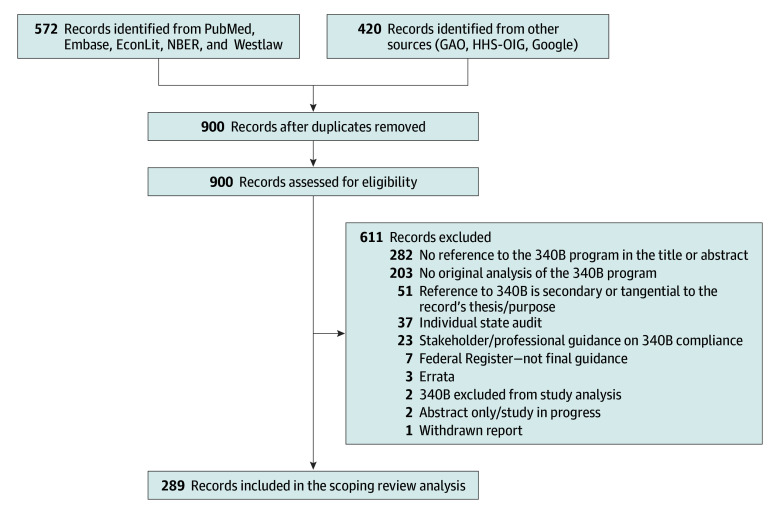
Study Selection Flow Diagram GAO indicates Government Accountability Office; HHS-OIG, US Department of Health and Human Services Office of the Inspector General.

We conducted article searches of PubMed, Embase, EconLit, NBER, and Westlaw as well as supplementary searches of Google, the Government Accountability Office (GAO) website, and the US Department of Health and Human Services Office of the Inspector General (HHS-OIG) website. Searches were updated iteratively from May 2022 to February 2023. Search terms included variations of *340B*, *340B Drug Pricing Program*, *340B Drug Discount Program*, and *340B program* (eAppendix 1 in Supplement 1). Duplicates of retrieved articles were removed. The titles and abstracts of articles were independently reviewed for inclusion by 2 authors (R.P.K. and J.W. for all sources except Westlaw and R.K. and A.S. for Westlaw), applying the exclusion criteria shown in Figure 2. Discordant categorizations for inclusion were resolved by discussion and involved a full-text review of the article. For all included articles, we recorded the (1) author, publication year, and publication type; (2) study objective or article thesis; (3) stakeholders discussed; (4) results or analyses; (5) conclusions; and (6) limitations. A wide range of document types were included in addition to articles from peer-reviewed literature, including law review articles, white papers published by various research groups, reports published by government agencies (eg, HHS-OIG, GAO, and the Congressional Research Service), Congressional committee reports and hearing transcripts, opinion pieces, blog posts, and webpages. The breadth of sources included ensured the identification of important evidence not reported in the peer-reviewed literature and was particularly valuable in identifying gaps in the evidence and translating the implications of the evidence to policy reforms.^16^ Institutional board approval was not required for the study because it did not involve human participants.

## Results

Our search yielded 900 documents, of which 289 met our inclusion criteria: 83 articles from PubMed, 12 articles from Embase, 2 articles from EconLit, 1 article from NBER, 28 articles from Westlaw, 23 legislative history documents, 103 articles from Google, 11 GAO reports, and 26 HHS-OIG Reports (Figure 2). This literature covered issues facing 4 stakeholders in the 340B program (1) covered entities, (2) pharmacies, (3) pharmaceutical manufacturers, and (4) patients (eAppendix 2 in Supplement 1).

### Covered Entities

Included articles and reports revealed the dramatic increase in covered entities participating in the 340B program since its inception.^17,18^ In 1992, there were approximately 1000 covered entities (including child sites, which are associated offsite facilities of covered entities); by 2021, there were over 50 000.^19^ In 2021, approximately 60% of covered entities were hospitals (including child sites), while 40% were federal grantee clinics.^19^ The 340B program now includes more than 40% of US hospitals.^20^

The 340B program can be lucrative for hospitals. One study found that hospitals’ mean estimated 340B profits from Medicare Part B in 2016 were $2.5 million, whereas median profits were $0.8 million, equal to 0.3% of hospital operating budgets or 9.4% of uncompensated care costs.^21^ Another study estimated that covered entities’ collective profits doubled from $20.2 billion in 2015 to $40.5 billion in 2019.^22^

The locations of covered entities, particularly hospitals, have changed over time. One study found that disproportionate share hospitals joining the 340B program since 2004 served higher-income communities compared with disproportionate share hospitals joining before 2004.^23^ Another study similarly found that disproportionate share hospitals joining before 2004 were located in counties with lower income levels and higher uninsurance rates.^24^

Covered entities are not required to report how they use 340B revenue as a condition of participation, creating challenges in studying this spending. In spite of this limitation, 340B revenue appeared to fund a range of health care services and programs. However, study findings conflicted as to whether the revenue is primarily directed toward charity care and low-income populations. Surveys and self-reported data from covered entities indicated that 340B program revenue funded free or low-cost medications for patients and subsidized uncompensated care and specialty clinics for diabetes, cancer, stroke, and brain injuries.^25,26,27,28,29^ One study found that 340B participation of disproportionate share hospitals was associated with a 29% increase in charity care spending, a 4% increase in discounted care, and a 19% increase in the income eligibility limit for discounted care, but was not associated with the offering of low-profit medical services.^30^ Another study found that 340B hospitals provided more medication access services and outpatient treatment services for drugs, alcohol, and HIV/AIDS compared with non-340B hospitals.^31^

By contrast, 1 study^32^ found no evidence that hospitals increased uncompensated care after joining the 340B program. The GAO found in a study of almost 3000 hospitals that, although most 340B hospitals provided more uncompensated care and charity care than nonparticipating hospitals, 14% of the 340B disproportionate share hospitals studied were among the bottom quarter of all hospitals studied in providing uncompensated care.^20^ Overall, 340B hospitals also increasingly purchased outpatient oncology clinics, moving oncology care from community-based practices to hospital outpatient sites.^30,33,34^ This consolidation may increase cost of care because outpatient sites often provide more expensive services not offered in physician’s offices.^20,33,34^ However, similar consolidation was observed among oncology practices and non-340B hospitals,^35^ making unclear the association with the 340B program.

Fewer included articles and reports focused on nonhospital 340B covered entities. In a survey of 31 hemophilia treatment centers, all reported that salaries of staff (including nurses, social workers, and physical therapists) were supported by 340B revenue and almost half used 340B revenue to provide patients with financial assistance for transportation to access care.^36^ One study^37^ concluded that the 340B program saved sexually transmitted disease clinics almost 100% on the cost of penicillin treatment for syphilis, whereas another found that 55% of rural hospitals used 340B revenue to be able to stay open.^38^ Similarly, a study^39^ found hepatitis C virus infection treatment programs would lose $370 per patient and not be financially sustainable without revenue from the 340B program.

Covered entities’ compliance with 340B program requirements has been closely scrutinized. The HRSA audits of covered entities between 2012 and 2016 found noncompliance rates (rates of 1 or more violations of 340B program requirements) between 63% and 82%.^40^ A 2020 GAO study^41^ of 1242 HRSA audits from 2012 through September 2020 found similarly high rates of noncompliance. Examples of noncompliance included recordkeeping flaws regarding 340B program eligibility, duplicate discounting, and diversion.

### Pharmacies

Included literature revealed that covered entities have contracted with external pharmacies to dispense discounted drugs since the start of the 340B program.^42,43^ Contract pharmacies were essential to the program because most covered entities lacked in-house pharmacies^42^ and contract pharmacies made receiving prescription drugs more convenient for patients.^43^ Contract pharmacies dispense drugs purchased by covered entities at 340B discounts to patients of the covered entities. In return, the pharmacies are paid a fee per prescription filled and in some cases a percentage of the revenue from 340B prescriptions.^26^ One study^22^ estimated that in 2019, contract pharmacy arrangements generated $5 billion in profit from 340B sales.

Investigations highlighted the dramatic increase in pharmacy participation since 2010. Although previous guidance only permitted a single contract pharmacy,^42^ HRSA advised in 2010 that covered entities could use an unlimited number of contract pharmacies.^44^ As a result, the number of contract pharmacies working with covered entities increased from approximately 1000 in 2010 to almost 28 000 in 2021.^19^ In 2017, approximately 25% of US pharmacies participated in the 340B program,^45^ with the 5 largest pharmacy chains accounting for 60% of contract pharmacies.^26^ As of July that year, the number of contract pharmacies employed by individual covered entities ranged from 0 to 439.^26^ The average among all covered entities using at least 1 contract pharmacy was 12, whereas the average among disproportionate share hospitals was 25.

The location of contract pharmacies varied widely. Although many contract pharmacies were within 30 miles of the covered entity in 2017, a GAO report^26^ found that 45% of disproportionate-share hospitals engaged a contract pharmacy more than 1000 miles away. One study^45^ of contract pharmacies found many stationed in higher-income, less diverse neighborhoods. Another study^46^ found that contract pharmacies for safety net clinics were opening in poorer communities, whereas the locations of contract pharmacies for 340B hospitals were uncorrelated with rates of poverty or uninsurance. A third study^47^ found that contract pharmacies were more prevalent in poorer communities but less prevalent in communities with high uninsurance rates.

The types of 340B discounted drugs dispensed by contract pharmacies differed from all prescriptions dispensed by pharmacies. For example, 1 study^48^ found that 340B prescriptions dispensed by contract pharmacies had a higher share of antivirals and specialty medicines and a lower share of generic drugs.

### Pharmaceutical Manufacturers

Pharmaceutical manufacturers face high costs through participation in the 340B program because they are required to provide steep discounts on their drugs to levels far below private market prices. In 2020, manufacturers sold more than $80 billion in drugs (or 16% of manufacturer US sales) at 340B discounted prices of approximately $38 billion.^49,50^ Manufacturers have tried to limit the scope of the program, and in turn the amount of their 340B discounted sales, by challenging regulations implemented by HRSA and placing restrictions on their participation with contract pharmacies.^51,52^

The 340B program’s effects on drug pricing remain unresolved. One study uncovered no data supporting an association between 340B discounts and related inflation penalties with manufacturer price increases in Medicare Part D.^53^ A separate study^54^ calculated that a 60% reduction in the list prices of hepatitis C drugs may have actually saved manufacturers $182 million from lower 340B discounts, whereas another suggested that the 340B program may have contributed to a 10% annual increase in list prices of cancer drugs between 1995 and 2013.^55^ Still, to our knowledge, no study investigated the association between the 340B program and manufacturers’ drug pricing practices broadly.

### Patients

Patients benefited from the 340B program through the programs and health care services that covered entities provided to them. Surveys revealed that some covered entities used 340B funds to open specialty clinics, dispense free or low-cost medications, offer patients transportation, and provide patient education services.^29,36^ However, 1 study^56^ found no relationship between the 340B program and increased provision of such services to low-income patients. Little research was identified on the diversity of patients in the 340B program or the benefits of the program to racial and ethnic minority groups.

There was mixed evidence on the association between the 340B program and patient cost savings. Some studies showed that some patients received free or low-cost medications from covered entities or contract pharmacies.^26^ A 2012 study^57^ comparing uninsured patients’ prescription drug costs and savings related to patient assistance programs and the 340B program at 2 community health centers found that patients receiving 340B medications had an average medication cost of $11.50 and average savings of $62.31 relative to list prices. However, in a GAO survey of 55 covered entities, 25 reported that they did not offer discounts at their contract pharmacies.^26^ Another study^58^ found that out-of-pocket costs increased for patients paying cash at 340B covered entities.

The association of the 340B program with patient outcomes was also mixed. One study^59^ found an association between the 340B program and increased medication adherence: 340B clinics had 5% higher medication adherence for patients with diabetes and 3% higher for patients with hyperlipidemia and hypertension compared with the general patient population, and 340B hospitals had 7% higher medication adherence for patients with diabetes, 6% higher for patients with hyperlipidemia, and 5% higher for patients with hypertension. However, a different study^56^ of 340B-eligible disproportionate share hospitals found no relationship between 340B program eligibility and mortality rates.

## Discussion

Our scoping review revealed that the 340B program has grown substantially since it was launched and provided meaningful benefits to covered entities, pharmacies, and patients. Covered entities used revenue from the 340B program to expand health care services and programming, open specialty clinics, provide medications at reduced costs to patients, and subsidize uncompensated care and staff salaries. Patients of covered entities received greater access to health care services, but there was mixed evidence as to lower medication costs. However, covered entities—notably disproportionate share hospitals—also used 340B revenue for purposes seemingly unrelated to underserved patient care, including opening sites in higher-income neighborhoods and acquiring outpatient physician practices.

Pharmaceutical manufacturers, meanwhile, missed out on revenue as a result of the 340B program and pursued several legal challenges against it. Most recently, manufacturers challenged the HRSA mandate that manufacturers deliver 340B drugs to contract pharmacies.^52^ District courts have reached different conclusions,^60,61,62,63^ and there has been only 1 appeals court ruling thus far, supporting manufacturer restrictions on 340B drug sales.^64^ Since then, at least 20 manufacturers have set conditions on their deliveries to contract pharmacies, although with other cases still pending, the propriety of these moves remains a source of legal uncertainty.^65^

The findings of this study demonstrate that the 340B program offers value to many stakeholders in the US health care system. Studies have shown that many covered entities used 340B revenue to provide additional health services to patients, subsidize uncompensated and charity care, and provide free or low-cost medications to patients. These findings should be considered against the increasing criticism of the 340B program. The benefits from the 340B program may vary based on the category of the covered entity. In particular, federally-funded clinics and disproportionate share hospitals likely benefit in different ways, with clinics seeming more reliant on 340B revenue to stay open and disproportionate share hospitals using 340B revenue to expand health care services. Still, our findings show that the 340B program has been successful in aiding safety net hospitals and clinics serving low-income and underserved populations and that the consequences of eliminating or substantially restricting the program would be great.

Even with the strengths of the program, our review identified facets of the 340B program for potential reform. Covered entities are financially benefitting from the 340B program, yet some hospitals may be operating inconsistently with its goals. There are no requirements on how covered entities spend their 340B revenue, and it is difficult to study these activities and evaluate their effects. Covered entities’ use of 340B funds has been a controversial area that received pushback from the federal government.^66,67,68^ In 2017, the Centers for Medicare & Medicaid Services announced that it would decrease Medicare Part B reimbursement for 340B hospitals from average sales price plus 6% to average sales price minus 22.5% to account for discounts received under the 340B program. However, in 2022, the Supreme Court rescinded the rule,^69^ and a federal court required repayment to the hospitals at the higher reimbursement rate.^70^ Studies also conflict on the extent of patient financial benefits, particularly on whether 340B discounts are passed on to patients or are benefiting covered entities in unintended ways.^26,29,36,56,57,58,59^ These critiques are more concerning in the context of audits showing duplicate discounting and diversion.^40,41^ As the 340B program grows, involving more covered entities and contract pharmacies and reaching more patients, the need for additional data reporting and oversight is critical.

One avenue for reform would be new legislation requiring all covered entities to face greater transparency requirements. Federal grantees currently have some reporting conditions, including how they spend grant funds and data on the clients serviced and services provided.^71^ Of specific concern are disproportionate share hospitals, which have increasingly served higher-income communities and have been criticized for their practices.^23,24^ At a minimum, all covered entities should be required to report to HRSA data on 340B revenue and their spending to expand health care service offerings and programming. Additional requirements could be set for the proportion of 340B revenue that must be put toward community benefit spending. These rules will promote trust and accountability in the 340B program and support future evaluations of its successes and effectiveness. For example, data on use of 340B funds can inform rules on spending of 340B revenue or changes in the calculation of 340B discounts. Congress should also delegate HRSA additional rulemaking and enforcement authority to strengthen its administration and oversight of the 340B program. This authority would bolster the ability of HRSA to clarify program requirements and address compliance violations. It would additionally limit the need for Congressional intervention in the future.

### Limitations

This study was limited by a lack of critical information on the 340B program, such as pricing, savings, and revenue, which were confidential, proprietary, or unavailable. Most literature focused on 340B disproportionate share hospitals, with less research on federal grantees and nonhospital covered entities. Greater attention is needed on the effects of the 340B program on these 340B-covered entities. Inherent limitations in scoping review methodology should also be noted.^16,72^ The study did not formally evaluate the quality of the evidence, identify potential biases in the individual or collective studies, or address the relative weight of the evidence in presenting the findings. Further, scoping reviews focus on breadth rather than depth on a particular topic. However, this method was most appropriate given our objectives to provide an overview of several aspects of the 340B program with analyses from several perspectives.

## Conclusions

In this scoping review of the 340B program, we found evidence that the 340B program benefited hospitals, clinics, pharmacies, and patients, with notable costs to pharmaceutical manufacturers. Increased transparency regarding the use of 340B program revenue and strengthened rulemaking and enforcement authority for HRSA would support compliance and help ensure the 340B program achieves its intended purposes.

## Notes

Supplement 1. **eAppendix 1.** Detailed Methodology
eReferences
**eTable 1.** Database Search Terms **eTable 2.** All Documents Included in the Scoping Review

Supplement 2.
Data Sharing Statement
