# Error in Supplement

**DOI:** 10.1001/jamahealthforum.2024.3404

**Published:** 2024-09-27

The Original Investigation “Outcomes of the 340B Drug Pricing Program: A Scoping Review,”^1^ published November 22, 2024, included errors in Supplement 1. In Supplement 1, the PRISMA extension for Scoping Reviews checklist (eTable 3) has been removed. This article has been corrected.^1^
