# Supplementary material for: Outcomes of the 340B Drug Pricing Program: A Scoping Review
Source: JAMA Health Forum. 2023 Nov 22;4(11):e233716. doi: 10.1001/jamahealthforum.2023.3716 (PMC10665972; doi:10.1001/jamahealthforum.2023.3716)
Supplement: Supplement 1. — eAppendix 1. Detailed Methodology eReferences eTable 1. Database Search Terms eTable 2. All Documents Included in the Scoping Review [file jamahealthforum-e233716-s001.pdf]

# Supplemental Online Content

Knox RP, Wang J, Feldman WB, Kesselheim AS, Sarpatwari A. Outcomes of the 340B Drug Pricing Program: a scoping review. *JAMA Health Forum*. 2023;4(10):e233716. doi:10.1001/jamahealthforum.2023.3716

**eAppendix 1.** Detailed Methodology
**eReferences**
**eTable 1.** Database Search Terms
**eTable 2.** All Documents Included in the Scoping Review

This supplemental material has been provided by the authors to give readers additional information about their work.

© 2023 Knox RP et al. *JAMA Health Forum.*

**eAppendix 1. Detailed Methodology**

In order to gain a broad understanding of the history and evolution of the 340B Drug Pricing Program and to assess the outcomes and impact of the 340B Program since its implementation, we conducted a scoping review. Our study followed the scoping review methodology set forth by Arskey and O'Malley[1] and the Preferred Reporting Items for Systematic reviews and Meta-Analyses extension for Scoping Reviews (PRISMA-ScR) checklist.[2]

We selected several databases to review literature from several disciplines, including medicine, health services, law, economics, and public health. We used PubMed, a National Center for Biotechnology Information database of biomedical and life sciences articles; Embase, a database of biomedical research maintained by Elsevier; EconLit, an academic database of economic articles maintained by the American Economic Association; NBER, a National Bureau of Economic Research database of working papers in economics; and Westlaw, a legal research database published by Thomas Reuters. We additionally searched Google, the Government Accountability Office (GAO) website, and the Department of Health and Human Services Office of the Inspector General (HHS-OIG) website. Search terms varied by database and included variations of *340B*, *340B Drug Pricing Program*, *340B Drug Discount Program*, and *340B Program*. Search terms for each database and dates of initial and final searches are included in **Appendix 1 Table 1**.We conducted initial searches in May 2022. Searches were updated iteratively through February 1, 2023. Duplicates of retrieved articles, non-English language articles, and articles published prior to 1992 were removed. The titles and abstracts of remaining articles were independently reviewed by two team members (RK and JW for PubMed, Embase, EconLit, NBER, Google, GAO, and HHS-OIG; RK and AS for Westlaw) applying the following

© 2023 Knox RP et al. *JAMA Health Forum.*

exclusion criteria: (1) articles with no reference to the 340B Program in the title or abstract, (2) errata publications, (3) Federal Register publications soliciting comments, (4) Federal Register delay notices, (5) articles providing stakeholders guidance on 340B Program compliance, and (6) articles in which reference to the 340B Program was tangential to the article's focus (e.g., a study of family planning clinics that briefly mentions their eligibility for 340B discounts). Discordant categorizations for inclusion were resolved by discussion and involved a full-text review of the article.

The titles and abstracts of remaining articles were independently reviewed for inclusion by two authors. For all included articles, we recorded the (1) author, publication year, and publication type; (2) study objective or article thesis; (3) stakeholders discussed; (4) results or analyses; (5) conclusions; and (6) limitations (**eTable 2**).

Consistent with a scoping review, a wide range of document types were included in addition to articles from peer-reviewed literature, such as law review articles, white papers from the grey literature, reports published by government agencies (e.g., HHS-OIG, GAO, and the Congressional Research Service), Congressional committee reports and transcripts, opinion pieces, blog posts, and webpages. These articles provided a breadth of perspectives in evaluating the 340B Program. Government reports indicated the intent of the 340B Program and how agencies have measured the impact and successes of the program. Law review articles, white papers from the grey literature, and less formal analyses from blog posts and web pages supplemented the peer-reviewed literature and provided more varied expertises and perspectives.

© 2023 Knox RP et al. *JAMA Health Forum.*

**eReferences**

1. Arksey H, O'Malley L. Scoping studies: towards amethodological framework. *Int J Soc Res Methodol*. 2005;8(1):19-32. doi:10.1080/1364557032000119616

2. Tricco AC, Lillie E, ZarinW, et al. PRISMA extension for scoping reviews (PRISMA-ScR): checklist and explanation. *Ann Intern Med*. 2018;169(7):467-473. doi:10.7326/M18-0850

© 2023 Knox RP et al. *JAMA Health Forum.*

## eTable 1. Database Search Terms

| Database | Access Dates (First and Last) | Search String | Total Unique Records Retrieved | Total Included (duplicates excluded) |
|---|---|---|---|---|
| **PubMed** | 5/02/2022<br><br>2/1/2023 | ((340b) AND ((drug pricing program) OR (drug discount program) OR (discount* outpatient drug*))) | 103 | 83 |
| **EMBASE** | 5/02/2022<br><br>2/1/2023 | 340b:ab,ti AND ('drug discount program':ab,ti OR 'drug pricing program':ab,ti OR 'discount* outpatient drug*':ab,ti) | 93 | 12 |
| **EconLit** | 5/02/2022<br><br>2/1/2023 | 340B | 4 | 2 |
| **NBER** | 5/02/2022<br><br>2/1/2023 | 340B, "Working Paper," 1992-Present | 12 | 1 |
| **Westlaw: Secondary Sources – Law Reviews and Journals** | 5/02/2022<br><br>2/1/2023 | "340B" | 177 | 28 |
| **Westlaw: Legislative History Tab for 42 U.S.C. § 256b** | 5/02/2022<br><br>2/1/2023 | "340B" OR "drug"* | 183 | 23 |
| **Google** | 5/02/2022<br><br>2/1/2023 | "340B drug discount program" OR "340B program" OR "340B drug pricing program"<br><br>With advanced Google search filters: Language [English]; Region [United States]; Safe Search [On]; Publication Date [1/1/1991 to present] | 209 | 103 |
| **GAO** | 5/02/2022<br><br>2/1/2023 | "340B" – Reports only | 22 | 11 |
| **HHS-OIG** | 5/02/2022<br><br>2/1/2023 | "340B" – All Reports | 97 | 26 |
| | | | **900** | **289** |

© 2023 Knox RP et al. *JAMA Health Forum.*

## eTable 2. All Documents Included in the Scoping Review

| Article Citation | Article Type | Study Objective/Article Thesis | Stakeholders Discussed | Results/Analyses | Conclusions/ Recommendations | Limitations |
|---|---|---|---|---|---|---|
| **PubMed** | | | | | | |
| Albrecht J, Mudahar S. The Reach of the 340B Drug Pricing Program. *JAMA Dermatol*. 2015;151(9):923-924. doi:10.1001/jamadermatol.2015.1005 | Opinion | To describe the impact of the 340B Program on dermatology | Hospitals, manufacturers, pharmacies | 340B Programs have the potential to generate revenue for hospitals and dermatologists may be able to use the program to help uninsured patients. | Dermatologists should become aware of the 340B Program to benefit their patients. | N/A |
| Alpert A, Hsi H, Jacobson M. Evaluating The Role Of Payment Policy In Driving Vertical Integration In The Oncology Market. *Health Aff (Millwood)*. 2017;36(4):680-688. doi:10.1377/hlthaff.2016.0830 | Original Research Article | To study vertical integration in the oncology market ad to assess the role of Medicare reimbursement and the 340B Program | Hospitals | "Difference-in-difference analyses yielded statistically insignificant results in the rate of hospital or health system ownership for practices in counties with hospitals newly eligible for the 340B program, relative to practices in counties without any new eligible hospitals…. Medicare Part B payment reform and 340B Drug Discount Program expansion under the ACA are unlikely to have been primary factor(s) driving the recent wave of vertical integration in the oncology market." | The study concluded that "neither changes in Medicare payment policy for chemotherapy drugs nor the expansion in eligibility for the 340B Drug Discount Program under the ACA are the primary causes for the recent wave of oncology practice consolidation with hospitals and health systems. Instead, vertical integration in the oncology market may be part of a broader trend occurring across medical specialties." | The study's data was not designed for research purposes and the study only included hospitals newly eligible for the 340B Program after the Affordable Care Act. |
| American Society of Clinical Oncology. Policy statement on the 340B Drug Pricing Program by the American Society of Clinical Oncology. *J Oncol Pract*. 2014;10(4):259-263. | Opinion | To provide a summary of issues and recommendations related to the 340B Program from the perspective of oncologists for policymakers to consider | Hospitals | ASCO recommended reforms to the 340B Program focused on the original intent of the program in helping low-income populations, patient access to care and medicines, and the definition of a patient under the 340B Program. | N/A | N/A |

© 2023 Knox RP et al. *JAMA Health Forum.*

| Article Citation | Article Type | Study Objective/Article Thesis | Stakeholders Discussed | Results/Analyses | Conclusions/ Recommendations | Limitations |
|---|---|---|---|---|---|---|
| Bach PB, Sachs RE. Expansion of the Medicare 340B Payment Program: Hospital Participation, Prescribing Patterns and Reimbursement, and Legal Challenges. *JAMA*. 2018;320(22):2311-2312. doi:10.1001/jama.2018.15667 | Commentary | To describe the first stage of the litigation challenging Medicare's decreasing the reimbursement to 340B covered entities. | Hospitals, clinics, manufacturers | The authors criticize the 340B Program eligibility requirements, as they are based on inpatient services but applies to drugs dispensed to outpatients. Hospital expansion into higher income areas is inconsistent with the purpose of the 340B Program. CMS' proposed reimbursement cut for 340B hospitals might ameliorate a small distortion the program has caused, but could also adversely affect those facilities. | "The next chapter in the 340B story will be a return to court, as the AHA has refiled its lawsuit challenging the cuts in reimbursement. Thus, the long history of the controversial 340B program will continue to unfold and could have important national implications." | N/A |
| Barlas S. The 340B Discount Drug Program At Ozarks Medical Center: An Economic Stimulus Plan for Missouri. *P T*. 2011;36(5):280-281. | News | To describe how the 340B drug discount program will supplement future revenue lost due a cut in Missouri's Medicaid funding | Hospital | A rural hospital in Missouri was suffered financially from a cut from state 2011 Medicaid funding, the recession, charity care, and contractual loss built a network of outpatient contract pharmacies to generate a new revenue stream. The new contract pharmacy network not only served as an economic stimulus program, but also contributed to overall community health and clinic value in terms of patient adherence. | 340B programs, and in particular using contract pharmacy networks, pose an opportunity for eligible entities to increase their revenues. | N/A |

© 2023 Knox RP et al. *JAMA Health Forum.*

| Article Citation | Article Type | Study Objective/Article Thesis | Stakeholders Discussed | Results/Analyses | Conclusions/ Recommendations | Limitations |
|---|---|---|---|---|---|---|
| Barlas S. Congress Likely to Rein In 340B Drug Discount Program: The HRSA's Draft Guidance and a Proposed Rule Give Legislators an Opening. *P T*. 2015;40(10):656-667. | News | To describe and provide commentary on Congress' intention to rein in the scope of the 340B Program | Hospitals, clinics, manufacturers, federal government | About one third of hospitals use the 340B Program. Medicare Part B spending his higher at 340B hospitals than non-340B hospitals. Lack of price transparency, lack transparency on the use of funds, and an unclear definition of patient all have caused criticism of the program. The budget for the 340B Program was $1.5 billion in 2009. The lack of resources hampers HRSA's oversight of the program. HRSA also lacks authority to rectify many of these issues. | As HRSA doesn't have the legal authority to respond to programmatic weakness, any proposed restrictions in the draft guidance on patients eligible for 340B drugs wouldn't be enforced unless Congress encodes the changes into law. | N/A |
| Barlas S. More Clouds Form Over 340B Program: Potential Medicare Cut Underlines Need to Rein In Program. *P T*. 2017;42(10):628-631. | News | To discuss the proposed cuts to Medicare Part B reimbursement of 340B hospitals | Hospitals, clinics, manufacturers, federal government | The CMS wants to exact a severe reduction in Medicare reimbursement from ASP + 6% to ASP 22.5% to 340B hospitals for 340B drugs. CMS and lawmakers suggest the cuts may address some program weaknesses. Hospital industry representatives say the cut will harm hospitals caring for the sickest, most vulnerable patients. | N/A | N/A |

© 2023 Knox RP et al. *JAMA Health Forum.*

| **Article Citation** | **Article Type** | **Study Objective/Article Thesis** | **Stakeholders Discussed** | **Results/Analyses** | **Conclusions/ Recommendations** | **Limitations** |
|---|---|---|---|---|---|---|
| Bhatt J, Orlowski JM. Consequences of the 340B Drug Pricing Program. *N Engl J Med*. 2018;378(21):2053. doi:10.1056/NEJMc1802999 | Medical Journal Reply | To respond to Desai & McWilliams study | Hospitals | The authors argued that there is an error in the regression, asserted the study did not control for differences in hospital scale<br><br>Analysis is limited to Medicare beneficiaries and does not take into account Medicaid beneficiaries and other low-income patients that are supposed to benefit from the 340B program<br><br>Study findings cannot be extrapolated because only 20% of 340B hospitals were included in the study | N/A | Authors of the reply are from the American Hospital Association and the Association of American Medical Colleges, who are challenging Medicare's decrease in reimbursement for 340B drugs – which this study provides evidence in support of. |

© 2023 Knox RP et al. *JAMA Health Forum.*

| Article Citation | Article Type | Study Objective/Article Thesis | Stakeholders Discussed | Results/Analyses | Conclusions/ Recommendations | Limitations |
|---|---|---|---|---|---|---|
| Bidwal M, Lor K, Yu J, Ip E. Evaluation of asthma medication adherence rates and strategies to improve adherence in the underserved population at a Federally Qualified Health Center. *Res Social Adm Pharm*. 2017;13(4):759-766. doi:10.1016/j.sapharm.2016.07.007 | Empirical Study | To "Assess asthma medication adherence and determine predictors of non-adherence in the underserved population at a FQHC." | Clinics, patients | "Approximately one-third of individuals were identified with medium–high adherence to asthma medications, of which only 8.3% of individuals were found to be fully adherent (MPR >= 0.8). The majority of individuals (66.1%) were identified with low adherence, despite efforts to reduce medication adherence barriers associated with drug cost, access to care, and language. Patients with low adherence were younger, had fewer medication refills, had fewer primary care provider visits, lower baseline Asthma Control Test (ACT) scores, and lower asthma medication ratios (AMR) than patients with medium–high adherence." "Despite being an FQHC providing low cost medications in a culturally competent manner, the majority of patients (Hispanic and non-Hispanic) presenting to this clinic were non-adherent." | "To improve asthma medication adherence rates, Community Health Clinic Ole should include consistent use three strategies: healthcare team support, optimal medication counseling, and patient-centered communication strategies."<br><br>"Our findings demonstrate that asthma medication adherence remains poor among all underserved patients despite improved access to care via reduced medication pricing and the provision of Spanish-speaking medication consultations at refill pick-ups. Poor adherence rates remained common among both the Non-Hispanic and Hispanic, younger, and lower-socioeconomic patients in our study." | 1) "the study was conducted in a single institution serving primarily low-income underserved patients and results may not be generalizable to the general asthma population"<br>2) "a cross- sectional study design only measures adherence at one point in time. This does not allow for the assessment of the variation of adherence over the full length of treatment"<br>3) "adherence measured by an MPR is a secondary measure of adherence; it cannot be confirmed whether patients actually used their inhalers as prescribed with precise technique"<br>4) "the study took place in an FQHC and may not be generalizable to other healthcare settings" |

© 2023 Knox RP et al. *JAMA Health Forum.*

| Article Citation | Article Type | Study Objective/Article Thesis | Stakeholders Discussed | Results/Analyses | Conclusions/ Recommendations | Limitations |
|---|---|---|---|---|---|---|
| Boerner H. (2015). 340B: major changes may be coming to the federal drug discount program for low-income and vulnerable populations. *Physician Leadersh J.* 2015;2(6):42-44. | News | To review new guidance proposed for 340B hospitals | Hospitals, manufacturers, federal government | HRSA in 2015 August proposed new rules would:<br>1) "keep all 340B-qualifying hospitals and satellite locations on a public list"<br>2) "Require all hospitals and satellite sites that want to be included in the program to provide documentation that they fit the criteria of the program."<br>3) "Require private, not-for-profit hospitals to have a signed certification from a governor or other official"<br>4) Limit who can prescribe 340B drugs by limiting the prescribing ability to providers employed by or under contract with a 340B enrolled hospital, not an affiliated physician<br>5) "Prohibit hospitals from using 340B drugs for patients being discharged from the hospital"<br>6) "Require all 340B-qualifying entities to notify HHS immediately if they no longer fit the criteria." | N/A | N/A |

© 2023 Knox RP et al. *JAMA Health Forum.*

| Article Citation | Article Type | Study Objective/Article Thesis | Stakeholders Discussed | Results/Analyses | Conclusions/ Recommendations | Limitations |
|---|---|---|---|---|---|---|
| Bright DR, Adams AJ, Akala FO, Lengel AJ, Martin SJ, Powers MF. Implementation of a $4 generic drug program in a 340B pharmacy. *Am J Health Syst Pharm*. 2010;67(11):929-931. doi:10.2146/ajhp090269 | Case study | To determine the initial effectiveness of a $4 generic medication program by examining the number of pharmacy patients and the number of prescriptions filled at the CMHC pharmacy | Clinic, pharmacies, patients | 1) "The total number of prescriptions filled at the CMHC pharmacy from January to March 2009 was 7134. For the same three-month period in 2008, 6166 prescriptions were filled."<br>2) "In the first three months after implementation of the program (January–March 2009), the average monthly prescription volume (in number of prescriptions filled) increased by 9 prescriptions per day in January, by 19 prescriptions per day in February, and by 23 prescriptions per day in March." | "A $4 generic drug program implemented at a 340B pharmacy increased patients' access to medication and increased the pharmacy's prescription volume while contributing to an increase in the pharmacy's net revenue." | N/A |
| Castellon YM, Bazargan-Hejazi S, Masatsugu M, Contreras R. The impact of patient assistance programs and the 340B Drug Pricing Program on medication cost. *Am J Manag Care*. 2014;20(2):146-150. | Original Research | The objective was "to examine the financial impact that PAPs and the 340B Program have on improving medication cost." | Patients | For patients receiving PAP medications the average cost of medications per visit was $0.11 (95% CI, $0.04-$0.17) and average savings per visit was $617.36 (95% CI, $581.32-$653.40). For patients receiving 340B medications the average cost of medications per visit was $11.50 (95% CI, $10.55-$12.45). Average savings on medications per visit was $62.31 (95% CI, $57.99-$66.63). | While savings for 340B medications were less than those for PAPs, the 340B program allows for purchasing both generic prescription drugs and OTC drugs. In addition, there were almost twice as many 340B medications dispensed in the month of February, providing increased access to medications for patients who may not otherwise have been able to afford their medications. Both 340B and PAPs were successful in providing significant medication cost savings for patients. | 1) cost savings were calculated using average market value versus other more affordable alternatives;<br>2) drug pricing may not represent what patients would paid; not a longitudinal study due to fluctuating market;<br>3) did not focus on cost-effective for the safety net clinic. |

© 2023 Knox RP et al. *JAMA Health Forum.*

| Article Citation | Article Type | Study Objective/Article Thesis | Stakeholders Discussed | Results/Analyses | Conclusions/ Recommendations | Limitations |
|---|---|---|---|---|---|---|
| Challen L, Kelso C, Pautler HM, Benanti G. Diabetes Outcomes: Comparison of Patient Assistance Programs to 340B Drug Pricing. *J Health Care Poor Underserved*. 2016;27(3):1192-1198. doi:10.1353/hpu.2016.0108 | Original research | "To determine whether primary care medicine clinic (PCMC) patients who receive insulin from manufacturer- sponsored PAPs have an improved change in A1C, compared with patients who receive the same medications through the 340B Drug Pricing Program." | Hospitals, patients | "The baseline and change in A1C for patients enrolled in a PAP was similar to patients receiving 340B pricing (10.3% vs. 9.3%) (– 0.52 ±2.67 vs. – 0.3 ±2.32, p=.66). Baseline and changes in SCr (1.0 vs. 0.99) (0.08 ±0.26 vs. 0.08 ±0.40, p=.93) and body mass index (34.0 vs. 33.9) (0.15 ±2.29 vs. 0.10 ±2.16, p=.89) were also similar between both groups. PAP patients and 340B patients had a similar number of hospital admissions (1.23 ±2.08 vs. 1.48 ±2.66, p=.612). Similarities were also noted with number of anti- hyperglycemic medications prescribed (2.04 ±0.72 vs. 1.96 ±0.76, p=.59) and number of PCMC physician visits (7.59 ±5.16 vs. 340B 7.82 ±5.73, p=.83)" | There was no significant difference in baseline and change in diabetes outcomes, number of medication use, healthcare utilization, and adherence rates between PAP group and 340B group. | 1) small sample size<br>2) short study duration<br>3) no information on important variables (e.g., length of diagnosis/ prior insulin use) |

© 2023 Knox RP et al. *JAMA Health Forum.*

| Article Citation | Article Type | Study Objective/Article Thesis | Stakeholders Discussed | Results/Analyses | Conclusions/ Recommendations | Limitations |
|---|---|---|---|---|---|---|
| Chapman R. A different view of the 340B program. *Oncology (Williston Park)*. 2014;28(3):178. | Commentary | To examine critiques of the 340B Program | Hospitals | 1) 340B program has "enabled vulnerable patients to access high-quality, affordable, convenient care", has "has enabled (oncologists) to (...) providing care for the underserved", has "helped offset a fraction of the cost of uncompensated care that (oncologists) provide to poor and uninsured Detroiters".<br>2) "(The recommendation) to limit the program only to indigent patients or redefining it as a patient entitlement would only boost Big Pharma's already healthy profit margins while leaving safety-net providers with fewer resources to carry out their missions."<br>3) Because of the 340B program, patients can now access their medications "closer to home through contracts with community drugstores", and receive oncology treatment and follow-up care near their home. | "Rather than place blame on a very important program, we should work together to address the most pressing challenges facing our community--declining reimbursement, high drug costs, and the need for more investments in cancer research and prevention." | N/A |

© 2023 Knox RP et al. *JAMA Health Forum.*

| Article Citation | Article Type | Study Objective/Article Thesis | Stakeholders Discussed | Results/Analyses | Conclusions/ Recommendations | Limitations |
|---|---|---|---|---|---|---|
| Clark BL, Hou J, Chou CH, Huang ES, Conti R. The 340B discount program: outpatient prescription dispensing patterns through contract pharmacies in 2012. *Health Aff (Millwood)*. 2014;33(11):2012-2017. doi:10.1377/hlthaff.2014.0833 | Original Research | To: 1) Compare national 340B-qualified prescriptions dispensed by Walgreens (a 340B contract pharmacies and the largest national pharmacy chain) and all prescriptions dispensed by this vendor; and 2) Describe the medical facilities that originated the 340B-qualified prescriptions. | Pharmacies, patients | Medications used to treat chronic disease (diabetes, high cholesterol levels, asthma, depression) account for an overwhelming majority of all prescriptions diseased through the 340B program. A higher percentage of antiretrovirals used to treat HIV/AIDS were dispensed through 340B prescriptions than through all prescriptions dispensed at Walgreens. The majority of 340B prescriptions dispensed at Walgreens originated at tuberculosis clinics, consolidated health centers, disproportionate-share hospitals, and Ryan White clinics. | "Patient assistance programs and discounted 340B medication pricing are two means for providing affordable medication to primary care patients without prescription drug benefits. Though in this study we found no statistical differences in diabetes outcomes or adherence of patients receiving insulin through a PAP or 340B pricing, we hope this study may serve as a platform for future research, which is promising. Larger sample sizes and a longer study duration may lead to more information regarding the association between the outcomes investigated. Furthermore, inquiry into patient perspectives and the effect of PAP or 340B pricing on quality of life may provide institutions with additional insight in the benefits of offering such programs to their patients." | 1) small sample size 2) short study duration 3) no information on important variables (e.g., length of diagnosis/ prior insulin use) |
| Cole J, Gardiner J, Curfman G. US Supreme Court Review of the 340B Drug Discount Program-American Hospital Association v Becerra. *JAMA Health Forum*. 2022 Mar 1;3(3):e215210. doi: 10.1001/jamahealthforum.2021.5210. PMID: 36218870. | Commentary | To discuss ongoing litigation related to the 340B Program | Hospitals, clinics, pharmacies | The Supreme Court heard arguments in *AHA v. Becerra* on whether the Medicare statute permitted differential reimbursement to 340B hospitals without pricing surveys conducted by HHS. There is also litigation challenging covered entities' use of contract pharmacies. | The future of the 340B Program will be greatly affected by these cases. | N/A |

© 2023 Knox RP et al. *JAMA Health Forum.*

| Article Citation | Article Type | Study Objective/Article Thesis | Stakeholders Discussed | Results/Analyses | Conclusions/ Recommendations | Limitations |
|---|---|---|---|---|---|---|
| Conti RM, Bach PB. Cost consequences of the 340B drug discount program. *JAMA*. 2013;309(19):1995-1996. doi:10.1001/jama.2013.4156 | Commentary | To describe the paradoxical effects of the 340B program on the costs of patient care | Hospitals, clinics, manufacturers, patients | The 340B program may be having paradoxical effects on the costs of patient care, in particular for patients with cancer, because:<br>1) availability of profits from prescribing expensive cancer drugs alters physician prescribing behavior; 2) disparity between 340B and non-340B entities in the profits they obtain from the care of insured cancer patients may lead to consolidation and affiliations between community-based oncology practices and 340B-eligible hospitals; shifting of care out of community-based oncology practices and into hospital-based infusion suites; 3) drug manufacturers will likely seek to increase list prices even further to offset revenue losses incurred as a larger number of drug sales become eligible for 340B discounts (and thus fewer drugs are sold at full price). | 340B program may be reconfigured to hospitals serving the most vulnerable patients while eliminating its cost-increasing effects by:<br>1) limit hospitals and their affiliated contract pharmacies to providing the 340B discounted drugs only to the poor and uninsured<br>2) require hospitals and treating physicians to pass on their savings from drug purchases to patients and their insurance providers, including Medicare; or allow insurers to recoup some 340B profits from hospitals and physicians and pass those profits back to their beneficiaries.<br><br>Most of the costs of the program are borne by manufacturers; the financial benefits of the 340B discounts are accruing almost entirely to hospitals, clinics, and physicians; and patients' out-of- pocket costs and total cost of care are being increased. | N/A |

© 2023 Knox RP et al. *JAMA Health Forum.*

| Article Citation | Article Type | Study Objective/Article Thesis | Stakeholders Discussed | Results/Analyses | Conclusions/ Recommendations | Limitations |
|---|---|---|---|---|---|---|
| Conti RM, Bach PB. The 340B drug discount program: hospitals generate profits by expanding to reach more affluent communities. *Health Aff (Millwood)*. 2014;33(10):1786-1792. doi:10.1377/hlthaff.2014.0540 | Original Research | To "examine whether the program is expanding in ways that could maximize hospitals' ability to generate profits from the 340B drug discounts." | Hospitals | 1) The number of newly registered 340B DSH hospitals began to increase at a higher rate starting in 2003<br>2) Hospital-affiliated clinics that registered for the 340B program in 2004 or later served communities that were wealthier and had higher rates of health insurance compared to communities served by hospitals and clinics that registered for the program before 2004<br>3) Compared to 340B DSH hospitals, their affiliated clinics tended to serve communities with lower poverty rates and higher mean and median income levels than their 340B DSH hospital parents did. | 1) The expansions among 340B DSH hospitals run counter to the program's original intention.<br>2) Gaining access to 340B drug discounts may act as one motivating rationale for the affiliations and mergers among hospitals and outpatient physician practices that are becoming increasingly common in the United States.<br><br>The 340B Program is being converted from one that serves vulnerable patient populations to one that enriches hospitals and their affiliated clinics | The study looked at a single year of data. There was also no breakdown of the makeup of the population that a 340B entity serves. |

© 2023 Knox RP et al. *JAMA Health Forum.*

| Article Citation | Article Type | Study Objective/Article Thesis | Stakeholders Discussed | Results/Analyses | Conclusions/ Recommendations | Limitations |
|---|---|---|---|---|---|---|
| Conti RM, Jones DK. Policy Diffusion across Disparate Disciplines: Private- and Public-Sector Dynamics Affecting State-Level Adoption of the ACA. *J Health Polit Policy Law*. 2017;42(2):377-385. doi:10.1215/03616878-3766771 | Law Journal Article | To examine how state-level adoption of the Affordable Care Act was affected by various public and private programs and policies. | Hospitals | "(340B program) was intended to provide assistance to medical providers who serve poor, underinsured patients, and is a good example of how federal policy impacts the private market, which in turn impacts state policy."<br><br>"340B-qualified hospitals are expanding their base into communities that tend to be affluent and well-insured, consistent with the most profitable expansion strategy that counters the objectives of the program (...) These activities drive up costs of providing care — and ultimately, commercial insurance premiums—since hospital outpatient contracts tend to be much more generous than physician office contracts and charge facility fees on top of service charges to payers and patients." | "Future empirical work on the rate, determinants, and impacts of ACA coverage expansions on individual and aggregate well-being must incorporate systematic study of this complex public–private sector ecosystem." | N/A |

© 2023 Knox RP et al. *JAMA Health Forum.*

| Article Citation | Article Type | Study Objective/Article Thesis | Stakeholders Discussed | Results/Analyses | Conclusions/ Recommendations | Limitations |
|---|---|---|---|---|---|---|
| Conti RM, Nikpay SS, Buntin MB. Revenues and Profits From Medicare Patients in Hospitals Participating in the 340B Drug Discount Program, 2013-2016. *JAMA Netw Open*. 2019;2(10):e1914141. Published 2019 Oct 2. doi:10.1001/jamanetworkopen.2019.14141 | Original research | "To report revenues and estimated profits from the 340B program that hospitals collect from Medicare and Medicare beneficiaries for outpatient clinic administration of prescription drugs covered under Medicare Part B." | Hospitals, clinics | "This cross-sectional study found that, in 2016, hospitals received \$3.7 billion for the administration of drugs in the 340B discount program; assuming a 50% discount, profits totaled \$1.9 billion. Median 340B profits from Medicare were 0.3% of hospital operating budgets and 9.4% of hospital uncompensated care costs. Revenues and estimated profits from administering drugs in the 340B discount program to Medicare beneficiaries are small in terms of overall hospital budgets but substantial compared with uncompensated care costs. Revenues and estimated profits from administering drugs in the 340B discount program to Medicare beneficiaries are small in terms of overall hospital budgets but substantial compared with uncompensated care costs." | "Estimated profits that hospitals derived from administering 340B-discounted drugs to Medicare patients are small compared with operating budgets yet substantial compared with uncompensated care costs for many hospitals."<br>"We found that acute care nonprofit and public hospitals and drugs covered by Medicare Part B are increasingly eligible for 340B discounts, and revenues and estimated profits from Medicare patients for hospitals participating in the 340B program are large and increasing over time. Our results suggest that the Office of the Inspector General's focus on oncology drugs alone in a previous report underestimates 340B revenues and profits that hospitals make from Medicare Part B reimbursements. … Moreover, our results suggest that there is substantial variability in the benefits of participation in the340B program nationwide and by state." | "One important limitation of our study is that we did not have access to claims for drugs dispensed by outpatient pharmacies that were eligible for 340B discounts by participating hospitals and their contract pharmacy relationships."<br>"we did not have access to claims for drugs dispensed by outpatient pharmacies that were eligible for 340B discounts by participating hospitals and their contract pharmacy arrangements… we did not have access to drugs administered by physicians on an outpatient basis that are eligible for 340B discounts by hospitals reimbursed by commercial insurers and their beneficiaries." |

© 2023 Knox RP et al. *JAMA Health Forum.*

| Article Citation | Article Type | Study Objective/Article Thesis | Stakeholders Discussed | Results/Analyses | Conclusions/ Recommendations | Limitations |
|---|---|---|---|---|---|---|
| Coukell AJ, Dickson S. Reforming the 340B Drug Pricing Program: Tradeoffs Between Hospital and Manufacturer Revenues [published correction appears in JAMA Intern Med. 2018 Aug 1;178(8):1144]. *JAMA Intern Med*. 2018;178(8):1127-1128. doi:10.1001/jamainternmed.2018.2007 | Commentary | To evaluate proposals to reform the 340B Program, focusing on hospital and manufacturer revenues | Hospitals, manufacturers | 1) "Policy proposals requiring that 340B savings be allocated directly to outpatients or outpatient services could decrease the ability of 340B hospitals to fund low-profit inpatient services."<br>2) Late 340B entrants "might be expected to have lower levels of uncompensated care and higher operating margins than early entrants to the 340B program; they still, however, provide more uncompensated care and have lower operating margins than non-340B hospitals".<br>3) "The net effect (of 2018 Medicare reimbursement cut) would be to transfer Medicare spending from the 340B hospital to the pharmaceutical manufacturer. Similarly, any narrowing of 340B eligibility to a smaller set of qualifying institutions would transfer the corresponding share of government payment for drugs from the hospital or the clinic to the manufacturer, (increasing manufacturers' revenues)." | "the public and policy makers would be well served by greater transparency on hospitals' use of 340B revenues, as suggested by lawmakers. Because any reduction in 340B eligibility increases manufacturer revenues, increased transparency will help policy makers characterize the benefits to patients when considering tradeoffs between hospital and manufacturer revenues from any changes to the program." | N/A |

© 2023 Knox RP et al. *JAMA Health Forum.*

| Article Citation | Article Type | Study Objective/Article Thesis | Stakeholders Discussed | Results/Analyses | Conclusions/ Recommendations | Limitations |
|---|---|---|---|---|---|---|
| Curfman G, Cole J. The 340B Drug Discount Program Preserved After US Supreme Court Review-But Chevron Remains Vulnerable. JAMA Health Forum. 2022 Sep 2;3(9):e223185. doi: 10.1001/jamahealthforum.2022.3185. PMID: 36218943. | Commentary | To analyze the Supreme Court's opinion in *AHA v. Becerra* | Hospitals, federal government | "The Court ruled that the text of the statute, as stipulated by the US Congress, is clear: if HHS does not conduct surveys on drug costs, it must follow reimbursement option 2, which does not permit variation of reimbursement according to hospital group. Thus, the Court concluded that the modified reimbursement formula used by HHS for 340B-covered entities was in violation of the clear language of the statute. The Court held that instead, 340B institutions must be reimbursed according to the average sales price of the drugs purchased plus 6%, meaning that they will be able to recuperate the large financial losses accrued since 2018." | "Because of the Court's opinion in American Hospital Association v Becerra, 340B-covered entities may now breathe a sigh of relief. However, also as a consequence of this case and West Virginia, federal agencies (including health care agencies) that have relied on Chevron deference to resolve legal disputes in their favor may have effectively lost this important doctrinal tool. The American Hospital Association v Becerra case is a distinctive example of how a seemingly minor and technical Supreme Court decision may nonetheless have extraordinarily important implications for US society and the law." | N/A |
| Daifotis AG. Risks to the 340B Drug Pricing Program. *JAMA*. 2022 Sep 20;328(11):1109. doi: 10.1001/jama.2022.12753. PMID: 36125477. | Medical Journal – Reply | To reply to Knox et al.'s Viewpoint on 340B contract pharmacy litigation | Hospitals, clinics, pharmacies | The reply criticized the Viewpoint's characterization of manufacturer restrictions on contract pharmacy agreements and called for greater transparency in the 340B Program. | N/A | N/A |

© 2023 Knox RP et al. *JAMA Health Forum.*

| Article Citation | Article Type | Study Objective/Article Thesis | Stakeholders Discussed | Results/Analyses | Conclusions/ Recommendations | Limitations |
|---|---|---|---|---|---|---|
| Desai SM, McWilliams JM. 340B Drug Pricing Program and hospital provision of uncompensated care. *Am J Manag Care*. 2021 Oct;27(10):432-437. doi: 10.37765/ajmc.2021.88761. PMID: 34668672; PMCID: PMC8544813. | Original Research | "To evaluate whether hospital entry into the 340B Drug Pricing Program, which entitles eligible hospitals to discounts on drug purchases and intends for hospitals to use associated savings to devote more resources to the care of low-income populations, is associated with changes in hospital provision of uncompensated care." | Hospitals, patients | "We do not find evidence that hospitals increased provision of uncompensated care after entry into the 340B program differentially more than hospitals that never entered or had not yet entered the program." | "Relying on hospitals to invest surplus into care for the underserved without marginal incentives to do so or strong oversight may not be an effective strategy to expand safety-net care." | 1) The analysis does not look at critical access hospitals.<br>2) The analysis does not consider hospitals joining for financial reasons to maintain current levels of uncompensated care.<br>3) The analysis did not control for predictors of uncompensated care that changed after hospitals joined the 340B Program. |

© 2023 Knox RP et al. *JAMA Health Forum.*

| Article Citation | Article Type | Study Objective/Article Thesis | Stakeholders Discussed | Results/Analyses | Conclusions/ Recommendations | Limitations |
|---|---|---|---|---|---|---|
| Desai S, McWilliams JM. Consequences of the 340B Drug Pricing Program. *N Engl J Med*. 2018;378(6):539-548. doi:10.1056/NEJMsa1706475 | Original Research | "The primary objective of this study was to assess the extent to which hospitals have followed program incentives by acquiring practices or employing more physicians in parenteral drug–intensive specialties, treating more patients in these specialties, and favoring high-income groups with more generous insurance when treating additional patients. The secondary objective was to test whether the program has been associated with expanded care or decreased mortality in low-income groups." | Hospitals, patients | "Hospital eligibility for the 340B Program was associated with 2.3 more hematologist–oncologists practicing in facilities owned by the hospital, or 230% more hematologist–oncologists than expected in the absence of the program (P=0.02), and with 0.9 (or 900%) more ophthalmologists per hospital (P=0.08) and 0.1 (or 33%) more rheumatologists per hospital (P=0.84). Program eligibility was associated with significantly higher numbers of parenteral drug claims billed by hospitals for Medicare patients in hematology–oncology (90% higher, P=0.001) and ophthalmology (177% higher, P=0.03) but not rheumatology (77% higher, P=0.12). Program eligibility was associated with lower proportions of low-income patients in hematology–oncology and ophthalmology and with no significant differences in hospital provision of safety-net or inpatient care for low-income groups or in mortality among low-income residents of the hospitals' local service areas." | "The 340B Program has been associated with hospital–physician consolidation in hematology–oncology and with more hospital-based administration of parenteral drugs in hematology–oncology and ophthalmology. Financial gains for hospitals have not been associated with clear evidence of expanded care or lower mortality among low-income patients." | 1) Reliance on Medicare Data<br>2) "program-related increases in hospital ownership of physician practices could have been overstated if practices owned by hospitals merely changed place-of-service codes to allow administration of discounted drugs"<br>3) "r regression-discontinuity approach supported inferences about hospitals just above the eligibility thresholds"<br>4) "conclusions may not apply to categories of eligible hospitals we did not study, such as critical access hospitals" |

© 2023 Knox RP et al. *JAMA Health Forum.*

| Article Citation | Article Type | Study Objective/Article Thesis | Stakeholders Discussed | Results/Analyses | Conclusions/ Recommendations | Limitations |
|---|---|---|---|---|---|---|
| Desai S, McWilliams JM. Consequences of the 340B Drug Pricing Program. *N Engl J Med*. 2018;378(21):2053-2054. doi:10.1056/NEJMc1802999 | Medical Journal - Reply | To respond to Reply Letters to Desai & McWilliams article | Hospitals, patients | 1) "our findings pertain directly to hospitals near the eligibility threshold, may not generalize to hospitals with DSH percentages that are well above the threshold, and do not exclude the beneficial use of drug discounts by individual hospitals. Nevertheless, our findings — which were based on an analysis of hospitals accounting for more than 50% of Medicare spending on care in public and nonprofit general acute care hospitals — constitute strong evidence of hospital responses that are inconsistent with the goals of the 340B Program and thus provide support for reform of this program." 2) "We reject the notion that low-income Medicare beneficiaries do not compose a population intended to benefit from the 340B Program." 3) "none of the points raised by Bhatt and Orlowski are of great consequence, let alone "undermine" our findings. Our conclusions remain unaltered." | N/A | N/A |

© 2023 Knox RP et al. *JAMA Health Forum.*

| Article Citation | Article Type | Study Objective/Article Thesis | Stakeholders Discussed | Results/Analyses | Conclusions/ Recommendations | Limitations |
|---|---|---|---|---|---|---|
| Dickson S. Association Between the Percentage of US Drug Sales Subject to Inflation Penalties and the Extent of Drug Price Increases [published correction appears in JAMA Netw Open. 2020 Oct 1;3(10):e2026259]. *JAMA Netw Open*. 2020;3(9):e2016388. Published 2020 Sep 1. doi:10.1001/jamanetworkopen.2020.16388 | Original Research | To "Estimate the association of existing inflation penalties in the US 340B Drug Pricing Program with manufacturer pricing behaviour in the Medicare Part D program and associated changes in Medicare pharmacy expenditures" | Manufacturers | "A higher sales percentage subject to inflation penalties was associated with lower annual price increases. Lower price increases owing to inflation penalties were estimated to be associated with a reduction in Medicare Part D pharmacy expenditures of $7.1 billion between 2013 and 2017."<br>1) "The data in the present analysis indicated that, although drugs with substantial sales percentages subject to inflation penalties had lower price increases compared with other drugs, their price increases remained higher than the rate of inflation. This finding suggests that the inflation penalty present in the 340B program may not sufficiently counteract other manufacturer incentives to increase prices."<br>2) "An inflation penalty will be most successful in discouraging price increases when a manufacturer is required to pay both the inflation penalty and the PBM price protection rebate, ensuring that price increases are actually associated with decreases in the manufacturer's net revenues." | "In this cross-sectional study, increases in the percentage of drug sales subject to inflation penalties were associated with lower annual price increases. Broader application of inflation penalties may help to reduce drug price increases and decrease overall drug spending." | 1) "errors could have occurred in the data-matching process, and not all prescriptions by physicians in a 340B-eligible organization may be filled in the 340B program<br>2) "Because this study used Medicare prescribing data only, the findings may not reflect the total sales percentage subject to inflation penalties for a given drug if non-Medicare drug use were to substantially differ based on the prescribing site (and therefore 340B eligibility)."<br>3) "This study did not consider new brand-name drug competition as a separate confounding variable in the model." |

© 2023 Knox RP et al. *JAMA Health Forum.*

| Article Citation | Article Type | Study Objective/Article Thesis | Stakeholders Discussed | Results/Analyses | Conclusions/ Recommendations | Limitations |
|---|---|---|---|---|---|---|
| Dickson S, Reynolds I. Estimated Changes in Manufacturer and Health Care Organization Revenue Following List Price Reductions for Hepatitis C Treatments. *JAMA Netw Open*. 2019;2(7):e196541. Published 2019 Jul 3. doi:10.1001/jamanetworkopen.2019.6541 | Original Research | "To estimate the differences in manufacturer and health care organization revenue from the Medicare Part D program following list price reductions for hepatitis C treatments, accounting for manufacturer discounts to eligible health care organizations under the 340B drug discount program and manufacturer rebates to pharmacy benefit managers." | Hospitals, manufacturers | 1) "The 3 hepatitis C treatments evaluated had 30% to 41% of claims prescribed by 340B-eligible health care organizations, greater than the 14% 340B prescribing rate for all Medicare Part D drugs."<br>2) "Based on use data from 2016, list price reductions for (the 3 selected) hepatitis C treatments in 2018 were estimated to have increased aggregate manufacturer net revenues for 3 treatments by $181.9 million—a 28% increase."<br>3) "Following the price change, aggregate 340B health care organization net revenues were estimated to have been $181.9 million lower—a 74% decrease."<br>4) "Net costs to Medicare and beneficiaries remained constant" | "Given the high share of 340B sales within the HCV market, reducing a drug's list price generates greater net manufacturer revenues than the same price reduction via a rebate mechanism (...) because with a high list price, the manufacturer would have to pay both a PBM rebate and a higher 340B discount, but with a lower list price and no rebate, the manufacturer faces only a lower 340B discount."<br>"List price reductions for hepatitis C treatments may have increased drug manufacturer net revenues, owing in part to lower discounts provided under the 340B program and the high share of sales subject to those discounts."<br>"Policymakers should consider the role of 340B discounts when evaluating policies to reduce drug spending", as "reduc(ing) the size of the 340B program may therefore diminish the 340B program's potential to restrain price increases or encourage list price decreases." | 1) There could be some "errors in the matching process to identify 340B-eligible prescribers, and not all prescriptions by 340B-eligible prescribers may be filled under the 340B program."<br>2) "The Medicare data set used only includes prescribers with 11 or more claims for a particular drug".<br>3) "Medicare beneficiaries may see different prescribers than non-Medicare patients, and estimates of the share of 340B-eligible prescriptions for a therapeutic class or individual product may not reflect the 340B-eligible share in the broader market" |

© 2023 Knox RP et al. *JAMA Health Forum.*

| Article Citation | Article Type | Study Objective/Article Thesis | Stakeholders Discussed | Results/Analyses | Conclusions/ Recommendations | Limitations |
|---|---|---|---|---|---|---|
| Eagle D, Buell RL, Vacirca J. The 340b drug discount program: oncology's optical illusion. *Oncology (Williston Park)*. 2013;27(11):1068-1072. | Opinion | To describe the impact of the 340B program on oncology practice and spending. | Hospitals, patients | "the 340B program creates a substantial discrepancy between the economic viability of eligible hospitals and that of other entities, including private oncology offices… If one type of care delivery system is financed more advantageously than another, it is predictable that the universe of oncology care will migrate towards the advantaged system. Additionally, the enormous profits derived from the 340B program create a large appetite among hospitals for the acquisition of private oncology clinics. Unfortunately, this migration from private practice to 340B institutions will cost both payers and patients more. For Medicare patients, oncology care in the hospital outpatient department costs 14% more than care delivered in a physician office. Patient out-of-pocket expenses are also higher in the former setting. For privately insured patients, hospital outpatient departments are 24% more costly than a physician office." | "While the 340b program should not be eliminated, it is ripe for reform. Allowing the drug discounts to be applied only to the indigent would seem sensible. Alternatively, building patient support programs around the patients themselves, rather than around institutions to which the patient may or may not access, would ensure that more patients in need are supported." | N /A |

© 2023 Knox RP et al. *JAMA Health Forum.*

| Article Citation | Article Type | Study Objective/Article Thesis | Stakeholders Discussed | Results/Analyses | Conclusions/ Recommendations | Limitations |
|---|---|---|---|---|---|---|
| Endriukaitis LA, Hayes GL, Mills J. Economic Evaluation of Changes in Reimbursement for Medications Purchased Through the 340B Drug Pricing Program. *Hosp Pharm*. 2021;56(4):235-240. doi:10.1177/0018578719888907 | Original Research | To review the financial impact of changes in the 2018 Medicare reimbursement for clinic-administered medications in a 340B hospital. | Hospitals | - "Most of the drugs (55%) (included in the analyses) were associated with oncologic indications. The actual reimbursement margin for 2018 was $3193525."<br>- "Changes to the reimbursement structure of the program has undoubtedly affected overall reimbursement. By our estimate, the overall medication reimbursement margin would have been $5.39 million dollars, conservatively, based on the previous scheme of ASP plus 6%, representing an almost $2.2 million dollar decreased payment in a single calendar year. "<br>- "An overall negative payment margin was present in 34 medications (39%), ranging from $−319558 to $−2.73. Negative variances were accounted for either through 1) billing discrepancies (eg, incorrect package size and billing units) (N=9) or 2) unfavorable 340B acquisition price relative to the published ASP (N=21). " | "Compounded with decreased Medicare payments, institutions providing uncompensated services may have difficulty spreading federal resources to populations in need" as many of the programs that improve health outcomes would run out of funding."<br><br>"Changes to reimbursement outlined by the CMS at the start of 2018 resulted in decreased reimbursement for 340B-eligible, clinic-administered medications. Most of the unfavorable variances were associated with 340B acquisition prices that exceeded reimbursement. Although the original intent of the 340B Drug Pricing Program was to stretch federal resources, decreased payments could reduce institutional ability to fund programs that support medically vulnerable populations." | 1) "did not account for medication-associated costs such as labor and materials", which may "further dimishing the potential for positive margin"<br>2) "limited to a single institution" and "singular reporting of the 340B drug"<br>3) the billing discrepancies observed in the study could be institution-specific<br>4) "didn't include medications that are administered directly to the site of disorder, (which) may underestimate or overestimate actual reimbursement margins"<br>5) "the status of the changes to 340B reimbursement outlined under the OPPS final rule is in a state of uncertainty" |

© 2023 Knox RP et al. *JAMA Health Forum.*

| Article Citation | Article Type | Study Objective/Article Thesis | Stakeholders Discussed | Results/Analyses | Conclusions/ Recommendations | Limitations |
|---|---|---|---|---|---|---|
| Fauer A, Friese CR. 340B Drug Pricing Program: The Expansion and Its Effect on Cancer Care. *Clin J Oncol Nurs*. 2019;23(2):217-219. doi:10.1188/19.CJON.217-219 | Commentary | To describe the effects of the 340B program on cancer care and provide guidance for nurses. | Hospitals, clinics | "Community oncology practices that are not eligible to participate in the 340B program have struggled to keep up with soaring prices of cancer drugs. Since 2008, 1,653 community oncology practices in the United States have closed, been acquired by hospital groups, or reported that they were struggling financially. With 340B program eligibility, hospitals that acquired a community oncology practice would receive the freestanding cancer hospital drug discount for purchased cancer drugs. Community oncology clinicians, including physicians, nurses, and pharmacists, are affected by care shifting away from community practices and toward hospital-based settings. Studies have demonstrated evidence of the 340B drug pricing program's impact on cancer care, such as vertical integration, biosimilar price competition, and profit generation. …. Taken together, the 340B Drug Pricing Program policy is a powerful contributor to the U.S. cancer care delivery system." | "Policymakers and clinicians, including physicians, nurses, and pharmacists, should explore solutions that allow for community oncology practices to receive drug discounts at similar levels to hospitals within the 340B Drug Pricing Program and continually study the effects of these solutions with clinician-led research." | N/A |

© 2023 Knox RP et al. *JAMA Health Forum.*

| Article Citation | Article Type | Study Objective/Article Thesis | Stakeholders Discussed | Results/Analyses | Conclusions/ Recommendations | Limitations |
|---|---|---|---|---|---|---|
| Fein AJ. Challenges for Managed Care from 340B Contract Pharmacies. *J Manag Care Spec Pharm*. 2016;22(3):197-203. doi:10.18553/jmcp.2016.22.3.197 | Opinion | To discuss crucial ways in which 340B growth is affecting managed care pharmacies | Hospitals, clinics, pharmacies | Challenges for managed care pharmacies include formulary rebates, profits from managed care paid prescription, disruption of managed care pharmacy networks, and reduced generic dispensing rates. | 1) "Managed care should become more engaged in discussions on how the 340B program evolves and offer policy proposals to mitigate the challenges it is encountering as a result of 340B program policies."<br>2) "There is an urgent need for objective, peer-reviewed research on the 340B program's costs, benefits, and implications for managed care pharmacy and practice." | N/A |
| Fong WH. 340B Drug Pricing Program Reform. *JAMA Oncol*. 2016;2(3):403. doi:10.1001/jamaoncol.2015.5400 | Medical Journal Reply | To reply to Kantarjian et al. | Hospitals, patients | Fong challenges authors' assertion that 340B is helping more patients and providing more services, instead arguing (1) patients are harmed because hospitals do not pass the discounts on to patients, (2) the standards for 340B participation have decreased and the program has expanded exponentially, and (3) consolidation of hospitals and community cancer centers has decreased the quality and convenience of cancer care. | "Congress needs to tighten up the 340B program's qualification standards so that only individual hospital sites that serve a substantial percentage of vulnerable patients participate in the 340B program. The 340B program should explicitly exclude the institution as a whole, their affiliates, and other sites that do not serve a high percentage of poor patients.340B Drug Pricing Program drugs should only go to 340B-eligible patients. Furthermore, 340B-eligible institutions should be legally required to pass along drug discounts to the target population." | N/A |

© 2023 Knox RP et al. *JAMA Health Forum.*

| Article Citation | Article Type | Study Objective/Article Thesis | Stakeholders Discussed | Results/Analyses | Conclusions/ Recommendations | Limitations |
|---|---|---|---|---|---|---|
| Genao I, Hendrickson K, Diers D, Browne R, Rawlings JE. Creating a medical home through the 340B Drug Pricing Program. *Conn Med*. 2010;74(10):615-620. | Original Research | To "Track inpatient and outpatient utilization before and after the implementation of the 340B Drug Pricing Program in a public university hospital." | Hospitals, patients | 1) "Our study results show a decrease in hospitalization rates, and an increase in utilization rates of primary-care services for both adults and children, HIV, ambulatory services, the ED, and some specialty services."<br>2) "Outpatient and emergency department utilization rates increased with small increments of direct cost."<br>3) "Inpatient utilization decreased while inpatient length of stay increased with over three million dollars in direct cost savings." | 1) "The provision of affordable medications leading to increase utilization of outpatient services, including the ED, suggest that many enrollees in the 340B Drug Pricing Program have chosen to make YNHH their medical home. (Moreover,) accessing primary-care clinics more often for routine care means medical conditions are diagnosed and treated earlier, potentially avoiding ancillary testing."<br>2) "The increased LOS (length of stay) could be attributed to the sicker patients being hospitalized, since those more clinically stable patients were cared for in outpatient settings."<br><br>"Improving continuity of care for patients without a usual source of care may have the effect of creating a “medical home.” The 340B Drug Pricing Program may be one policy tool to improve care for the underserved while increasing outpatient utilization and decreasing inpatient utilization and costs." | 1) "focused entirely in one hospital and our sample composed of patients who qualified (generally by income) for the 340B program"<br>2) "ould not match medical record numbers to all patients in the pharmacy database" |

© 2023 Knox RP et al. *JAMA Health Forum.*

| Article Citation | Article Type | Study Objective/Article Thesis | Stakeholders Discussed | Results/Analyses | Conclusions/ Recommendations | Limitations |
|---|---|---|---|---|---|---|
| Gerlach J, McSweeney S, Swearingen A, Coustasse A. Examining the Benefits of the 340b Drug Discount Program. *Health Care Manag (Frederick)*. 2018;37(3):225-231. doi:10.1097/HCM.0000000000000220 | Systematic review | To determine which stakeholders benefited because of the 340B Drug Discount Program and what have been the drivers of recent changes to the program | Hospitals, clinics, pharmacies, patients | "Considering outpatient prescription medicine caters toward a significant number of conditions that affect the low-income populations, discounts on such medications have allowed pharmacies to realize substantial cost savings as they would have purchased the drugs at a higher price if they opted out of the (340B) program."<br>"340B eligible hospitals have realized increased Medicare profits because of 340B."<br>"Through expansion of services (i.e., affiliations and mergers among hospitals and outpatient physician practices), hospitals have driven organic growth in 340B through increased volumes that would qualify for 340B savings."<br>"retail pharmacy contracts have allowed pharmacies to increase covered entities' participation in the 340B program and in return, the covered entity offered a per-prescription dispense fee that induced retail pharmacies to participate"<br>"The program has also improved access to medications used to treat chronic conditions such as diabetes and asthma among the indigents." | "As 340B has continued to expand, political advocacy has been successful in creating controversy over the direct financial benefit that health care providers and contract pharmacies have realized because of the program. However, vulnerable and underserved patient populations have also benefited in the form of expanded services and increased access to health care. This type of benefit, although indirect, has fulfilled the intent of the 340B program at its inception." | 1) "did not include primary research into how individual hospitals and other covered entities were using 340B drugs and accompanying profits in 2017"<br>2) "limited by the timeliness of the topic and the ongoing political and legal activity surrounding 340B"<br>3) "limited by the bias of the researchers, bias of the available publications, limitations of the chosen research strategy, and the number of databases used for the secondary research" |

© 2023 Knox RP et al. *JAMA Health Forum.*

| Article Citation | Article Type | Study Objective/Article Thesis | Stakeholders Discussed | Results/Analyses | Conclusions/ Recommendations | Limitations |
|---|---|---|---|---|---|---|
| Gricius RF, Wong D. Fast track to 340B. *Healthc Financ Manage*. 2016;70(1):56-62. | Commentary | To describe steps for newly eligible hospitals to fast track their approval to participate in the 340B program | Hospitals | With the expansion of Medicaid under the ACA, more hospitals will likely become eligible for the 340B program. | N/A | N/A |
| Hall CS. HIV Prevention and the 340B Drug Pricing Program. *N Engl J Med*. 2022 Aug 11;387(6):575-576. doi: 10.1056/NEJMc2208621 . PMID: 35947724. | Medical Journal Reply | To reply to Marcus et al.'s article | Clinics, patients | Hall argues for an "''all-of-the-above" financing strategy for maximizing engagement in PrEP is imperative, given the yawning gap in access to this prevention strategy." | N/A | N/A |
| Hardaway J. 340B Program Puts Manufacturers At Risk of Duplicate Drug Discounts. *P T*. 2016;41(1):38. | Letter to the Editor | To highlight the risk of duplicate discounting for pharmaceutical manufacturers | Hospitals, clinics, manufacturers | At least 25% of 340B Programs audited between 2012 and 2016 had duplicate discounting errors. HRSA does not have adequate guidance to prevent duplicate discounting errors in Medicaid managed care organizations and in contract pharmacy arrangements. | "My advice to manufacturers is simple: Understand your company's 340B duplicate discount risk and take steps to minimize it because the current program implementations fail to do so. Fortunately, program guidelines have repeatedly affirmed manufacturers' authority to review 340B program implementation, to dispute Medicaid rebate claims suspected of being erroneous, and to seek remediation if duplicate discount errors are discovered. Manufacturers must exercise this authority if they are to take control of their duplicate discount risk." | N/A |

© 2023 Knox RP et al. *JAMA Health Forum.*

| Article Citation | Article Type | Study Objective/Article Thesis | Stakeholders Discussed | Results/Analyses | Conclusions/ Recommendations | Limitations |
|---|---|---|---|---|---|---|
| Hart C. Protect the 340B drug program. *Mod Healthc*. 2014;44(7):24. | Opinion | To describe the value of the 340B Program | Hospitals, clinics, patients | "(340B) program is deeply important to urban, community and rural hospitals alike (…) patients receive free or low-cost medicines, as well as medication therapy management, and mental health and substance-abuse counseling."<br>Examples:<br>1) Black Hills of South Dakota: "340B savings help us provide free medications to uninsured patients upon discharge (...) including a large Native American population that does not have access to the Indian Health Service at night or on weekends."<br>2) Mount Sinai Health System in Chicago: "340B helps fund a clinic that treats stroke and brain-injury patients."<br>3) Monroe County Hospital in Monroeville, Ala.: " (340B) program funds low-cost or no-cost drugs for outpatients in its cancer and diabetes clinics" | "As a community of caregivers, I urge other (hospital) CEOs to rise and support the program publicly." | Author is a CEO of a hospital participating in the 340B Program |

© 2023 Knox RP et al. *JAMA Health Forum.*

| Article Citation | Article Type | Study Objective/Article Thesis | Stakeholders Discussed | Results/Analyses | Conclusions/ Recommendations | Limitations |
|---|---|---|---|---|---|---|
| Henkel B. What's at stake in effort to diminish 340B program. *Mod Healthc*. 2014;44(39):34. | Opinion | To discuss what is at risk if the 340B Program is downsized | Hospitals, clinics, patients | 1) "Because drug companies have become less willing to share their profits with those in need, access to the program is at risk."<br>2) "Participation from these hospitals (critical-access and disproportionate-share facilities) in 340B allowed us (non-profit health system) to save $97 million on drugs last year, which we reinvested in programs that treat the poor and vulnerable."<br>3) " The (340B) program cuts costs for a three-month supply of a cholesterol-lowering drug from $700 to just $60. A 15- day supply of an injectable form of insulin drops from over $400 to $16." | "340B is a vital, successful program that allows hospitals to help underserved patients with the high cost of drugs, and sustains medical services direly needed within the community. Any efforts to diminish 340B will only leave our most vulnerable citizens with less access to the medication and the quality healthcare they desperately need." | N/A |

© 2023 Knox RP et al. *JAMA Health Forum.*

| Article Citation | Article Type | Study Objective/Article Thesis | Stakeholders Discussed | Results/Analyses | Conclusions/ Recommendations | Limitations |
|---|---|---|---|---|---|---|
| Jones EA, Linas BP, Truong V, Burgess JF, Lasser KE. Budgetary impact analysis of a primary care-based hepatitis C treatment program: Effects of 340B Drug Pricing Program. *PLoS One*. 2019;14(3):e0213745. Published 2019 Mar 14. doi:10.1371/journal.pone.0213745 | Original Research | To "Conduct a budgetary impact analysis of a multidisciplinary primary care-based HCV treatment program, with and without 340B support." | Hospitals, patients | 1) "Total program costs were $942,770, while revenues totaled $1.2 million. With the 340B Drug Pricing Program the hospital received a net revenue of $930 per patient referred to the HCV treatment program."<br>2) "In the absence of the 340B program, the hospital would lose $370 per patient referred and would likely not be sustainable in resource-poor settings, which disproportionately care for HCV-infected patients."<br>3) "Ninety-seven percent (68/70) of patients who initiated treatment in the program achieved a sustained virologic response (SVR) at a net cost of $4,150 each, among this patient subset." | "The 340B Drug Pricing Program enabled a safety-net hospital to deliver effective primary care-based HCV treatment using a multidisciplinary care team. Efforts to sustain the 340B program could enable dissemination of similar HCV treatment models elsewhere." | 1) "conducted at a single site so may lack generalizability"<br>2) assumed fixed number of visits per patient, fixed costs for medications<br>3) did not consider costs related to space, capital equipment, or from the patient perspective<br>4) "insurers such as Medicaid may directly negotiate medication pricing with pharmaceutical companies and therefore the 340B drug discount program may not be applicable" |

© 2023 Knox RP et al. *JAMA Health Forum.*

| Article Citation | Article Type | Study Objective/Article Thesis | Stakeholders Discussed | Results/Analyses | Conclusions/ Recommendations | Limitations |
|---|---|---|---|---|---|---|
| Jung J, Xu WY, Kalidindi Y. Impact of the 340B Drug Pricing Program on Cancer Care Site and Spending in Medicare. *Health Serv Res*. 2018;53(5):3528-3548. doi:10.1111/1475-6773.12823 | Original Research | To "Examine the impact of the 340B drug discount program on the site of cancer drug administration and cancer care spending in Medicare." | Hospitals, patients | 1) "The probability of a patient receiving cancer drug administration in hospital outpatient departments (HOPDs) versus physician offices increased 7.8 percentage points more in new 340B markets than in markets with no 340B hospital."<br>2) "The program did not increase the probability of a patient receiving cancer drug administration and did not change the frequency of cancer drug claims among users."<br>3) "Per-patient spending on other cancer care increased $1,162 more in new 340B markets than in markets with no 340B hospital." | "No change in use/quantity led to the insignificant effect of 340B on cancer drug spending because Medicare pays the same for outpatient drugs regardless of setting."<br><br>Per-patient spending on cancer care increased could be because "patients visiting HOPDs for cancer drug administration may receive additional services that might not be offered if they visited physicians' offices" and "Medicare payments for those services are higher in HOPDs than in Offices. "<br><br>"The 340B program shifted the site of cancer drug administration to HOPDs and increased spending on other cancer care. As the program expands, continuing assessment of its impact on service utilization and spending would be needed." | 1) "did not examine pharmacy-dispensed cancer drugs, which are covered by Part D"<br>2) only included new 340B entities, so the results "may not be generalizable to the effects by 340B DSHs" or long-term impacts of the program<br>3) unmeasured time-varying confounding such as stage of cancer<br>4) "analysis is limited to cancer care in Medicare" |

© 2023 Knox RP et al. *JAMA Health Forum.*

| **Article Citation** | **Article Type** | **Study Objective/Article Thesis** | **Stakeholders Discussed** | **Results/Analyses** | **Conclusions/ Recommendations** | **Limitations** |
|---|---|---|---|---|---|---|
| Kantarjian H, Chapman R. 340B Drug Pricing Program Reform-Reply. *JAMA Oncol*. 2016;2(3):403-404. doi:10.1001/jamaoncol.2015.5403 | Commentary | To describe the value of the 340B program with a focus on oncology care | Hospitals, patients, pharmacies | 1) All hospitals are purchasing oncology practices.<br>2) The 340B Program continues to expand.<br>3) There are now more locations where 340B discounted drugs are dispensed, including contract pharmacies.<br>4) 340B sales will continue to increase in the future.<br>5) With Medicaid expansion, more hospitals will be eligible to participate in the 340B Program. | "Medical services and cancer care are undergoing fundamental changes that are not related to the 340B program and that would not be reversed by restricting 340B. Restricting 340B may worsen access to cancer care in rural areas by forcing the shutdown of rural centers and community hospitals that rely on such programs for financial solvency. Clarifying the root causes of this shift in the site of cancer care from community oncology to hospital-based practices, and addressing them with solutions and incentives that stabilize or reverse this trend (if judged beneficial to cancer care in the United States), is a better approach. Innovative solutions could also been visioned to create incentives for oncology practices outside hospital settings and in underserved areas, as well as piloting innovative telemedicine relations between rural medical practices and oncology specialists." | N/A |

© 2023 Knox RP et al. *JAMA Health Forum.*

| Article Citation | Article Type | Study Objective/Article Thesis | Stakeholders Discussed | Results/Analyses | Conclusions/ Recommendations | Limitations |
|---|---|---|---|---|---|---|
| Kantarjian H, Chapman R. Value of the 340B Drug Discount Program [published correction appears in JAMA Oncol. 2015 Nov;1(8):1172]. *JAMA Oncol*. 2015;1(8):1029-1030. doi:10.1001/jamaoncol.2015.2168 | Medical Journal – Reply | N/A | Hospitals, patients, pharmacies | The authors respond by saying (1) 340B hospitals provide more care to low income patients than non-340B hospitals, are 1/3 of DSH hospitals but provide 60% of uncompensated care, and provide more specialized, often unprofitable services than non 340B hospitals - arguing this shows that it is helping patients and increasing comprehensive care, and (2) it was Congress' intent for covered entities to profit from 340B drugs and not necessarily pass savings on to patients. | "The 340B program is essential to vulnerable patients with cancer and the 340B entities that support them. A disproportionate level of low-income patients are treated in 340B hospitals. The disproportionate amount of uncompensated care that 340B hospitals provide shows that they are de-serving of being 340B entities, and that they are using their program savings appropriately to further their mission of treating low-income, poor, and vulnerable patients." | N/A |

© 2023 Knox RP et al. *JAMA Health Forum.*

| Article Citation | Article Type | Study Objective/Article Thesis | Stakeholders Discussed | Results/Analyses | Conclusions/ Recommendations | Limitations |
|---|---|---|---|---|---|---|
| Kantarjian HM, Chapman R. Role of the 340B Drug Discount Program in Recent Cancer Care Trends. *J Oncol Pract*. 2015;11(4):303-307. doi:10.1200/JOP.2014.002139 | Review | "Review the role of the 340B drug discount program and its relevance to recent trends in cancer care and to the access of safety-net providers and vulnerable patients to much-needed drug support." | Hospitals, clinics, patients | While the number of hospital-based practices were increasing, the number of small private cancer care practices were decreasing between 2012 and 2013, presumably because "decreased profits may have the most impact on underserved areas, where oncology practices are smaller and less able to withstand financial pressures." | Proposed solutions to stop the trend include to:<br>1) "allow private community practices to qualify for 340B if they provide care to vulnerable patients in the outpatient setting that is equivalent, by objective measures, to the qualifications required for safety-net providers."<br>2) "modify the reimbursement model for chemotherapy delivered in an outpatient office, to account for the complexities of care."<br>3) "offer US citizenship to outstanding foreign graduate medical oncologists who complete their training in the United States and are willing to practice in underserved and rural areas for significant periods of time"<br>4) "moving away from fee-for-service reimbursement (which incentivizes overuse of medicines and increases health care costs) toward a bundled payment system that pays for episodes of care"<br>5) "create incentives for oncologists to practice outside hospital settings and in rural areas and pilot innovative telemedicine relationships between oncology specialists and rural medical practices." | N/A |

© 2023 Knox RP et al. *JAMA Health Forum.*

| Article Citation | Article Type | Study Objective/Article Thesis | Stakeholders Discussed | Results/Analyses | Conclusions/ Recommendations | Limitations |
| --- | --- | --- | --- | --- | --- | --- |
| Keough CL, Webster SA. 340B program presents opportunities--and challenges. *Healthc Financ Manage*. 2009;63(11):42-48. | Commentary | To advise hospitals on eligibility and compliance with the 340B program. | Hospitals | "The 340B program provides an opportunity for hospitals to achieve significant cost savings on outpatient drugs. Ongoing disputes surrounding the Medicare DSH payment calculation, and potential misunderstandings of the Medicare provider-based reimbursement rules, pose obstacles to hospitals that wish to continue or gain participation in the program. Eligible hospitals should pay close attention not only to the rule specifically governing the 340B program, but also to these Medicare reimbursement rules, to ensure their continued access to drug discounts available under the program." | N/A | N/A |

© 2023 Knox RP et al. *JAMA Health Forum.*

| Article Citation | Article Type | Study Objective/Article Thesis | Stakeholders Discussed | Results/Analyses | Conclusions/ Recommendations | Limitations |
|---|---|---|---|---|---|---|
| Knox RP, Kesselheim AS, Sarpatwari A. Risks to the 340B Drug Pricing Program Related to Manufacturer Restrictions on Drug Availability. *JAMA*. 2022 May 3;327(17):1647-1648. doi: 10.1001/jama.2022.5959. Erratum in: JAMA. 2022 Sep 20;328(11):1111. PMID: 35426909. | Commentary | To describe the litigation related to manufacturer restrictions on 340B contract pharmacies | Hospitals, clinics, pharmacies, manufacturers, patients | Six manufacturers sued HRSA regarding an HHS Advisory Opinion requiring manufacturers deliver 340B discounted drugs to an unlimited number of contract pharmacies and Violation Letters sent by HRSA to manufacturers related to their contract pharmacy restrictions. Courts have reached mixed results. "Two courts found that the procedures used by HRSA to challenge the manufacturers' restrictions were improper, while 1 court found HRSA had acted properly. Two courts held that the pharmaceutical manufacturers' restrictions of 340B drug sales to contract pharmacies were unlawful, whereas 2 courts found that the 340B statute did not clearly prohibit the manufacturers' restrictions. | "Two legislative steps could potentially help remedy this situation. First, Congress could explicitly allow the participation of contract pharmacies in the 340B program given their importance in securing revenue<br>For 340B centers and ensuring patient access to low-cost drugs… Second, to ensure the 340B program is functioning effectively, Congress could delegate rulemaking powers to HRSA to develop and enforce standards for preventing drug diversion and duplicate discounting." | N/A |
| Knox RP, Sarpatwari A. Risks to the 340B Drug Pricing Program-Reply. *JAMA*. 2022 Sep 20;328(11):1109-1110. doi: 10.1001/jama.2022.12756. PMID: 36125474. | Medical Journal Reply | To respond to Daifotis' letter replying to Knox et al.'s Viewpoint | Hospitals, clinics, pharmacies, patients | The author's critiqued Daifotis' characterization of contract pharmacy restrictions, the courts' opinions, and the necessity of contract pharmacies. | N/A | N/A |

© 2023 Knox RP et al. *JAMA Health Forum.*

| Article Citation | Article Type | Study Objective/Article Thesis | Stakeholders Discussed | Results/Analyses | Conclusions/ Recommendations | Limitations |
|---|---|---|---|---|---|---|
| Lee CH, Chang J, McCombs J. Specialty Drug Price Trends in the Federal 340B Drug Discount Program. *J Manag Care Spec Pharm*. 2019;25(2):178-187. doi:10.18553/jmcp.2019.25.2.178 | Original Research | "To analyze trends over a 10-year period (2006~2016) in the price of specialty drugs, contrasting the market price with the price paid under the 340B program." | Hospitals, clinics, manufacturers | "The 340B price growth rate patterns were similar to the profile of the WAC prices over time across all drug classes. The overall drug price growth rate per year over 10 years for WAC prices was 15% and 10% for 340B prices. For specialty drug classes, the average growth rates per year were 14% for the WAC price and 6% for the 340B price. For certain specialty drug classes, such as antineoplastic and antiretroviral drugs, the 340B price inflation rates were significantly lower than the WAC price inflation rates after 2013." | "The price inflation of specialty drugs exceeds the rate of inflation in the Consumer Price Index for prescription drugs. The 340B price shows a similar inflation pattern as the WAC price over time in the specialty drug categories." | 1) Study did not encompass all specialty drugs.<br>2) The study may not be generalizable to other drug classification systems (used AHFS Pharmacologic-Therapeutic Classification codes).<br>3) Drug class codes may change over years.<br>4_ Authors limited the study to real-world drug purchasing decisions rather than total drug catalog files of wholesalers. |

© 2023 Knox RP et al. *JAMA Health Forum.*

| Article Citation | Article Type | Study Objective/Article Thesis | Stakeholders Discussed | Results/Analyses | Conclusions/ Recommendations | Limitations |
|---|---|---|---|---|---|---|
| Li Y, Xu S. Association of Beneficiary-Level Risk Factors and Hospital-Level Characteristics With Medicare Part B Drug Spending Differences Between 340B and Non-340B Hospitals. JAMA Netw Open. 2022 Feb 1;5(2):e220045. doi: 10.1001/jamanetworkopen.2022.0045. Erratum in: JAMA Netw Open. 2022 Jul 1;5(7):e2224469. PMID: 35179584; PMCID: PMC8857681. | Original Research | "To examine whether per-beneficiary Medicare Part B drug spending is significantly different between 340B and non-340B hospitals while adequately controlling for patient-level and hospital-level risk factors." | Hospitals, patients | "The sample included 35 364 beneficiaries (21 825 women [61.7%]; 29 996 White patients [84.8%]; mean [SD] age, 70.6 [12.0] years) and 2446 hospitals. A total of 918 hospitals (37.5%) were in the 340B program and 938 hospitals (38.3%) were teaching hospitals. There was a higher percentage of teaching hospitals among 340B hospitals (517 of 918 [56.3%]) than non-340B hospitals (421 of 1528 [27.6%]), and beneficiaries who went to 340B hospitals were more likely to be non-White than those who went to non-340B hospitals (3360 of 19 139 [17.6%] vs 1583 of 13 710 [11.5%]). The Part B drug spending difference between 340B and non-340B hospitals was not statistically significant after controlling for beneficiary-level risk factors and hospital-level characteristics ($568; 95% CI, -$283 to $1419; P = .19)." | "The results show that the differences in patient population and hospital-level characteristics may explain drug spending differences between 340B and non-340B hospitals, which raises doubt about the financial incentive theory of the 340B program drug discount and the justification for the Centers for Medicare & Medicaid Services's 340B payment policy." | 1) Not all relevant detailed patient risk factors are available in Medicare claims data 2) Potential selection bias due to inherent differences between 340B and non-340B hospitals 3) Could not measure 340B financial incentives 4) Further research needed on relationship between 340B discounts and policies with physician prescribing behavior |

© 2023 Knox RP et al. *JAMA Health Forum.*

| Article Citation | Article Type | Study Objective/Article Thesis | Stakeholders Discussed | Results/Analyses | Conclusions/ Recommendations | Limitations |
|---|---|---|---|---|---|---|
| Lin JK, Li P, Doshi JA, Desai SM. Assessment of US Pharmacies Contracted With Health Care Institutions Under the 340B Drug Pricing Program by Neighborhood Socioeconomic Characteristics. *JAMA Health Forum*. 2022 Jun 17;3(6):e221435. doi: 10.1001/jamahealthforum.2022.1435. PMID: 35977245; PMCID: PMC9206190. | Original Research | To measure "pharmacy participation following the 2010 expansion and the extent to which growth has occurred in socioeconomically disadvantaged neighborhoods." | Hospitals, clinics, pharmacies | "From 2011 to 2019, the share of 340B retail pharmacies in socioeconomically disadvantaged and primarily non-Hispanic Black and Hispanic/Latino neighborhoods declined, even though the share of all retail pharmacies (ie, 340B and non-340B) in socioeconomically disadvantaged and racial and ethnic minoritized neighborhoods increased slightly… Although the percentage of 340B pharmacies in the lowest income neighborhoods declined by 5.6%, the percentage of non-340B pharmacies in the same neighborhoods increased by 1.3%. In contrast, though the percentage of 340B pharmacies in the highest income neighborhoods increased by 5.0%, the percentage of non-340B pharmacies in the same neighborhoods increased by 1.5%. There were similar differential declines in the percentage of 340B and non-340B pharmacies in neighborhoods with the highest levels of social deprivation, predominantly Black neighborhoods, and predominantly Hispanic/Latino neighborhoods." | "Substantial growth occurred following the 2010 340B expansion—by 2019, nearly one-third of all pharmacies were contracting with a 340B institution. The vast majority were retail pharmacies.<br><br>Contract pharmacy growth was concentrated in affluent and predominantly White neighborhoods, whereas the share of 340B pharmacies in socioeconomically disadvantaged and primarily non-Hispanic Black and Hispanic/Latino neighborhoods declined. Our study was limited in that we could not observe whether 340B discounts were passed to low-income patients. Nonetheless, our work adds to a growing body of evidence questioning the degree to which 340B program growth serves vulnerable communities." | N/A |

© 2023 Knox RP et al. *JAMA Health Forum.*

| Article Citation | Article Type | Study Objective/Article Thesis | Stakeholders Discussed | Results/Analyses | Conclusions/ Recommendations | Limitations |
|---|---|---|---|---|---|---|
| Malouin RA, Mckernan L, Forsberg A, et al. Impact of the 340B Pharmacy Program on Services and Supports for Persons Served by Hemophilia Treatment Centers in the United States. *Matern Child Health J*. 2018;22(9):1240-1246. doi:10.1007/s10995-018-2545-7 | Original Research | "Describe the impact of the 340B program funding on services and support provided by Hemophilia Treatment Centers (HTCs) to persons affected by rare bleeding disorders." | Clinics, patients | 1) "The 31 of 37 HTCs responding served over 10,000 individuals, or one-third of the national HTC patient population. The majority of responding HTCs reported that 340B program income supported over 90% of staff such as nurses, social workers, and physical therapists." 2) "Over 3000 patients at 25 centers received financial assistance for transportation, with almost half the centers reporting > 90% funding from 340B program income to help patients access care. " | "The results from this survey of 31 centers with established programs demonstrates the HTCs' reliance on 340B program support for vital comprehensive services, that are otherwise non-reimbursable, and highlights the importance of the 340B program in sustaining the high quality of care and in increasing access for a geographically dispersed, medically vulnerable population." | 1) "the survey over-represents the larger, more established (HTC) programs" 2) "the results of this survey represent care received by approximately one-third of the HTC population" 3) "inability to collect patient specific data from HTCs" 4) "collected information on ranges of funding support instead of exact amounts" |
| Mansour A. Built for the road ahead. *Healthc Financ Manage*. 2015;69(10):76-81. | Commentary | To describe how Henry Ford Health System in Detroit used the 340B program and patient assistance programs to improve medication adherence, reduce admissions, and achieve cost savings. | Hospitals | "The 340B program helped cover uncompensated drug costs, run a prescription assistance program, reduced preventable admissions; the hospital also requested additional scrutiny and clarity in the program" | N/A | N/A |
| Marcus JL, Killelea A, Krakower DS. HIV Prevention and the 340B Drug Pricing Program. Reply. N Engl J Med. 2022 Aug 11;387(6):576. doi: 10.1056/NEJMc2208621 . PMID: 35947725. | Medical Journal – Reply | To respond to Hall's reply to the authors' article | Clinics, patients | The authors argue that a national PrEP program needs to address the overreliance on the 340B Program in HIV/AIDS care. | N/A | N/A |

© 2023 Knox RP et al. *JAMA Health Forum.*

| Article Citation | Article Type | Study Objective/Article Thesis | Stakeholders Discussed | Results/Analyses | Conclusions/ Recommendations | Limitations |
|---|---|---|---|---|---|---|
| Marcus JL, Killelea A, Krakower DS. Perverse Incentives - HIV Prevention and the 340B Drug Pricing Program. N Engl J Med. 2022 Jun 2;386(22):2064-2066. doi: 10.1056/NEJMp2200601. Epub 2022 May 28. PMID: 35621521. | Commentary | To describe the perverse incentives in HIV/AIDS care related to the 340B Program | Clinics, patients | The 340B Program incentivizes the prescribing of hire-priced PrEP, even when there are cheaper and generic options available. The overreliance on the 340B Program should be addressed in reforms to HIV/AIDS care and the establishment of a national PrEP Program. | "The United States aims to decrease HIV transmission by 90% by 2030, but reaching that goal will require an overhaul of the HIV-prevention financing infrastructure. Although 340B will continue to facilitate access to discounted drugs, a national PrEP program would ensure that overreliance on 340B does not dictate clinical decision making. A decade after the emergence of PrEP as a powerful HIV-prevention tool, it's time to ensure widespread availability by promoting access to safe, effective, and sustainably priced medications, rather than providing incentives to prescribe higher-cost alternatives." | N/A |

© 2023 Knox RP et al. *JAMA Health Forum.*

| Article Citation | Article Type | Study Objective/Article Thesis | Stakeholders Discussed | Results/Analyses | Conclusions/ Recommendations | Limitations |
|---|---|---|---|---|---|---|
| Mulligan K, Romley JA, Myerson R. Access to the 340B Drug Pricing Program: is there evidence of strategic hospital behavior?. *BMC Res Notes*. 2021;14(1):228. Published 2021 Jun 3. doi:10.1186/s13104-021-05642-4 | Original Research | The article "Assessed the plausibility of strategic behavior to gain access to the 340B program by comparing the distribution of hospital DSH near the eligibility cutoff to what would be expected by chance alone, in the absence of strategic behavior." | Hospitals | 1) "In 2014–2016, the number of hospitals just above the threshold increases by 41% compared to the number just below it. Prior to 2014, this pattern was much less pronounced."<br>2) "McCrary density tests found this increase to be statistically significant across a range of bandwidths in 2014–2016 ($p < 0.01$). From 2011–2013, the findings are sensitive to the bandwidth around the threshold, but insignificant in 2008–2010. We found no comparable change among hospitals ineligible for the 340B program. " | These patterns "are consistent with a hypothesis that some hospitals adjusted their DSH to gain 340B eligibility, although this is a fairly recent phenomenon."<br><br>"Our findings support recent calls from the Government Accountability Office to improve oversight of the 340B program." | 1) interpretability of results: "only applies to hospitals "near" the eligibility threshold<br>2) no statistical tests available to check for the possibility of "hospitals adjust(ing) DSH well beyond the threshold"<br>3) "costs and benefits tied to 340B participation by hospitals may have shifted over time" |

© 2023 Knox RP et al. *JAMA Health Forum.*

| Article Citation | Article Type | Study Objective/Article Thesis | Stakeholders Discussed | Results/Analyses | Conclusions/ Recommendations | Limitations |
|---|---|---|---|---|---|---|
| Nikpay S, Buntin M, Conti RM. Diversity of Participants in the 340B Drug Pricing Program for US Hospitals. *JAMA Intern Med*. 2018;178(8):1124-1127. doi:10.1001/jamainternmed.2018.2015 | Research Letter | The article "examined how uncompensated care, provision of low-profit services, and financial stability differed between nonprofit and public hospital 340B participants and nonparticipants in 2015." | Hospitals | "Before 2004 (early participants), fewer than 200 hospitals participated in the 340B program. From January 1, 2004, through December 31, 2010 (intermediate participants), participation increased to 927 hospitals, predominantly among hospitals not targeted by expansions. After 2010 (late participants), participation reached 1046 hospitals, or 41.8% of all 2504 nonprofit and public general acute care hospitals in 2015… Early participants were larger (439 vs 338 beds), disproportionately public (81 [51.4%] vs 55 [11.0%]), academic (108 [67.9%] vs 91[18.2%]), and located in counties with lower income levels (18%vs 15%) and higher levels of uninsured patients (14% vs 12%) when compared with intermediate and late participants (P< .001)." | "As of 2015, 41.8% of all nonprofit and public general acute-care hospitals participated in the 340B program. Although participating hospitals provided more uncompensated care and low-profit services to patients despite worse finances than nonparticipants, later participants—most hospitals—spent less of their budget on uncompensated care and were more financially stable compared with earlier participants. Our results should be interpreted as descriptive owing to unmeasured confounding, and some of our outcome measures may be reported with error. Recent reimbursement reforms will likely have different effects across 340B participants. Targeting cuts might mitigate potential adverse effects on participants that provide a large amount of charitable medical care and operate at a substantial loss." | N/A |

© 2023 Knox RP et al. *JAMA Health Forum.*

| Article Citation | Article Type | Study Objective/Article Thesis | Stakeholders Discussed | Results/Analyses | Conclusions/ Recommendations | Limitations |
|---|---|---|---|---|---|---|
| Nikpay SS, Buntin MB, Conti RM. Relationship between initiation of 340B participation and hospital safety-net engagement. *Health Serv Res*. 2020;55(2):157-169. doi:10.1111/1475-6773.13278 | Original Research | The article "Examined whether 340B participation increases safety-net engagement." | Hospitals | 1) "New 340B participation was not associated with a change in uncompensated care, but was associated with a 28.9 percent increase in charity care spending (SE = 8.8), or about $880,000 per hospital. However, total community benefit spending (including charity care) did not change."<br>2) "340B was associated with an increase in the probability of offering discounted care (4.3 percentage points, SE = 1.6) from 84 to 88 percent and an increase in the income eligibility limit for discounted care (18.9 percentage points, SE = 5.6) from 294 to 313 percent."<br>3) "(340B) Participation was not associated with the probability of offering low-profit medical care services." | 1) "There is no association between uncompensated care, the standard measure of safety-net care provision, and 340B participation."<br>2) " Initiating participation in 340B is associated with an increase in some community benefit spending but reductions in other types of community benefit spending."<br>3) The increase in charity may not represent a true increase in safety-net care because: 1) the "percent increase in charity care identified in this paper translates to a miniscule increase in charity care" and 2) "the increase in charity care appeared to be offset by reductions in other types of community benefit spending"<br>4) The results adds support to recent evidence that "hospitals may manipulate the DSH patient percentage in order to qualify for 340B"<br>5) "Alternative measures show that newly participating hospitals may increase charity care, potentially through offering more patients discounted care. However, increases appear to be fully offset by reductions in other community benefit programs." | 1) the data "could not capture all the efforts hospitals may take to contribute to safety-net care", the SES status of the patients used them<br>2) the results "may not be generalized to the 198 hospitals that began participating before 2004" since they might "behave differently than hospitals that participated after 2004"<br>3) the community benefit spending data was reported at the hospital tax filing unit level<br>4) the study "considered all new participants as equally affected by the program but it is likely that some hospitals generate more revenue from the program than others"<br>5) since the study period was short, the study was unable to examine any associations with long-term outcomes.<br>6) "unable to consider the association of 340B with safety-net engagement for non-DSH hospitals."<br>7) "cannot fully address the possibility that hospitals raise charges after becoming eligible for 340B, thus inflating the value of hospital charity care"<br>8) no causal conclusion |

© 2023 Knox RP et al. *JAMA Health Forum.*

| Article Citation | Article Type | Study Objective/Article Thesis | Stakeholders Discussed | Results/Analyses | Conclusions/ Recommendations | Limitations |
|---|---|---|---|---|---|---|
| Nikpay S, Gracia G, Geressu H, Conti R. Association of 340B contract pharmacy growth with county-level characteristics. Am J Manag Care. 2022 Mar;28(3):133-136. doi: 10.37765/ajmc.2022.88840. PMID: 35404549. | Original Research | "To estimate the association of 340B contract pharmacy growth between 2009 and 2019 with county-level characteristics, including availability of health care providers, health care spending, population, and socioeconomic characteristics." | Hospitals, clinics, pharmacies | "We find that growth of contracts with 340B hospitals was uncorrelated with uninsured rates, poverty rates, or areas of medical underservice. By contrast, we find that growth of contracts with 340B safety-net clinics was positively correlated with poverty rates and metropolitan statistical status. These findings suggest different patterns of access for patients." | "Our results add systematic evidence of a difference in how the 2 main types of 340B participants-hospitals and safety-net clinics-use the 340B program. Policy proposals to reform 340B should consider reforms for safety-net clinics and hospitals separately." | 1) unable to assess volume of 340B-eligible prescriptions<br>2) county may not be a good proxy for retail pharmacy markets<br>3) Lacked unique pharmacy identifier |
| Radford A, Slifkin R, Schur C, Cheung K, Baernholdt M. Rural hospitals: are you missing out on drug savings?. *Healthc Financ Manage*. 2008;62(6):82-85. | Commentary | To describe how rural hospitals can benefit from the 340B program. | Hospitals | "For many rural hospitals, the financial benefits of participating in the 340B program outweigh the challenges of implementation and administration. Hospital administrators should take the time to learn whether their organizations are eligible. If not, they may be overlooking the potential for real cost savings."" | N/A | N/A |

© 2023 Knox RP et al. *JAMA Health Forum.*

| Article Citation | Article Type | Study Objective/Article Thesis | Stakeholders Discussed | Results/Analyses | Conclusions/ Recommendations | Limitations |
|---|---|---|---|---|---|---|
| Rana I, von Oehsen W, Nabulsi NA, et al. A comparison of medication access services at 340B and non-340B hospitals. *Res Social Adm Pharm*. 2021;17(11):1887-1892. doi:10.1016/j.sapharm.2021.03.010 | Original Research | To "Characterize medication access services provided at hospitals that participate in the 340B Drug Pricing Program compared to hospitals that do not participate in the 340B Program." | Hospitals | "340B hospitals provided a significantly higher average number of medication access services compared to non-340B hospitals (6.20 vs. 3.91, p = 0.0001), adjusted for differences in hospital size and ownership type. For all nine medication access services that were assessed, a higher percentage of 340B hospitals reported providing the service compared to non-340B hospitals."<br><br>"There was a significant difference between 340B and non-340B hospitals for the provision of drug/alcohol outpatient treatment services (37.5% vs. 9.5%) and HIV/AIDS outpatient services (39.3% vs. 9.5%). There was also a significant difference in the average number of general health care access services offered between 340B hospitals and non-340B hospitals, adjusted for differences in hospital size and ownership type (1.39 vs. 0.71, p = 0.0201)." | "340B hospitals provided more medication access services, on average, than comparably sized non- 340B hospitals, suggesting that hospitals participating in the 340B Drug Pricing Program may be better positioned to create and administer programs that support medication access services." | 1) majority of survey respondents were in the Midwest<br>2 Since 340B participation was assessed at the end of the questionnaire, it is unlikely that 340B participation played a role in propensity to submit a response. It is more likely that hospitals with more medication access services would be more eager to respond, agnostic to 340B status. As such, asymmetry in responses from 340B and non-340B hospitals may be more reflective of what programs (these hospitals) offer, or lack thereof. "<br>3) "there is no way to independently validate responses for accuracy" |

© 2023 Knox RP et al. *JAMA Health Forum.*

| Article Citation | Article Type | Study Objective/Article Thesis | Stakeholders Discussed | Results/Analyses | Conclusions/ Recommendations | Limitations |
|---|---|---|---|---|---|---|
| Romero LM, Olaiya O, Hallum-Montes R, et al. Efforts to Increase Implementation of Evidence-Based Clinical Practices to Improve Adolescent-Friendly Reproductive Health Services. *J Adolesc Health*. 2017;60(3S):S30-S37. doi:10.1016/j.jadohealth.2016.07.017 | Original Research | To "Describe changes in implementation of evidence- based clinical practices among health center partners as part of a multicomponent, community-wide teen pregnancy prevention initiative; to better understand the barriers to and facilitators of implementation of the evidence-based clinical practices; and to describe the technical assistance and training provided to the health center partners and key lessons learned" | Clinics | "Across 48 health centers in the 10 communities, 52% reported an increase in the implementation of evidence-based clinical practices from 2012 to 2013, mostly in providing contraceptive access (23%) and offering Quick Start (19%). Among health centers that reported a decrease in implementation of evidence-based clinical practices (35%), most reported a decrease in having either hormonal contraception or intrauterine devices available at every visit (15%), having HIV rapid testing available (10%), or participating in the federal 340B Drug Discount Program (2%)." "Health systems and community-level factors like (...) support from health center leadership, communication between leadership and staff, and staff attitudes and beliefs were reported as factors that facilitated the implementation of new practices" | "To increase adolescent's use of quality, client-centered, affordable and confidential reproductive health services, improvement in the implementation of evidence-based clinical practices is needed. Efforts to identify barriers to and facilitators for implementation of evidence- based clinical practices can inform for health centers of opportunities to build their capacity to ensure that evidence-based clinical practices are being implemented." | The generalizability of the results is limited as "the sample of 48 health centers reporting data for this analysis is not representative of all health centers participating in the Community-Wide Initiatives" |

© 2023 Knox RP et al. *JAMA Health Forum.*

| Article Citation | Article Type | Study Objective/Article Thesis | Stakeholders Discussed | Results/Analyses | Conclusions/ Recommendations | Limitations |
|---|---|---|---|---|---|---|
| Ross SL, Li BD. Consequences of the 340B Drug Pricing Program. *N Engl J Med*. 2018;378(21):2053. doi:10.1056/NEJMc1802999 | Medical Journal Reply | To respond to Desai & McWilliams' article | Hospitals | The reply offered the following critiques:<br>1) Authors excluded one third of DSH from analysis<br>2) "the authors focus on investments in federally qualified health centers and reduced mortality; these factors do not fully reflect how hospitals use their savings to treat low-income patients and those from rural areas"<br>3) Authors did not take into account other market trends in hospital consolidation | N/A | N/A |
| Ruhr LR, Grossman J, Odendahl R, Eisenberg DL. Contraceptive utilization at publicly funded clinics before and after introduction of low-cost levonorgestrel intrauterine system: a retrospective case study analysis. Women Health. 2022 Jan;62(1):75-84. doi: 10.1080/03630242.2021.2019171. PMID: 35021955. | Original Research | To "understand how the availability and utilization of a low-cost levonorgestrel intrauterine system (hormonal IUD) impacts uptake relative to other contraceptive methods" and "to determine if the percentage of self-pay women who obtained a hormonal IUD changed over time." | Clinics, patients | "Among self-pay women, hormonal IUD uptake increased 3 percentage points ($p < .001$) after the introduction of low-cost hormonal IUD. Privately insured women saw a 7-percentage point increase in hormonal IUD uptake while women whose family income was greater than 200% of the federal poverty level (FPL) saw a 13.9 percentage point increase in hormonal IUD uptake." | "The introduction of a low-cost hormonal IUD was associated with more self-pay and low-income women accessing this method. Access to and utilization of low-cost hormonal IUDs are complicated by many factors including product availability and provider training. | N/A |

© 2023 Knox RP et al. *JAMA Health Forum.*

| Article Citation | Article Type | Study Objective/Article Thesis | Stakeholders Discussed | Results/Analyses | Conclusions/ Recommendations | Limitations |
|---|---|---|---|---|---|---|
| Ruley M, Belcher M, Sayre H, Coustasse A. The 340B Program, Contract Pharmacies, Hospitals, and Patients: An Evolving Relationship Impacting Health Care Delivery. *Health Care Manag (Frederick)*. 2019;38(4):311-321. doi:10.1097/HCM.0000000000000279 | Systematic Review | To "Evaluate the growth and effects of the federal 340B Drug Pricing Program implementation and to determine whether it has decreased hospital expenditures" | Hospitals, pharmacies, patients | More hospitals are participating in the 340B Program and discounts are growing. Audits have found high rates of non-compliance. Hospitals are contracting with pharmacies more commonly. | "The 340B program has helped to offset hospitals' expenditures, but the ongoing increase in patients' out-of-pocket costs would imply that hospitals have used 340B to benefit themselves rather than their low-income patients. Additional studies and/or a meta-analysis of this topic would be helpful in determining where the additional funding associated with the 340B program payments to hospitals is being allocated by those hospitals." | There was a small number of articles reviewed and there is no consistent data available regarding hospital participation rate and profit margins. |
| Shi L, Wharton MK, Monnette A. Ensuring access to prescription medications in the post-ACA healthcare access landscape: the essential role of FQHCs in the safety net for the underinsured. *Am J Manag Care*. 2018;24(5 Suppl):S67-S73. | Original Research | "Federally qualified health centers (FQHCs) are essential to underinsured populations in the safety net by offering them several means of access to reduced cost medications. This study employed a 2-pronged approach to evaluate FQHCs' role, estimating both the need for patient assistance and the impact of the safety net." | Clinics, patients | "There were 1337 FQHCs serving more than 2.5 million patients, nearly 29% of whom were uninsured. FQHCs utilized 2 programs to provide affordable, reduced-reduced cost prescriptions for patients without insurance: 1) the HRSA 340B Drug Pricing Program and 2) prescription assistance programs, which rely on pharmaceutical manufacturer donations of reduced-cost medications or coupons. Although these programs were effective at providing affordable prescriptions, program accessibility varied widely by state and FQHC resources." | "Despite changes in the healthcare access landscape due to the ACA, underinsured populations remain prevalent and the need for financial assistance with medications persists. FQHCs are uniquely situated to provide access to these essential services. Further, policy and funding efforts, such as expansion of 340B programs, could assist FQHCs in fulfilling the role of prescription safety-net providers." | 1) only 2 years pre-Medicaid expansion data available<br>2) assume price of prescriptions in 340B programs and PAPS found in literature review applied to all FQHCs |

© 2023 Knox RP et al. *JAMA Health Forum.*

| Article Citation | Article Type | Study Objective/Article Thesis | Stakeholders Discussed | Results/Analyses | Conclusions/ Recommendations | Limitations |
|---|---|---|---|---|---|---|
| Thomas S, Schulman K. The unintended consequences of the 340B safety-net drug discount program. *Health Serv Res*. 2020;55(2):153-156. doi:10.1111/1475-6773.13281 | Review | To describe the unintended consequences of the 340B program, focusing on entity participation and financial incentives. | Hospitals, clinics, manufacturers | "Overall, then literature now suggests that the 340B program has offered a major financial windfall to covered entities with little of the benefit targeted toward safety-net providers who care for low-income patients. Further, 340B has placed hospitals in a position of benefitting financially from increases in drug prices (the higher the price, the greater the financial benefit from the 340B discount, even under Medicare. To place this impact in perspective, 50 percent of the increase in the cost of care in the United States from 1996 to 2013 was due to price increases in service intensity (in part a reflection of the migration to the hospital outpatient setting). The evidence seems to suggest that the 340B program, coupled to health system pricing leverage in the commercial market, has played an important role in these findings." | "Narrowing the criteria for organizations to be designated as covered entities, requiring specific application of 340B profits to charity care, and limiting the mark-up on the sale of 340B products to private payers (or eliminating the 340B discount for privately insured patients) are all approaches that can all reduce the distortions of the 340B program. None are likely to be politically popular, and it will be important to ensure that the elimination of 340B does not result in a financial windfall for pharmaceutical manufacturers. Unfortunately, changes in the organization of care from community to hospital-based treatment may persist even with the most aggressive reforms to the 340B program." | N/A |
| Traynor K. Children's hospitals seek drug-cost savings through 340B participation. *Am J Health Syst Pharm*. 2010;67(5):339-340. doi:10.2146/news100017 | News | To discuss how children's hospitals, newly eligible to participate in 340B, are signing up to participate and may achieve cost savings | Hospitals | Pharmacists at children's hospitals are hoping to achieve big savings with the 340B Program. | N/A | N/A |

© 2023 Knox RP et al. *JAMA Health Forum.*

| Article Citation | Article Type | Study Objective/Article Thesis | Stakeholders Discussed | Results/Analyses | Conclusions/ Recommendations | Limitations |
|---|---|---|---|---|---|---|
| Traynor K. Medicaid reform causes major 340B program changes in Illinois. *Am J Health Syst Pharm*. 2012;69(17):1451-1452. doi:10.2146/news120062 | News | To describe new Illinois law mandating all eligible entities participate in the 340B Program. | Hospitals, clinics, pharmacies | The new law mandates 340B participation and may have unintended consequences, including high costs to implement and administer the program (software, time, etc) and may cut into entities profit margins, depending on whether they can carve-out Medicaid patients. | N/A | N/A |
| Traynor K. Senators acknowledge importance of 340B program. *Am J Health Syst Pharm*. 2018;75(13):925-926. doi:10.2146/news180040 | News | To report on Senators' comments after a HELP Committee hearing on 340B oversight. | Hospitals, government | Senators recognized the importance of the 340B Program to access to care and hospitals' bottom lines, but disagreed as to what the purpose of the program is/should be and if and how it should be reformed. | N/A | N/A |
| US Health Resources and Services Administration. 340B Drug Pricing Program Ceiling Price and Manufacturer Civil Monetary Penalties Regulation. *Fed Regist*. 2017;82(3):1210-1230. | Federal Register Final Notice | To notify manufacturers participating in the 340B Program of the calculation of 340B ceiling prices and the application of civil monetary penalties | Hospitals, clinics, manufacturers | The final rule presents the calculation for 340B ceiling prices (including when penny pricing applies) and sets forth the circumstances for non-compliance that can result in manufacturers receiving civil monetary penalties. | N/A | N/A |
| US Health Resources and Services Administration. Final notice regarding section 602 of the Veterans Health Care Act of 1992 duplicate discounts and rebates on drug purchases. *Fed Regist*. 1993;58(119):34058-34059. | Federal Register Final Notice | To announce the final rule to prevent duplicate discounts and rebates | Hospitals, clinics, state governments | HRSA will record and submit the Medicaid provider number of each covered entity to state Medicaid programs. | N/A | N/A |

© 2023 Knox RP et al. *JAMA Health Forum.*

| Article Citation | Article Type | Study Objective/Article Thesis | Stakeholders Discussed | Results/Analyses | Conclusions/ Recommendations | Limitations |
|---|---|---|---|---|---|---|
| US Health Resources and Services Administration. Notice regarding section 602 of the Veterans Health Care Act of 1992--rebate option. *Fed Regist*. 1998;63(124):35 239-35242. | Federal Register Final Notice | To inform interested parties on final guidance for covered entities | Hospitals, clinics | HRSA provided guidance on the confidentiality of 340B pricing, preventing duplicate discounts and diversion, eligibility for retroactive discounts, voluntariness of participation, audit requirements, eligible drugs, and working with group purchasing organizations, purchasing agents, and wholesalers. | N/A | N/A |
| US Public Health Service. Final notice regarding section 602 of the Veterans Health Care Act of 1992 entity guidelines. *Fed Regist*. 1994;59(92):25110-25114. | Federal Register Final Notice | To "inform interested parties of the final guidelines recognizing a rebate option for State AIDS Drug Assistance Programs (ADAPs) receiving funds under Title XXVI of the PHS Act as an optional alternate means of accessing section 340B discount pricing." | Clinics | "HRSA recognizes rebates obtained by the State ADAPs or their components that equal or exceed the 340B discount provided by the statutory ceiling price as a method of participating in the 340B program, subject to compliance with other requirements for participation. Standard business practices, such as those reflected in the Medicaid Rebate Program and current voluntary manufacturer rebate programs (consistent with the requirements of section 340B and all program guidance published in the Federal Register) are appropriate for the development of rebate contracts and agreements between State ADAPs and manufacturers."<br><br>Previous guidance for 340B covered entities apply to State ADAPs. | N/A | N/A |

© 2023 Knox RP et al. *JAMA Health Forum.*

| Article Citation | Article Type | Study Objective/Article Thesis | Stakeholders Discussed | Results/Analyses | Conclusions/ Recommendations | Limitations |
|---|---|---|---|---|---|---|
| Warren A, Shankar A. Oncology Transactions and the 340B Drug Pricing Program. *J Oncol Pract*. 2013;9(2):89-91. doi:10.1200/JOP.2013.000895 | Medical Journal | To provide "an overview of the 340B program and discuss[] various business and transaction models through which 340B drug pricing may be available." | Hospitals | Hospitals could expand their access to the 340B program by opening outpatient clinics, to enter into an affiliation with a medical oncology practice, entering into an affiliation with a noneligible hospital and establishing an oncology clinic; hospitals should be aware of the increased scrutiny of the 340B program by the government and pharma and the delays in being able to register new facilities in the 340B program. | Business models could expand 340B hospitals participation in the program, particularly in oncology. | N/A |
| Werling K, Abraham S, Strelec J. The 340B Drug Pricing Program: an opportunity for savings, if covered entities such as disproportionate share hospitals and federally qualified health centers know how to interpret the regulations. *Health Care Law Mon*. 2007;3-12. | Policy Analysis | To describe the opportunity for savings provided by the 340B Program and review the proposed rule on patient definition | Hospitals, clinics, manufacturers, patients | The proposed rule on patients required 1) the specific professional-facility relationships that satisfy the proposed patient definition and 2) the type of providers that can offer services that qualify an individual as a patient under the newly proposed patient definition, and the criteria that enable covered entity employees to be qualified as patients under the proposed guidelines. Manufacturers supported the changes while covered entities found them too restrictive. | "A Covered Entity can realize significant savings by participating in the 340B Drug Pricing program. Compliant participation requires an entity to correctly interpret numerous regulations, a process made even more difficult by proposed changes to Program definition." | N/A |

© 2023 Knox RP et al. *JAMA Health Forum.*

| Article Citation | Article Type | Study Objective/Article Thesis | Stakeholders Discussed | Results/Analyses | Conclusions/ Recommendations | Limitations |
|---|---|---|---|---|---|---|
| Werling K, Abraham S, Strelec J. The 340B Drug Pricing Program: an opportunity for savings, if covered entities such as disproportionate share hospitals and federally qualified health centers know how to interpret the regulations. *J Health Care Finance*. 2007;34(2):57-70. | Policy Analysis | To describe the opportunity for savings provided by the 340B Program and review the proposed rule on patient definition | Hospitals, clinics, manufacturers, patients | The proposed rule on patients required 1) the specific professional-facility relationships that satisfy the proposed patient definition and 2) the type of providers that can offer services that qualify an individual as a patient under the newly proposed patient definition, and the criteria that enable covered entity employees to be qualified as patients under the proposed guidelines. Manufacturers supported the changes while covered entities found them too restrictive. | "A Covered Entity can realize significant savings by participating in the 340B Drug Pricing program. Compliant participation requires an entity to correctly interpret numerous regulations, a process made even more difficult by proposed changes to Program definition." | N/A |

© 2023 Knox RP et al. *JAMA Health Forum.*

| Article Citation | Article Type | Study Objective/Article Thesis | Stakeholders Discussed | Results/Analyses | Conclusions/ Recommendations | Limitations |
|---|---|---|---|---|---|---|
| Whittington MD, Campbell JD, McQueen RB. Achieving high value care for all and the perverse incentives of 340B price agreements. *Neurol Clin Pract*. 2018;8(2):148-152. doi:10.1212/CPJ.0000000000000437 | Original Research | To understand how 340B impacts cost-effectiveness analyses | Manufacturers | "We found that the amount paid to the manufacturer (340B price) was a good value ($118,256 per quality-adjusted life-year); however, from the payer drug cost perspective, good value ($196,683 per quality-adjusted life year) was not achieved. Given that emerging value frameworks incorporate cost-effectiveness, these price variations may have downstream negative consequences, including inaccurate coverage and reimbursement policy recommendations. Upcoming policy changes to the 340B program should incentivize pricing schemes hinged on transparency and value." | "Agreements like 340B likely hinder the delivery of high value care due to the variation in drug price by sector. This multiple sclerosis case study provides one example of how the variation in drug prices from agreements like 340B could be large enough to provide different estimates of value. Given that some emerging US value assessment frameworks incorporate estimates of cost-effectiveness, these price variations may have downstream negative consequences, including inaccurate coverage and reimbursement policy recommendations. Future research should expand this investigation to other 340B-eligible pharmaceuticals. Upcoming policy changes to the 340B program should incentivize pricing schemes hinged on transparency and value to help achieve sustainable and high value health care for all." | Uses estimated 340B and negotiated discounts, not actual figures |

| Article Citation | Article Type | Study Objective/Article Thesis | Stakeholders Discussed | Results/Analyses | Conclusions/ Recommendations | Limitations |
|---|---|---|---|---|---|---|
| Wood WW, Weingart W. 340B drug discount program in the cross hairs. *Am J Pharm Benefits*. 2014;6(5):204-207. | News | To review criticisms of the 340B Program | Hospitals, clinics, manufacturers, pharmacies | Critics of the 340B Program have recommended the 340B Program be restricted to benefit uninsured patients. They have also criticized the growth of the program, the use of contract pharmacy arrangements, and the expansion into oncology and physician practices. | "The 340B drug discount program has become controversial. Drug industry and private oncology critics want to shrink the program by limiting hospital and patient eligibility. All stakeholders are awaiting regulatory guidance from the government, which has been held up because of legal wrangling. Safety net hospitals argue that 340B savings are essential to helping them meet their missions to treat the underserved." | |

| Article Citation | Article Type | Study Objective/Article Thesis | Stakeholders Discussed | Results/Analyses | Conclusions/ Recommendations | Limitations |
|---|---|---|---|---|---|---|
| Wu T, Williams C, Vranek K, Mattingly TJ 2nd. Using 340B drug discounts to provide a financially sustainable medication discharge service. *Res Social Adm Pharm*. 2019;15(1):114-116. doi:10.1016/j.sapharm.2018.03.065 | Original Research | To evaluate the sustainability of (a medication bedside delivery program) program with and without 340B discounts | Hospitals, pharmacies, patients | 1) "The profitability of the current bedside delivery service (utilizing 340B prices) demonstrated a net benefit when utilizing 340B pricing. (…) However the financial cost- benefit analysis utilizing non-340B prices demonstrated a negative financial benefit."<br>2) "Potential expansion of delivery services would operate at a loss if implemented in a regular retail acquisition cost scenario, but would be profitable in a 340B drug pricing scenario."<br>3) "Expansion of (bedside delivery) services throughout the hospital produces a positive earnings before interest, taxes, depreciation, and amortization, which could be reinvested in better patient care. However, without the 340B price discounts, this service would actually operate at a financial loss. This demonstrates the importance of participation in the 340B program for a large dis proportionate share hospital." | "Existing outpatient pharmacy bedside delivery services are financially beneficial to an institution participating in the 340B drug pricing program. Without the 340B drug price discounts, the service would operate at a financial loss, and further investigation must be done to determine whether other clinical or economic benefits would warrant medication delivery at the institution." | 1) "bedside delivery technicians and workflow at the present institution were not at maximum operational efficiency during the data collection period", which may lead to an overestimation of the cost<br>2) some unmeasured confounders may lead to higher cost estimates in the financial model<br>3) " the profit margins stated from the simulation may be underestimated as we continually see efficiency and workflow process improvement"<br>4) didn't include depreciation of assets in the model<br>5) didn't consider future technology upgrades or other investments as this is a short-term study<br>6) "(the) fixed costs included a laptop computer on a mobile workstation which may be more expensive compared to other technology options." |

© 2023 Knox RP et al. *JAMA Health Forum.*

| Article Citation | Article Type | Study Objective/Article Thesis | Stakeholders Discussed | Results/Analyses | Conclusions/ Recommendations | Limitations |
|---|---|---|---|---|---|---|
| Xiao R, Ross JS, Gross CP, Dusetzina SB, McWilliams JM, Sethi RKV, Rathi VK. Hospital-Administered Cancer Therapy Prices for Patients with Private Health Insurance. JAMA Intern Med. 2022 Jun 1;182(6):603-611. doi: 10.1001/jamainternmed.2022.1022. PMID: 35435948; PMCID: PMC9016607. | Original research | "To assess the extent of price markup by hospitals on parenterally administered cancer therapies and price variation among hospitals and between payers at each hospital." | Hospitals, patients | "Of 61 NCI-designated cancer centers, 27 (44.3%) disclosed private payer-specific prices for at least 1 top-selling cancer therapy as required by federal regulations. Median drug price markups across all centers and payers ranged between 118.4% (sipuleucel-T) and 633.6% (leuprolide). Across-center price ratios ranged between 2.2 (pertuzumab) and 15.8 (leuprolide). Negotiated prices also varied considerably between payers at the same center; median within-center price ratios for cancer therapies ranged from 1.8 (brentuximab) to 2.5 (bevacizumab)." | "Most NCI-designated cancer centers did not publicly disclose payer-specific prices for cancer therapies as required by federal regulation. The findings of this cross-sectional study suggest that, to reduce the financial burden of cancer treatment for patients, institution of public policies to discourage or prevent excessive hospital price markups on parenteral chemotherapeutics might be beneficial." | The findings may not be generalizable to non-oncology drugs. The study may have underestimated treatment prices and price variations. The study also could not estimate the proportion of patients covered by different insurance plans and the impact of variations in negotiated prices on patient out of pocket costs. |
| Yang YT, Chen B, Bennett CL. Federal 340B Program Payment Scheme for Drugs Designated As Orphan Products: Congressional Clarification Needed to Close the Government-Industry Revolving Door. *J Clin Oncol*. 2016;34(36):4320-4322. doi:10.1200/JCO.2016.68.2989 | Opinion | To describe the impact of the orphan drug exclusion in the 340B Program and related litigation | Hospitals, clinics, manufacturers | Because Congress did not delegate HRSA with authority, manufacturers are no longer required to offer 340B discounts on orphan drugs to certain hospitals. This also may initiate future lawsuits related to HRSA's guidance. | "Congress should consider amending the statute to clarify that the Orphan Exclusion is applicable only when these drugs are administered for rare indications. Congress should also provide HHS with additional rule-making authority to interpret the 340B statute and address other oversight issues that are the subject of continued debate between 340B covered entities and pharmaceutical manufacturers." | N/A |

© 2023 Knox RP et al. *JAMA Health Forum.*

| Article Citation | Article Type | Study Objective/Article Thesis | Stakeholders Discussed | Results/Analyses | Conclusions/ Recommendations | Limitations |
|---|---|---|---|---|---|---|
| Zeta LM. Comprehensive Legislative Reform to Protect the Integrity of the 340B Drug Discount Program. *Food Drug Law J*. 2015;70(4):481-499. | Law Journal | To discuss the limitations of HRSA's authority and to recommend reforms. | Hospitals, clinics, manufacturers | The prohibitions on duplicate discounting and diversion are a "legal fiction," limited by HRSA's lack of enforcement authority. Many covered entities are incentivized to use the 340B Program as a revenue stream. | The author recommends delegating adequate rulemaking authority to HRSA, defining what an eligible patient is for the 340B Program, and restructure the 340B Program as a rebate program as opposed to a discount program. | N/A |
| **EMBASE** | | | | | | |
| Burton-Meza C. (2016). Public Health Service Act, section 340B: 340B Drug Discount Program: Compliance risks associated with contract pharmacy arrangements. *J Health Care Finance*. 2016;42(4):2-21 | Commentary | To describe current issues in 340B compliance related to contract pharmacies | Hospitals, clinics, pharmacies, manufacturers | 1) "HRSA's (current) guidance on key program requirements often lacks the necessary level of specificity to provide clear direction, making it difficult for participants to self-police or monitor others 'compliance and raising concerns that the guidance may be interpreted in ways that are inconsistent with intent."<br>2) "operating the 340B in contract pharmacies creates more opportunities for drug diversion compared to in-house pharmacies (because) they are more likely to serve both patients of covered entities and other in the community"<br>3) three activities have been strictly prohibited by the 340B statue: duplication of discount, diversion, and the Group Purchasing Organization exclusion; however, "there is a question of whether HRSA has the authority to make rules under the 340B program" | "In this current economic environment, the notion of allowing the participants to self-police themselves has been less than effective; (...) if Congress intends to fix this program, they must grant authority to HRSA to make rules that all participants must follow and not interpretive guidance that will be left to the participants' discretion." | N/A |

© 2023 Knox RP et al. *JAMA Health Forum.*

| Article Citation | Article Type | Study Objective/Article Thesis | Stakeholders Discussed | Results/Analyses | Conclusions/ Recommendations | Limitations |
|---|---|---|---|---|---|---|
| Eggers GG, Mark SM, Weber RJ. Director's forum-340B drug pricing program: Interpreting regulations and exploring opportunities. *Hosp Pharm*. 2011;46(5):368-373. | Commentary | To describe the 340B program to advise pharmacy directors on how they could improve programs for uninsured and underinsured patients. | Hospitals, pharmacies | "The regulations surrounding eligibility, drug pricing, and pharmacy contracting present complexities that can make participating in 340B seem intimidating and unattractive to potentially eligible organizations. By gaining further understanding of the intricacies of the 340B Drug Pricing Program and performing an analysis of their own facility, pharmacists and other health care providers can utilize the program to maximize the benefit for both their patients and their organization." | N/A | N/A |

© 2023 Knox RP et al. *JAMA Health Forum.*

| Article Citation | Article Type | Study Objective/Article Thesis | Stakeholders Discussed | Results/Analyses | Conclusions/ Recommendations | Limitations |
|---|---|---|---|---|---|---|
| Fratto E. Bicillin Delivery: Reducing Syphilis and Meeting Patients Where They Are. *Sexually Transmitted Diseases*. 2022;49(10):S56-S57. | Original research | To examine the role of the 340B Program in addressing the resurgence of syphilis | Hospitals, patients | "By implementing 340B compliant policies and procedures, STD programs can leverage the 340B Drug Pricing Program to support their efforts in addressing the syphilis epidemic. NCSD will host a panel of STD program managers and 340B experts to discuss Bicillin Delivery as a means to ensure timely and appropriate treatment for syphilis cases and address skyrocketing syphilis rates. The panel will provide service-level insight and discuss their experience working with private providers, health care partners, and disease intervention specialists (DIS) to ensure those diagnosed with syphilis receive timely and appropriate treatment." | "Accessing 340B priced penicillin G benzathine yields a cost savings of 99.99% for STD programs. Implementing a Bicillin Delivery program also removes structural barriers to timely and appropriate syphilis treatment including healthcare costs, pharmacy stock-onhand limitations, and referral-related loss to follow-up." | N/A |
| Gellad WF, James AE. Discounted Drugs for Needy Patients and Hospitals - Understanding the 340B Debate. *N Engl J Med*. 2018;378(6):501-503. doi:10.1056/NEJMp1716139 | Commentary | To describe the controversy surrounding CMS' decision to decrease Medicare Part B reimbursement for 340B drugs | Hospitals, federal government | "Critics contend that hospitals have purchased community practices in part for this reason — to expand their footprint into wealthier neighborhoods to "profit" from the 340B program."<br>"The hospitals view any reduction to the program, or restrictions on how they can use the generated revenue, as a threat to their ability to care for vulnerable and uninsured patients." | "The program became so large and convoluted that it requires scaling back, inevitably causing pain for hospitals that have spent years relying on the revenue it brought in and building businesses around it."<br><br>"As the current debate about 340B plays out and the policy changes are enacted in 2018, stay tuned for the next act in this long-playing drama." | N/A |

© 2023 Knox RP et al. *JAMA Health Forum.*

| Article Citation | Article Type | Study Objective/Article Thesis | Stakeholders Discussed | Results/Analyses | Conclusions/ Recommendations | Limitations |
|---|---|---|---|---|---|---|
| Guadamuz J, Qato D. Availability of pharmacies participating in the 340B drug pricing program, 2016. *Value Health*. 2018;21:S97. | Medical Journal Abstract | “to estimate the availability of 340B pharmacies and to examine the community characteristics associated with 340B pharmacy availability." | Pharmacies | “In 2016, one in four community pharmacies in the US participated in the 340B program. While chain and independent pharmacies account for 58.7% of 340B pharmacies, only 14.4% of independent pharmacies participated in the program…. Communities with the highest uninsured rates have lower availability of 340B pharmacies than all other communities. … Community prevalence of no insurance is negatively associated with 340B pharmacy availability.” | "One in five communities in the US have a 340B pharmacy. Community availability of 340B pharmacies is more common in poor communities. Despite the program’s aim of improving access to healthcare and medications for uninsured patients, communities with higher proportion of uninsured residents are lacking 340B pharmacies." | N/A |
| Guadamuz, JS, Qato DM. Availability of pharmacies participating in the 340B Drug Pricing Program, 2016. *Pharmacoepidemiol Drug Saf*. 2018;27:429-430. | Medical Journal Abstract | "(1) To estimate the availability of 340B pharmacies and (2) to examine the community characteristics associated with 340B pharmacy availability." | Pharmacies | “In 2016, one in four community pharmacies in the US participated in the 340B program. While chain and independent pharmacies account for 58.7% of 340B pharmacies, only 14.4% of independent pharmacies participated in the program. … Communities with the highest uninsured rates have lower availability of 340B pharmacies than all other communities… Community prevalence of no insurance is negatively associated with 340B pharmacy availability.” | "One in five communities in the United States have a 340B pharmacy. Community availability of 340B pharmacies is more common in poor communities. Despite the program’s aim of improving access to healthcare and medications for uninsured patients, communities with higher proportion of uninsured residents are lacking 340B pharmacies." | N/A |

© 2023 Knox RP et al. *JAMA Health Forum.*

| Article Citation | Article Type | Study Objective/Article Thesis | Stakeholders Discussed | Results/Analyses | Conclusions/ Recommendations | Limitations |
|---|---|---|---|---|---|---|
| Hou J, Clark B, Chou C, Huang E, Conti R. Medication adherence among 340B patients with hypertension, hyperlipidemia, and diabetes. *J Manag Care Spec Pharm*. 2016;22:S43. | Medical Journal Abstract | "To determine whether patients with diabetes, hypertension or hyperlipidemia (all among the top five therapeutic classes for which 340B prescriptions are dispensed), who receive medications for these diseases through a 340B program have higher medication adherence rates than a comparable patient population which does not receive medications through the 340B program." | Hospitals, clinics, pharmacies, patients | "Among the patients who received medications through a 340B program and whose prescriptions for such medications originated from 340B clinics … mean medication adherence was 5% higher for patients with diabetes, 3.4% higher for patients with hyperlidemia, and 2.9% higher for patients with hypertension than the respective comparison cohort. Patients who received medications through a 340B program and whose prescriptions for such medications originated from 340B hospitals … showed greater advantages in medication adherence relative to the general patient population. Mean medication adherence was 7.2% higher for patients with diabetes, 6.0% higher for patients with hyperlidemia, and 5.0% higher for patients with hypertension." | "The results from this study show that patients who receive medications through a 340B program have higher medication adherence rates than comparable patients who do not receive medications through the program." | N/A |

© 2023 Knox RP et al. *JAMA Health Forum.*

| Article Citation | Article Type | Study Objective/Article Thesis | Stakeholders Discussed | Results/Analyses | Conclusions/ Recommendations | Limitations |
|---|---|---|---|---|---|---|
| Hussaini SMQ, Johnson J, Chino F. Price variability of pembrolizumab across U.S. National Cancer Institute-designated Cancer Centers (NCICCs) from 2016-2021. *J Clin Oncology*. 2022;40(28):48. | Medical Journal Abstract | "We investigated variability in the charged and reimbursed payments for pembrolizumab utilizing Medicare claims across all NCICCs" | Hospitals, clinics | "Our analysis included 53 NCICCs of which 47 were part of the 340B Drug Pricing Program (NCI-340B), 11 were Prospective Payment System-exempt (NCI-PPS), and 6 NCICCs were both (PPS/340B). From 2016 to 2021, total Medicare claims increased from 9461 to 46291 across NCICCs, with NCI-PPS centers comprising 30% of claims. Total payments to NCICCs increased from \$55.9M to \$388.1M annually. Average charge for pembrolizumab in 2021 were: \$45,227 (NCI-PPS), \$46,000 (all NCICCs), \$49,057 (NCI-340B), \$59,392 (dual-eligible PPS/340B)…. When considering a smaller cohort of PPS-only or 340B-only, PPS-only status was associated with lowest charged price but highest reimbursement (33.5%, or \$8568)." | "NCICCs charged > 4 times the average reimbursed price for pembrolizumab, with charges increasing faster than inflation. While lower charges were noted at NCI-PPS, they received higher reimbursement. Dual PPS/340B status commanded the highest charged prices and quickest price increases. Our study informs greater price transparency regulation at NCICCs and raises questions regarding utility of special status benefits from the federal government." | N/A |
| Lasser KE, Heinz A, Battisti L, et al. A Hepatitis C Treatment Program Based in a Safety-Net Hospital Patient-Centered Medical Home. *Ann Fam Med*. 2017;15(3):258-261. doi:10.1370/afm.2069 | Original research | "This article describes an innovative and successful HCV primary care treatment program in a patient-centered medical home based at an urban, safety-net hospital." | Hospitals, patients | Of the 302 patients who were referred to the program, "all 46 patients who attended a visit 3 months after completing treatment achieved a sustained virologic response." | "Given the opportunity to design an HCV treatment program within primary care, this model, embedded in an urban safety-net hospital PCMH, achieved impressive outcomes and has the potential for dissemination." | Only one hospital was studies, so there is limited generalizability. |

© 2023 Knox RP et al. *JAMA Health Forum.*

| Article Citation | Article Type | Study Objective/Article Thesis | Stakeholders Discussed | Results/Analyses | Conclusions/ Recommendations | Limitations |
|---|---|---|---|---|---|---|
| Latimer H, Tomicki S, Dieguez G, Cockrum P, Kim GP. Dispersion in total cost of care for Medicare fee-for-service (FFS) patients with metastatic pancreatic cancer receiving FDA approved/NCCN Category 1 regimens at 340B versus non-340B institutions. *J Clin Oncol*. 2021;39(15 Suppl). DOI: 10.1200/JCO.2021.39.15_suppl.e18843 | Original Research | To "Analyze the dispersion in total cost of care (TCOC) for Medicare FFS patients (pts) with metastatic pancreatic cancer (m-PANC) treated at 340B or non-340B institutions, by NCCN Category 1 regimen" | Hospitals, patients | 1) "We identified 2,697 (340B) and 3,839 (non-340B) patients taking NCCN Category 1 regimens. Gem-mono represented 1% and 4% of all pts in 340B and non-340B institutions, respectively. Gem-nab accounted for 72% of pts in both cohorts."<br>2) "For gem-nab, FFX, and nal-IRI pts, median total cost of care was similar in both cohorts, although mean total cost of care by quartile was lower at 340B institutions than non-340B institutions, except for gem-nab in the 1st quartile.<br>3) "The components of total cost of care were similar between 340B and non- 340B institutions in all quartiles. In both cohorts, % inpatient care costs increased between the 1st and 4th qrt (340B:15% to 23%, non-340B:14% to 25%). From the 1st to the 4thqrt, mean rates of admissions increased in both cohorts." | "Median total cost of care was lower at 340B institutions than non-340B institutions for all regimens, and the range of total cost of care dispersion was also smaller at 340B institutions. Across quartiles, chemotherapy accounted for approximately half the total cost of care; however, inpatient care costs were proportionally higher in the 4th quartile. Comparing regimens, despite 2L nal-IRI patients being more heavily pretreated, median costs in each cohort were similar to 1L gem-nab and 1L FFX, while mean rates of admission were generally lower than 1L gem-nab and 1L FFX across quartiles and cohorts." | N/A |

© 2023 Knox RP et al. *JAMA Health Forum.*

| Article Citation | Article Type | Study Objective/Article Thesis | Stakeholders Discussed | Results/Analyses | Conclusions/ Recommendations | Limitations |
|---|---|---|---|---|---|---|
| Lee C, McCombs J, Stone B, Chang J. Evaluation of drug price trending in the federal 340b drug discount program. *Value Health*. 2017;20(5):A126. | Medical Journal Abstract | "The Federal 340B drug discount program provides access to significant drug price discounts for healthcare organizations serving disadvantaged patients. Currently there are no published studies documenting pricing trends in the 340B program. In this project, we analyzed drug price trends in the 340B program over a 10-year period." | Hospitals, clinics, pharmacies | "340B prices declined relative to AWP over time across all drug classes. Overall drug price growth rate over 10-years was 16% for AWP and 19% for 340B (p = 0.88). The growth rate variations were similar after 2010. Among high cost drug classes, the 10-year price growth rates were: 11% in AWP and 5% in 340B in antiretroviral drugs (p < 0.01), 58% in AWP and 32% in 340B in antineoplastic drugs (p = 0.37), 16% in AWP and -6% in 340B in disease-modifying antirheumatic drugs (DMARD) (p=0.07) and 14% in AWP and 15% in 340B in antidiabetic drugs (p=0.97). For specialty drug classes, such as antineoplastic drugs, antiretroviral drugs and DMARDs, the 340B price growth rates were smaller than AWP growth rates after 2014." | "The relatively low drug price in the 340B program provides significant financial savings for eligible healthcare organizations. Eligible organizations with high specialty drug volume would benefit the most from the 340B program." | N/A |

© 2023 Knox RP et al. *JAMA Health Forum.*

| Article Citation | Article Type | Study Objective/Article Thesis | Stakeholders Discussed | Results/Analyses | Conclusions/ Recommendations | Limitations |
|---|---|---|---|---|---|---|
| Zappa, A. Proposed rule changes for 340B programs: Overview and impact. *Am J Pharm Benefits*. 2015;7(5):233-237. | Review | To review and assess the proposed rule changes governing 340B programs | Hospitals, clinics, manufacturers, pharmacies | The proposed rule would exclude referral and discharge prescriptions from the 340B Program, remove Medicaid managed care organizations from contract pharmacy programs, and require covered entities conduct quarterly reviews of contract pharmacies. | "There are several material changes to the 340B program in this proposed guidance. The most material changes are removal of discharge prescriptions, removal of referral providers, removal of Medicaid MCO prescriptions in contract pharmacy programs, and the quarterly contract pharmacy reviews. Together, these could remove upwards of 30% to 40% of currently included prescriptions and significantly increase administrative oversight. These changes may cause some entities with small programs to voluntarily stop their programs and move back to using GPO pricing." | N/A |

© 2023 Knox RP et al. *JAMA Health Forum.*

| Article Citation | Article Type | Study Objective/Article Thesis | Stakeholders Discussed | Results/Analyses | Conclusions/ Recommendations | Limitations |
|---|---|---|---|---|---|---|
| **National Bureau of Economic Research** | | | | | | |
| Howard DH Back PB, Berndt ER, Conti RM. Pricing in the Market for Anticancer Drugs. https://www.nber.org/papers/w20867. Published January 2015. Accessed September 22, 2021. | Working Paper | To study the change in anticancer drug launch prices from 1995 to 2013. | Manufacturers | In addition to broadened eligibility for 340B entities, "mergers between 340B providers and non-340B providers (...) have also expanded the program's reach."<br><br>1) "Recent increases in the launch prices of anticancer drugs may be an unintended consequence of policies to expand access to price discounts."<br>2) "Because the 340B discount is based on a drug's average price, the program presents manufacturers with an incentive to set higher launch prices to offset discounts. Increases in the number of 340B-eliglble providers have magnified the incentive, possibly leading to upward pressure in the prices paid by non-eligible providers."<br>3) "The 340B program also splits the market into price-elastic and price-inelastic segments. Just as branded drug manufacturers increase prices following generic entry to capture revenues from brand-loyal customers, manufacturers of recently launched drugs may cede large discounts to their price-sensitive segment but increase prices to non-340B providers." | "Manufacturers may set higher launch prices over time as standards evolve. Pricing trends may also reflect manufacturers' response to expansions in the 340B Drug Pricing Program, which requires manufacturers to provide steep discounts to eligible providers." | N/A |

© 2023 Knox RP et al. *JAMA Health Forum.*

| Article Citation | Article Type | Study Objective/Article Thesis | Stakeholders Discussed | Results/Analyses | Conclusions/ Recommendations | Limitations |
|---|---|---|---|---|---|---|
| **EconLit** | | | | | | |
| Dietrich M, Gregory D. Chapter 11: Section 340B expansion. In Dietrich M, Gregory D. *The Financial Professional's Guide to Healthcare Reform*. 1st ed. Wiley. 2012;361-272. | Book | To provide an overview of the 340B Program (chapter within) | Hospitals, clinics, manufacturers | The chapter provides an overview of the changes the Affordable Care Act made to the 340B Program and reviews the proposed civil monetary penalty, administrative dispute resolution, and orphan drug rules. | N/A | N/A |

© 2023 Knox RP et al. *JAMA Health Forum.*

| Article Citation | Article Type | Study Objective/Article Thesis | Stakeholders Discussed | Results/Analyses | Conclusions/ Recommendations | Limitations |
|---|---|---|---|---|---|---|
| Nikpay S. The Medicaid windfall: Medicaid expansions and the target efficiency of hospital safety-net subsidies. *J Public Econ*. 2022:208; 104583. | Original Research | This paper presents the first evidence on target efficiency of Medicaid-based eligibility criteria for hospital safety-net subsidies. | Hospitals, clinics, patients | 1) "The target efficiency of Medicare DSH, Medicaid DSH, and 340B ranged from 43% to 53%. These target efficiency estimates suggest that approximately half of hospitals are either inappropriately included in or excluded from safety-net subsidy programs when using the 5% uncompensated care requirement to define safety-net hospitals."<br>2) "estimates from two-way fixed effects event study models… show that the ACA Medicaid expansion increased receipt of Medicare DSH and 340B safety-net subsidies by 9% and 31% respectively, but reduced the amount of uncompensated care hospitals provided by 47%.<br>3) The disenrollment resulted in an 8% reduction in the DSH patient percentage and a corresponding 4% reduction in Medicare DSH receipt, and 56% reduction in 340B participation, despite the fact that uncollectible patient bills increased in Tennessee relative to surrounding states. Target efficiency increased as a result of these changes by 15%." | "I show that subsidies to support hospitals that contribute significantly to the US health care safety-net are poorly targeted, and that Medicaid expansions actually worsen targeting over time. Current policy proposals to reform the safety-net are insufficient to improve targeting, and eligibility criteria must be revised directly. In the presence of scarce federal resources, continuing to poorly target subsidies to hospitals with significant uncompensated care burdens could ultimately weaken the safety-net." | 1) study relies on hospital administrative data<br>2) Used CBO safety-net criteria<br>3) Adapted 1950s era non-profit status criteria as a measure of safety-net status<br>4) The "conceptual model suggests that target efficiency can be improved by revising eligibility criteria. However, the extent to which Medicaid expands depends upon state decisions. Therefore, the resulting revisions to eligibility criteria would need to be state-specific rather than one national-level revision as I have proposed." |

© 2023 Knox RP et al. *JAMA Health Forum.*

| Article Citation | Article Type | Study Objective/Article Thesis | Stakeholders Discussed | Results/Analyses | Conclusions/ Recommendations | Limitations |
|---|---|---|---|---|---|---|
| **Westlaw – Secondary Sources** | | | | | | |
| Applegate D, Gollwitzer A. Impediments to innovation: Implications for national health care legislation for the intellectual property community. *Engage: J Federalist Soc Pract Groups*. 2010;11:64-67. | Law Journal | To describe the potential impact of the ACA on innovation and intellectual property rights. | Hospitals, clinics, manufacturers | "The GAO report must address whether the 340B program "should be expanded since it is anticipated that the 47,000,000 individuals who are uninsured as of the date of enactment of this Act will have health care coverage once this Act is implemented." 14 Any expansion of the 340B Program--that is, expanding either the number of entities allowed to purchase discount drugs (or the number of drugs available at discount prices), or decreasing the discount price for available drugs--will necessarily reduce payments to drug makers." | ""At bottom, the "Patient Protection and Affordable Care Act" both explicitly and implicitly impacts innovation and intellectual property rights, in some ways that are difficult or impossible to predict. Directly, the Act imposes a complicated regulatory scheme, details yet to be provided, concerning intellectual property protection of biologic drugs. Indirectly, the Act takes resources of drug companies and medical device makers and importers that otherwise might fund research and development or directly lower product costs. It remains to be seen, in other words, whether the recently-passed national health care legislation will violate the first and oldest rule in medicine: first, do no harm." | N/A |

© 2023 Knox RP et al. *JAMA Health Forum.*

| Article Citation | Article Type | Study Objective/Article Thesis | Stakeholders Discussed | Results/Analyses | Conclusions/ Recommendations | Limitations |
|---|---|---|---|---|---|---|
| Baer CJ. Drugs for the indigent: A proposal to revise the 340B Drug Pricing Program. *William & Mary Law Rev*. 2015;57:637-673. | Law Journal | To provide a comprehensive plan to reform the 340B Drug Pricing Program | Hospitals, clinics, manufacturers, pharmacies, patients | Criticisms of the 340B Program include the definition of patient, hospital profits, and the limited authority of HRSA. The author argues the three-part proposal would pass on greater discounts to patients while maintaining incentives for manufacturers and covered entities. However, the proposal could have higher administrative costs. | Reform to the 340B Program is needed. "First, Congress should untether 340B from the Medicaid drug rebate program, allowing 340B to target only the specific populations within its scope and thus more appropriately serve its intended beneficiaries. Second, Congress should recognize the inherent differences among the patient populations currently served by 340B and tailor drug discounts to match each group's ability to pay. Third, Congress needs to authorize HHS with the appropriate mandate to administer the 340B program effectively." | N/A |

© 2023 Knox RP et al. *JAMA Health Forum.*

| Article Citation | Article Type | Study Objective/Article Thesis | Stakeholders Discussed | Results/Analyses | Conclusions/ Recommendations | Limitations |
|---|---|---|---|---|---|---|
| Ballreich J, Levengood T, Conti RM. Opportunities and Challenges of Generic Pre-Exposure Prophylaxis Drugs for HIV. J Law Med Ethics. 2022;50(S1):32-39. doi: 10.1017/jme.2022.33. PMID: 35902088; PMCID: PMC9341204. | Law Journal | "In this study, we examine early utilization patterns and prices for generic PrEP. We discuss the opportunities and challenges for generic PrEP to improve health among HIV vulnerable populations." | Hospitals, clinics, patients | Generic PrEP would reduce the price of the drug and improve access. However, the 340B Program is an impediment for generic drug uptake, including in the context of Descovy (despite little evidence of superior benefit to Truvada). These financial benefits are less apparent for FQHCs, who are statutorily required to provide care on a sliding scale based on ability to pay. | "In this analysis, the introduction of generic PrEP significantly reduced the per-unit price of the drug. Lower priced generic PrEP should result in increased overall uptake of PrEP; however, our analysis of early utilization patterns of generic PrEP suggests generic PrEP may be cannibalizing branded PrEP. While generic PrEP holds promise to reduce cost-related access barriers and help the U.S. reach the goal of 50% uptake of PrEP in the US by 2030, structural challenges in the PrEP market such as the 340B provider incentives may hinder increased PrEP uptake." | N/A |

© 2023 Knox RP et al. *JAMA Health Forum.*

| Article Citation | Article Type | Study Objective/Article Thesis | Stakeholders Discussed | Results/Analyses | Conclusions/ Recommendations | Limitations |
|---|---|---|---|---|---|---|
| Barker TR. A critical analysis of provisions of the Affordable Care Act affecting the life sciences industry. *J Health Biomed Law*. 2011;7:67-90. | Law Journal | To describe how the Affordable Care Act affected the life sciences industry, including how it "changes the manner in which pharmaceutical products are covered by Medicaid and the Public Health Service Act section 340B program" | Manufacturers | "Nonetheless, one particular concern of manufacturers is that the legislation left unaddressed the fact that there is no prohibition on the entity's ability to re-sell the drug to patients at a higher rate; whereas a covered entity must be able to purchase a drug at a favored price. For the first time, however, requirements for compliance, coupled with the possibility of civil monetary penalties for noncompliance, were included into the statute… Although this expansion is potentially negative for manufacturers, on the positive side, the new law creates more compliance requirements on covered entities. In addition, it creates some new markets for manufacturers." | "The Affordable Care Act, most notable in the popular press for the Act's re-structuring of the American health care system, contains provisions of significant interest to the life sciences industry. These include the new assessment on pharmaceutical manufacturers and medical device manufacturers, revisions to the Medicaid prescription drug rebate program, the expansion of the Public Health Service Act section 340B program, and adjustments to the Medicare Part D prescription drug benefit. These changes create both challenges and strategic business opportunities for pharmaceutical manufacturers." | N/A |

© 2023 Knox RP et al. *JAMA Health Forum.*

| Article Citation | Article Type | Study Objective/Article Thesis | Stakeholders Discussed | Results/Analyses | Conclusions/ Recommendations | Limitations |
|---|---|---|---|---|---|---|
| Bobroff R. Medicaid preemption claims in *Douglas*: Avert the Astra Abyss. *Yale Law J Online*. 2012;122:19-23. | Law Review | To review the challenges of bringing Medicaid preemption claims after *Douglas* and *Astra* | Hospitals, clinics, manufacturers, patients | *Astra* may pose a challenge to future suits enforcing obligations under the Medicaid statute due to its interpretation of third party beneficiary contracts and the lack of a private right of action in the statute. | "Looking to the future, the Patient Protection and Affordable Care Act expands Medicaid eligibility in 2014, increasing the number of people who will rely upon Medicaid's provisions. As Medicaid becomes a more substantial player in the United States health-insurance system, the statute's enforceability assumes greater importance. The Douglas decision does not prevent court access to enforce Medicaid, but the threat of the dissent's interpretation of Astra still looms." | N/A |
| Church RP, Hamscho VK. Contract pharmacy restrictions, legal challenges, and congressional action: What to expect from the 340B Drug Pricing Program. *J Health Care Compliance*. 2021;23(1):45-77. | Legal Publication | To provide an update on 340B contract pharmacy restrictions and related litigation | Hospitals, clinics, manufacturers, pharmacies | HRSA has advised that the 2010 guidance document is its interpretation of contract pharmacy requirements. Congress has urged for HHS to intervene. | "Absent administrative, judicial, or congressional action, it is likely that more drug manufacturers will restrict contract pharmacy access to 340B pricing and request claims data from covered entities… any legislative fix should be closely watched by covered entities and will likely come with new compliance requirements for covered entities as well as manufacturers in the years ahead." | N/A |

© 2023 Knox RP et al. *JAMA Health Forum.*

| Article Citation | Article Type | Study Objective/Article Thesis | Stakeholders Discussed | Results/Analyses | Conclusions/ Recommendations | Limitations |
|---|---|---|---|---|---|---|
| Comer C, Fernández R. Health Departments and PrEP: A Missed Opportunity for Public Health. *J Law Med Ethics*. 2022;50(S1):64-68. doi: 10.1017/jme.2022.39. PMID: 35902080; PMCID: PMC9341197. | Law Journal | "The paper identifies common barriers and challenges to Pre-Exposure Prophylaxis (PrEP) uptake and offers considerations for state and local public health departments to address barriers and retool infrastructure to increase access to PrEP to new users." | Hospitals, clinics, state governments | Various programs keep PrEP unaffordable and prevent patients from using PrEP. One such program is the 340B Program, which incentivizes providers to prescribe higher cost medications in lieu of available cheaper, equally effective brand or generic drugs. | "While there is variability in different jurisdictions, health departments have some power to effectuate change in their communities to increase utilization of PrEP. It is crucial that health departments do what they can to increase the uptake of PrEP. A new purchasing and distribution model could absorb financial and administrative burden so that key populations can be served and meaningful work can be done within communities and health systems to achieve the goals of ending the HIV epidemic." | N/A |

© 2023 Knox RP et al. *JAMA Health Forum.*

| Article Citation | Article Type | Study Objective/Article Thesis | Stakeholders Discussed | Results/Analyses | Conclusions/ Recommendations | Limitations |
|---|---|---|---|---|---|---|
| Craig RC. Supreme Court news. *Administrative Regul Law News*. 2011;36:20-26. | Law Review | To summarize the Supreme Court's opinion in *Astra v. Santa Clara County* | Hospitals, clinics, manufacturers | "The Supreme Court unanimously … denied medical facilities a cause of action against pharmaceutical manufacturers as third-party beneficiaries of pharmaceutical pricing agreements between the federal government and those manufacturers for Medicaid programs, emphasizing interference with the administrative scheme of enforcement. In an opinion by Justice Ginsburg, the Court held that such a private right of action by "Section 340B" facilities against drug manufacturers who charged more than the statutory ceiling allowed under the Public Health Services Act (PHSA), 42 U.S.C. § 256b, would be "incompatible with the statutory regime" administered by the Department of Health and Human Services (HHS)." | N/A | N/A |
| Curran AV, Schott CH, Peterson KA, et al. 340B Program: HRSA issues dispute resolution proposed rule. *J Health Care Compliance*. 2016;18(5):45-56. | Legal journal | To describe the proposed administrative dispute resolution process rule | Hospitals, clinics, manufacturers, HRSA | The article outlines the administrative dispute resolution process as set forth in the Notice of Proposed Rulemaking. | N/A | N/A |

© 2023 Knox RP et al. *JAMA Health Forum.*

| Article Citation | Article Type | Study Objective/Article Thesis | Stakeholders Discussed | Results/Analyses | Conclusions/ Recommendations | Limitations |
|---|---|---|---|---|---|---|
| Finegan S, Keller S, O'Neill S, et al. United States Supreme Court Update. *Appellate Advocate*. 2011;23:647-685. | Law Review | To summarize the Supreme Court's opinion in *Astra v. Santa Clara County* | Hospitals, clinics, manufacturers | "The Court determined the Agreement was a form contract that served primarily to enroll drug manufacturers into the 340B Program and spell out the terms of the statute and underlying regulations. The Court reasoned that, because the obligations under the Agreement and under the statute are identical, a suit to enforce the Agreement would be the same as a suit to enforce the statute. This conclusion was supported by the facts of the case, where the County's claims were based entirely on statutory obligations embodied in the Agreement, rather than any independent substantive obligation unique to the contract. The Court also rejected the Ninth Circuit's conclusion that third party beneficiary suits were an acceptable method of spreading the enforcement burden. The Court held this approach is inconsistent with the statutory framework, which centralizes enforcement power to avoid disjointed application of the closely related Medicaid Drug Rebate Program and 340B Program." | N/A | N/A |

© 2023 Knox RP et al. *JAMA Health Forum.*

| Article Citation | Article Type | Study Objective/Article Thesis | Stakeholders Discussed | Results/Analyses | Conclusions/ Recommendations | Limitations |
|---|---|---|---|---|---|---|
| Fisher NC. The 340B Program: a federal program in desperate need of revision after two-and-a-half decades of uncertainty. *J Health Care Law Policy*. 2019;22:25-73. | Law Journal | "this paper proposes by updating a few key areas, not only would program participants have better guidance, but the program's original intent would be preserved." | Hospitals, clinics, manufacturers, pharmacies, patients | Reforms are needed for the 340B Program. Congress needs to give additional oversight powers to HRSA after *PhRMA I* and *PhRMA II*. With these powers, HRSA needs to give guidance to covered entities on the definition of patients and the use of contract pharmacies. HRSA also needs to increase audits and require increased transparency to ensure the program is helping indigent patients. | "The 340B Program faces several internal challenges due to its nuanced and cumbersome nature. However, this paper has highlighted several key areas of the program, if updated, would have a huge impact… Tradeoffs are inevitable. However, any substantial change to the 340B Program should protect the original intent. Currently, competing incentives between drug manufacturers and covered entities have turned this well-intended program into a revenue generating arrangement. Still, the 340B Program has enabled covered entities to increase services provided. For some patients, these services may be the difference between life and death." | N/A |
| Geilfuss CF, Egan KJ, Kwiecinski MF. Access to the Section 340B Drug Pricing Program for newly eligible entities: critical next steps. *J Health Care Compliance*. 2010;12(4):39-64. | Legal Publication | To advise newly eligible 340B entities on participating in the 340B program | Hospitals, clinics | Newly eligible covered entities may be entitled to retroactive discounts on 340B drugs. Covered entities should consider contract pharmacy arrangements under HRSA's new guidance permitting an unlimited number of contract pharmacies. | N/A | N/A |

© 2023 Knox RP et al. *JAMA Health Forum.*

| Article Citation | Article Type | Study Objective/Article Thesis | Stakeholders Discussed | Results/Analyses | Conclusions/ Recommendations | Limitations |
|---|---|---|---|---|---|---|
| Haidar AZ. Recent 340B contract pharmacy troubles and the necessary solution. *Health Lawyer*. 2020;33(2):34-55 | Legal publication | "This article discusses the following: (1) the background of the 340B Program, (2) contract pharmacies, (3) recent actions taken by drug manufacturers that have endangered the practice of using contract pharmacies, (4) obstacles preventing remedies through enforcement by HRSA or through litigation, and (5) why and how Congress, HRSA, and CMS should act to solve the current problem." | Hospitals, clinics, manufacturers, pharmacies | "It is important that contract pharmacies continue to expand because of the savings and revenue that they bring to covered entities, especially covered entities like FQHCs which play an important role in providing care to low-income Americans. Covered entities use the savings and revenue that they generate through the 340B Program - including through contract pharmacy arrangements - to provide 340B drugs at lower costs to uninsured patients, eliminate the need for some patients to travel to receive care, and provide services that they might otherwise not have been able to offer to their patients.<br>At the same time, the concerns that drug manufacturers have with contract pharmacy arrangements, which largely stem from the lack of oversight by HRSA and the lack of clarity in state Medicaid policies, must also be addressed." | "To correct these deficiencies with the 340B Program, Congress should amend Section 340B to specifically grant authority to the HHS Secretary (through the HRSA Administrator) to promulgate regulations to enforce Section 340B. Congress should amend Section 340B to (1) specifically address contract pharmacy arrangements as a method through which drugs can be dispensed through the 340B Program, and (2) authorize HHS to promulgate binding regulations regarding contract pharmacy arrangements, which should include regulatory oversight of such arrangements by HHS through HRSA."<br><br>"Congress must directly address contract pharmacy arrangements and authorize the HHS Secretary to promulgate regulations governing them." | N/A |

© 2023 Knox RP et al. *JAMA Health Forum.*

| Article Citation | Article Type | Study Objective/Article Thesis | Stakeholders Discussed | Results/Analyses | Conclusions/ Recommendations | Limitations |
|---|---|---|---|---|---|---|
| Hodge JG, White EN, Freed R, Wells N. Supreme Court Impacts in Public Health Law: 2021-2022. J Law Med Ethics. 2022;50(3):608-612. doi: 10.1017/jme.2022.100. PMID: 36398641. | Law Journal | To describe "series of critical cases raise manifold changes and impacts on individual and communal health through 10 key areas ranging from abortions to vaccinations." | Hospitals, clinics, federal government | "In an unanimous Opinion released June 15, 2022 the Court held that HHS cannot alter reimbursement rates without complying with statutory requirements. Finding the agency exceeded its statutory authority in diminishing reimbursements allows many rural hospitals to retain over-payments on certain prescription drugs that help offset other expenses in treating indigent patients. The Court declined to address underlying principles of judicial deference to agencies' statutory interpretation, finding that the text of the federal statute was "straightforward." In June, as well, the Court deferred to HHS interpretation of a federal statute in determining disproportionate share hospital payments, potentially harming facilities serving low-income patients." | N/A | N/A |

© 2023 Knox RP et al. *JAMA Health Forum.*

| Article Citation | Article Type | Study Objective/Article Thesis | Stakeholders Discussed | Results/Analyses | Conclusions/ Recommendations | Limitations |
|---|---|---|---|---|---|---|
| Jackson T. A matter of interpretation: How the orphan drug litigation tests the limits of the 340B Program guidance: Orphan drug litigation exposes weaknesses in the 340B Program's statutory framework. *J Health Care Compliance*. 2014;16(5):5-16. | Legal Journal | "This article summarizes the orphan drug litigation, particularly the fight between HHS and PhRMA over whether the orphan drug rule can survive as interpretive guidance. The article then considers the standards that courts use to distinguish between legislative rules and interpretive guidance. Finally, this article considers whether certain existing 340B Program guidance is susceptible to challenge by pharmaceutical manufacturers or covered entities." | Hospitals, clinics, manufacturers, HRSA | "The orphan drug litigation exposes weaknesses in the 340B Program's statutory framework. Unless reversed on appeal, the District Court's decision indicates that HHS does not have the statutory authority to issue legislative rules that would provide clear, definitive standards for key aspects of 340B Program oversight. The orphan drug litigation also presents the question of whether HHS has appropriately used interpretive rules to address the "many gaps" that exist in the 340B Program. Because HHS may not have the authority to issue legislative rules and because limits exist on how far HHS may stretch interpretive rules, Congress should intervene to fill the gaps that exist in 340B Program oversight or expressly delegate to HHS the authority to do so." | N/A | N/A |

© 2023 Knox RP et al. *JAMA Health Forum.*

| Article Citation | Article Type | Study Objective/Article Thesis | Stakeholders Discussed | Results/Analyses | Conclusions/ Recommendations | Limitations |
|---|---|---|---|---|---|---|
| Junger J. High Impact, Unique Risks – What Compliance Professionals Need to Know About the 340B Drug Discount Program. *J Health Care Compliance.* 2022;24(2):37. | Legal Publication | To advise on compliance issues related to participation in the 340B Program | Hospitals, clinics, manufacturers, pharmacies | The article advises covered entities on the implementation of a 340B Program and compliance considerations related to duplicate discounting, diversion, virtual inventories, and contract pharmacy arrangements. | "The 340B Program may not be the first thought when identifying an organization's compliance risks, but it should not be an afterthought. A well-developed plan for 340B implementation can help safety-net providers keep the doors open and offer better and more comprehensive services to patients. Compliance professionals should consider reaching out to their Pharmacy counterparts and offer their support in ensuring that 340B savings continue to bolster the organization's safety-net mission." | N/A |
| Killelea A, Johnson J, Dangerfield DT, Beyrer C, McGough M, McIntyre J, Gee RE, Ballreich J, Conti R, Horn T, Pickett J, Sharfstein JM. Financing and Delivering Pre-Exposure Prophylaxis (PrEP) to End the HIV Epidemic. J Law Med Ethics. 2022;50(S1):8-23. doi: 10.1017/jme.2022.30. PMID: 35902089; PMCID: PMC9341207. | Law Journal | "This document proposes a financing and delivery system to unlock broad access to PrEP for those most vulnerable to HIV acquisition and bring an end to the HIV epidemic." | Hospitals, clinics, patients | The article proposes designing a national PrEP program and suggests new support for 340B providers to prevent overreliance on 340B revenue to fund HIV services. | "By building a bridge to often neglected communities, a national PrEP program could become a platform to address other major challenges to public health." | N/A |

© 2023 Knox RP et al. *JAMA Health Forum.*

| Article Citation | Article Type | Study Objective/Article Thesis | Stakeholders Discussed | Results/Analyses | Conclusions/ Recommendations | Limitations |
|---|---|---|---|---|---|---|
| Leifer JC, Evans MC, Graham RA, et al. Federally-qualified health centers: From safety net provider to cornerstone of health reform?. *J Health Life Sci Law*. 2014;8:29-62. | Law Journal | "This Comment discusses the evolution of the FQHC ""patient-centered health home" model of care and forecasts the central role FQHCs are likely to play in achieving significant changes to our health system over the coming years." | Clinics, patients, HRSA | "Under Section 340B of the Public Health Service Act, 20 covered entities, including FQHCs, can participate in a drug pricing program allowing them to purchase outpatient prescription drugs from manufacturers at the same discounted prices offered to State Medicaid agencies or at better prices if the FQHC can negotiate them. This reduces an FQHC's costs for medications it dispenses to patients and permits FQHCs "to stretch scarce Federal resources as far as possible, reaching more eligible patients and providing more comprehensive services." According to some estimates, FQHCs can realize savings of between 15% and 60% on prescription drug costs, depending on the volume and type of drugs purchased." | N/A | N/A |

© 2023 Knox RP et al. *JAMA Health Forum.*

| Article Citation | Article Type | Study Objective/Article Thesis | Stakeholders Discussed | Results/Analyses | Conclusions/ Recommendations | Limitations |
|---|---|---|---|---|---|---|
| Mello MM, Riley T, Sachs RE. The Role of State Attorneys General in Improving Prescription Drug Affordability. *S Cal L Rev*. 2022;95(3):595-662. | Law Journal | "In this Article, we report findings from an empirical study of state attorney general activities relating to pharmaceutical pricing. Drawing from key informant interviews with attorneys working on drug pricing issues as well as a scoping review, we report on how state attorneys general are working to address the problem of drug affordability, how they make decisions about resource investments in this area, what positions state attorneys general to be effective change agents in this space, and what challenges they confront in this work. We situate our results within the broader literature on state attorneys general as policy actors, and we suggest measures that could extend their capacity to successfully tackle the complex issues that give rise to unaffordable drugs." | Hospitals, clinics, pharmacies, patients, state governments, federal governments | State Attorneys General have played a significant role in actions related to prescription drug affordability. In one case, attorneys general from 28 states and the District of Columbia wrote a letter to the Trump Administration arguing that manufacturers were violating their obligations under the 340B Program by not delivering drugs to contract pharmacies. | "In conclusion, in reflecting on AGs as policy actors in our federalist system, Paul Nolette has argued that AGs can serve as "a mechanism to achieve a larger and more energetic regulatory state." Few areas of policymaking cry out for a more energetic regulatory presence than pharmaceutical pricing. Whether measured in terms of financial and clinical harm to patients, budgetary impact on public and private insurers, or starkness of contrast between the American approach and those of other industrialized nations, the regulatory gap is cavernous. AGs alone will not be able to close it, but they are and should be an integral part of the effort." | N/A |
| Ogrovsky K, Kracov DA. The impact of reform on health care fraud enforcement. *Brief*. 2010;40:42-48. | Legal Journal | To describe the impact of the Affordable Care Act on health care fraud enforcement | Hospitals, clinics, manufacturers | The Affordable Care Act reforms the 340B Program to require manufacturers provide quarterly reports on 340B ceiling prices and authorizes HRSA to issue civil monetary penalties for instances of knowing and intentional overcharging. | N/A | N/A |

© 2023 Knox RP et al. *JAMA Health Forum.*

| Article Citation | Article Type | Study Objective/Article Thesis | Stakeholders Discussed | Results/Analyses | Conclusions/ Recommendations | Limitations |
|---|---|---|---|---|---|---|
| Pepperdine University School of Law Legal Summaries. *J Natl Assoc Administrative Law Judiciary*. 2011;31:845-860. | Law Review | To summarize the Supreme Court's opinion in *Astra v. Santa Clara County* | Hospitals, clinics, manufacturers | "The Supreme Court held that a private action to enforce the price agreement made under the 340B program is incompatible with the statutory regime, because Congress vested authority to supervise compliance with the program solely with the Department of Health and Human Services. … Noting that the agreements had no negotiable terms, the Court reasoned that the pricing agreements are analogous to the Medicaid Rebate Agreements and are not traditional contracts. A third-party suit to enforce an agreement between the Department of Health and Human Services and drug manufactures, the Court concluded, is essentially a suit to enforce the statute itself, which all parties agreed Congress did not intend to permit." | N/A | N/A |
| Privacy & Publicity/Negligence/Equitable Remedies. *Bus Torts Rep*. 2021;34(2):400-43. | Law Journal | To describe the case J.R. v. Walgreens Boots Alliance, Inc., No. 20-1767 (4th Cir. Oct. 19, 2021) (per curiam). | Pharmacies, patients | Customers did not have privacy claims against Walgreens related to inclusion of patient prescription data to facilitate its participation in the 340B Program. | N/A | N/A |

© 2023 Knox RP et al. *JAMA Health Forum.*

| Article Citation | Article Type | Study Objective/Article Thesis | Stakeholders Discussed | Results/Analyses | Conclusions/ Recommendations | Limitations |
|---|---|---|---|---|---|---|
| Romano DH. Unlawful, unfair, and unwise: Constitutional and rulemaking infirmities in CMS's enrollment revocation regulations and how to challenge them. *Health Lawyer*. 2019;31(6):3-16. | Legal Publication | "This article looks at due process and other serious issues with how CMS's regulations are written and applied." related to revokation of Medicare participation." | Hospitals | "In the recent decision of AHA v. Azar, however, the district court waived exhaustion and took jurisdiction over the American Hospital Association's ("AHA's") challenge to CMS's 340B discount drug program payment rule, solely on the basis that the challenge to the regulation would have been futile if it were pursued through the administrative appeals process, and addressed the merits of the AHA's claim that the rule was ultra vires. …If the court's decision stands on appeal, it might pave the way for providers and suppliers to challenge the enrollment regulations as procedurally or substantively invalid without first exhausting administrative remedies and without even the need to present a collateral claim (provided that other courts would be persuaded by the D.C. courts)." | N/A | N/A |

© 2023 Knox RP et al. *JAMA Health Forum.*

| Article Citation | Article Type | Study Objective/Article Thesis | Stakeholders Discussed | Results/Analyses | Conclusions/ Recommendations | Limitations |
|---|---|---|---|---|---|---|
| Shepherd J. The prescription for rising drug prices: Competition or price controls?. *Health Matrix*. 2017;27:316-346. | Law Journal | "In this article, I explain why reforms promoting competition will produce better results and fewer negative consequences than reforms imposing new price controls." | Manufacturers | "HRSA estimates that covered entities saved $3.8 billion on outpatient drugs through the program in fiscal year 2013. Unfortunately, many of these savings are not reaching low-income patients. The 340B statute does not require that providers only dispense 340B drugs to needy patients. Instead, providers may purchase 340B drugs at a steep discount, sell them to non-qualified patients, and pocket the difference between the 340B discounted price and the reimbursement from the non-qualified patients' private insurance companies. …. Moreover, with at least forty percent of drugs sold under price controls, and some programs even requiring drugs to be sold for a penny, manufacturers are forced to sell many drugs at significant discounts. This creates incentives to charge higher prices to other, non-covered patients to offset the discounts." | Expanded adoption of price controls (like those used in the 340B Program) incentivizes higher drug prices, discourage innovations, and may stifle generic entry. | N/A |

© 2023 Knox RP et al. *JAMA Health Forum.*

| Article Citation | Article Type | Study Objective/Article Thesis | Stakeholders Discussed | Results/Analyses | Conclusions/ Recommendations | Limitations |
|---|---|---|---|---|---|---|
| Social Security Act – Administrative Law – Chevron Deference – American Hospital Ass'n v. Becerra. *Harv L Rev*. 2022;136:480-89. | Law Journal | To summarize the Supreme Court's opinion in *American Hospital Association v. Becerra* | Hospitals, clinics, federal government | The Supreme Court held that under the Medicare statute, HHS had to survey hospitals for drug acquisition cost data before varying reimbursement rates by hospital group. Therefore Medicare was not permitted to adjust the reimbursement rates for 340B hospitals without this survey data. The case adds to the Court's *Chevron* jurisprudence. | N/A | N/A |
| Vohra S, Pointer C, Fogleman A, Albers T, Patel A, Weeks E. Designing Policy Solutions to Build a Healthier Rural America. J Law Med Ethics. 2020 Sep;48(3):491-505. doi: 10.1177/1073110520958874. PMID: 33021172. | Law Journal | "This paper aims to provide a framework for policy solutions to build a healthier rural America." | Hospitals, clinics, patients | The 340B Program provides critical support to rural hospitals. Several other federal programs and designations also support health care services in rural communities. | Research into the needs of rural health communities and reforms with a focus on social issues (including economic development, housing, education, and culture) are needed to improve rural health. | N/A |
| von Oehesen W, Doggett G, Davis J. The 340B Drug Discount Program: A new era of enforcement and oversight. *J Health Life Sci Law*. 2012;5:72-123. | Law Journal | "Besides analyzing the history and impact of these two reports, the article offers recommendations on how 340B providers can prepare for increased program enforcement and how legislation intended to create a meaningful dispute resolution process must be implemented." | Hospitals, clinics, manufacturers, pharmacies | The article discusses the challenges with 340B compliance. In particular, the article reviews the changes made to the 340B Program by the Affordable Care Act, the changing definition of a patient under HRSA guidance, the challenges of preventing duplicate discounting, and the need for a dispute resolution process. | "As the community works together on these issues, there is a need to address the escalating threat of declining third-party reimbursement rates to 340B providers, which all stakeholders recognize inhibit the program's purpose. The entire 340B community will need to come together to ensure that stakeholders have effective enforcement mechanisms, including a meaningful administrative dispute resolution process, and that the 340B program operates with the utmost integrity." | N/A |

© 2023 Knox RP et al. *JAMA Health Forum.*

| Article Citation | Article Type | Study Objective/Article Thesis | Stakeholders Discussed | Results/Analyses | Conclusions/ Recommendations | Limitations |
|---|---|---|---|---|---|---|
| White EK. Killing U.S. Slowly: Curing the epidemic rise of cancer drug prices. *Food Drug Law J*. 2017;72:189-224. | Law Journal | This article "discusses various U.S. regulatory frameworks that impact prescription drug prices and evaluates proposed reforms to these frameworks in terms of which ones are most likely to succeed" focusing on cancer drugs. | Manufacturers | "Critics argue that basing Medicaid rebates and 340B discounts on AMP, as well as providing an additional Medicaid rebate for price increases, incentivizes manufacturers to set high initial market prices in order to increase the price paid by these programs. Others have argued that because the discount on brand-name drugs may be affected by the 'best price' offered by manufacturers, manufacturers are incentivized not to reduce prices charged to other purchasers because it may lower the price paid by Medicaid and 340B covered entities… Although also not proposed by MedPAC or HHS, Congress may be willing to implement a separate reimbursement model for physicians at 340B hospitals that more accurately tracks the deeply discounted prices they pay for prescription drugs. Given the rising number of 340B hospitals and the increased Medicare spending on chemotherapy drugs at these hospitals, changing the physician reimbursement model for 340B hospitals could curb the windfall they receive from the ASP plus 6% system." | "The United States must address the rising cost of prescription drugs and the profit-maximizing pricing practices of the pharmaceutical industry." | N/A |

© 2023 Knox RP et al. *JAMA Health Forum.*

| Article Citation | Article Type | Study Objective/Article Thesis | Stakeholders Discussed | Results/Analyses | Conclusions/ Recommendations | Limitations |
|---|---|---|---|---|---|---|
| **Westlaw – Legislative History** | | | | | | |
| 138 Cong. Rec. H11666-02, 1992 WL 280143 (1992). | Legislative History | Draft and debate of the Veterans Health Care Act of 1992 | Hospitals, clinics, manufacturers, patients | The report includes a draft of the Veterans Health Care Act of 1992 and discussion of the need for the 340B Program to resolve the unintended consequences of the Medicaid Drug Rebate Program and to help public health clinics and hospitals stretch scare federal funds by receiving discounts on drugs. Representative Slattery also expressed concern of the unintended consequences of the Medicaid Best Price Rule and argued for a flat rebate as an alternative. | N/A | N/A |
| 138 Cong. Rec. S16117-01, 1992 WL 251277 (1992). | Legislative History | Draft and debate of the Veterans Health Care Act of 1992 | Hospitals, clinics, manufacturers, patients | The report includes a draft of the Veterans Health Care Act of 1992 and discussion of the need for the 340B Program to resolve the unintended consequences of the Medicaid Drug Rebate Program and to help public health clinics and hospitals stretch scare federal funds by receiving discounts on drugs. | N/A | N/A |
| 138 Cong. Rec. S17742-02, 1992 WL 279425 (1992). | Legislative History | Debate on the proposed Veterans Health Care Act of 1992 | Hospitals, clinics, manufacturers, patients | The debate included speeches from Senators Chafee and Rockefeller reflecting on the unintended consequences of the Medicaid Drug Rebate Program and the need for the 340B Program to resolve these challenges. | N/A | N/A |

© 2023 Knox RP et al. *JAMA Health Forum.*

| Article Citation | Article Type | Study Objective/Article Thesis | Stakeholders Discussed | Results/Analyses | Conclusions/ Recommendations | Limitations |
|---|---|---|---|---|---|---|
| 138 Cong. Rec. S17872-02, 1992 WL 279559 (1992). | Legislative History | Draft and proposed amendments to the Veterans Health Care Act of 1992 and discussion of compromise agreements between the House of Representatives and Senate versions of the bill | Hospitals, clinics, manufacturers | Draft of the full Veterans Health Care Act creating the 340B Program. The Senate and House of Representatives compromised on the entities eligible to participate in the 340B Program, excluding , alcohol or drug treatment entities or mental health entities receiving Public Health Service funds and including comprehensive hemophilia diagnostic treatment center, Native Hawaiian Health Centers, and urban Indian organization receiving funds under title V of the Social Security Act. | N/A | N/A |
| 155 Cong. Rec. S11607-03, 2009 WL 3877374 (2009). | Legislative History | Proposed Senate amendments to H.R. 3590, to amend the Internal Revenue Code of 1986 to modify the first-time homebuyers credit in the case of members of the Armed Forces and certain other Federal employees, and for other purposes | Hospitals, clinics, manufacturers | The proposed amendments expanded the categories of hospitals and clinics eligible to participate in the 340B Program, allowed some inpatient drugs to receive 340B discounts, set restrictions on group purchasing arrangements, proposed improvements to program integrity, and required GAO study and make recommendations on improving the 340B Program. | N/A | N/A |

© 2023 Knox RP et al. *JAMA Health Forum.*

| Article Citation | Article Type | Study Objective/Article Thesis | Stakeholders Discussed | Results/Analyses | Conclusions/ Recommendations | Limitations |
|---|---|---|---|---|---|---|
| 155 Cong. Rec. S12072-03, 2009 WL 4280114 (2009). | Legislative History | Proposed Senate amendments to H.R. 3590, to amend the Internal Revenue Code of 1986 to modify the first-time homebuyers credit in the case of members of the Armed Forces and certain other Federal employees, and for other purposes | Hospitals, clinics | The proposal would permit manufacturers from providing discounts to 340B covered entities in exchange for inclusion on their formularies. | N/A | N/A |
| 155 Cong. Rec. S12164-01, 2009 WL 4340053 (2009). | Legislative History | Proposed Senate amendments to H.R. 3590, to amend the Internal Revenue Code of 1986 to modify the first-time homebuyers credit in the case of members of the Armed Forces and certain other Federal employees, and for other purposes | Hospitals, clinics, manufacturers | The proposed amendments expanded the categories of hospitals and clinics eligible to participate in the 340B Program, allowed some inpatient drugs to receive 340B discounts, set restrictions on group purchasing arrangements, proposed improvements to program integrity, and required GAO study and make recommendations on improving the 340B Program. | N/A | N/A |
| 155 Cong. Rec. S12334-01, 2009 WL 4405668 (2009). | Legislative History | Proposed Senate amendments to H.R. 3590, to amend the Internal Revenue Code of 1986 to modify the first-time homebuyers credit in the case of members of the Armed Forces and certain other Federal employees, and for other purposes | Hospitals, clinics | The proposal would permit manufacturers from providing discounts to 340B covered entities in exchange for inclusion on their formularies. | N/A | N/A |

© 2023 Knox RP et al. *JAMA Health Forum.*

| Article Citation | Article Type | Study Objective/Article Thesis | Stakeholders Discussed | Results/Analyses | Conclusions/ Recommendations | Limitations |
|---|---|---|---|---|---|---|
| 155 Cong. Rec. S12621-01, 2009 WL 4573078 (2009). | Legislative History | Proposed Senate amendments to H.R. 3590, to amend the Internal Revenue Code of 1986 to modify the first-time homebuyers credit in the case of members of the Armed Forces and certain other Federal employees, and for other purposes | Hospitals, clinics | The amendment proposed adding rural health clinics to the list of covered entities eligible to participate in the 340B Program. | N/A | N/A |
| 155 Cong. Rec. S12914-01, 2009 WL 4722527 (2009). | Legislative History | Proposed Senate amendments to H.R. 3590, to amend the Internal Revenue Code of 1986 to modify the first-time homebuyers credit in the case of members of the Armed Forces and certain other Federal employees, and for other purposes | Hospitals, clinics, manufacturers | The proposal would add Program of All-Inclusive Care for the Elderly (PACE) and Special Needs Programs (SNPs) to the list of covered entities eligible to participate in the 340B Program, exclude amendments to the 340B Program from certain budget neutral requirements of the Affordable Care Act and would permit manufacturers from providing discounts to 340B covered entities in exchange for inclusion on their formularies. | N/A | N/A |
| 155 Cong. Rec. S13096-04, 2009 WL 4733405 (2009). | Legislative History | Proposed Senate amendments to H.R. 3590, to amend the Internal Revenue Code of 1986 to modify the first-time homebuyers credit in the case of members of the Armed Forces and certain other Federal employees, and for other purposes | Hospitals, clinics, manufacturers | The proposal would permit manufacturers from providing discounts to 340B covered entities in exchange for inclusion on their formularies. | N/A | N/A |

© 2023 Knox RP et al. *JAMA Health Forum.*

| Article Citation | Article Type | Study Objective/Article Thesis | Stakeholders Discussed | Results/Analyses | Conclusions/ Recommendations | Limitations |
|---|---|---|---|---|---|---|
| 155 Cong. Rec. S13890-02, 2009 WL 5042748 (2009). | Legislative History | Proposed Senate amendments to H.R. 3590, Service Members Home Ownership Tax Act of 2009 | Hospitals, clinics, manufacturers | The proposed amendments expanded the categories of hospitals and clinics eligible to participate in the 340B Program, allowed some inpatient drugs to receive 340B discounts, set restrictions on group purchasing arrangements, proposed improvements to program integrity, and required GAO study and make recommendations on improving the 340B Program. | N/A | N/A |
| 156 Cong. Rec. H1891-01, 2010 WL 1027566 (2010). | Legislative History | Proposed Senate amendments to H.R. 3590, Service Members Home Ownership Tax Act of 2009, and H.R. 4872, Health Care and Education Reconciliation Act of 2010 | Hospitals, clinics, manufacturers | The proposed amendments expanded the categories of hospitals and clinics eligible to participate in the 340B Program, allowed some inpatient drugs to receive 340B discounts, set restrictions on group purchasing arrangements, proposed improvements to program integrity, and required GAO study and make recommendations on improving the 340B Program. | N/A | N/A |
| 156 Cong. Rec. H8267-06, 2010 WL 5017582 (2010). | Legislative History | Draft amendments to the Medicare and Medicaid Extenders Act of 2010 | Hospitals, clinics | The proposal would allow children's hospitals to purchase orphan drugs at 340B discounts. | N/A | N/A |

© 2023 Knox RP et al. *JAMA Health Forum.*

| Article Citation | Article Type | Study Objective/Article Thesis | Stakeholders Discussed | Results/Analyses | Conclusions/ Recommendations | Limitations |
|---|---|---|---|---|---|---|
| 156 Cong. Rec. S2028-01, 2010 WL 1064252 (2010). | Legislative History | Draft amendments to H.R. 8472 | Hospitals, clinics, manufacturers | The proposal would exclude amendments to the 340B Program from certain budget neutral requirements of the Affordable Care Act and would permit manufacturers from providing discounts to 340B covered entities in exchange for inclusion on their formularies. | N/A | N/A |
| 156 Cong. Rec. S8631-02, 2010 WL 4976844 (2010). | Legislative History | Draft of H.R. 4994, "An Act to amend the Internal Revenue Code of 1986 to reduce taxpayer burdens and enhance taxpayer protections, and for other purposes" | Hospitals, clinics | The proposal would allow children's hospitals to purchase orphan drugs at 340B discounts. | N/A | N/A |
| 156 Cong. Rec. S8642-03, 2010 WL 4976872 (2010). | Legislative History | Proposed amendments to bill S. 3454 | Hospitals, clinics | The proposal would allow children's hospitals to purchase orphan drugs at 340B discounts. | N/A | N/A |
| 1992 U.S.C.C.A.N. 4186, 1992 WL 475763 (Leg.Hist.) (1992). | Legislative History | Report reviewing the compromise agreement between the House of Representatives and Senate on the Veterans Health Care Act of 1992 | Hospitals, clinics, manufacturers | The Senate and House of Representatives compromised on the entities eligible to participate in the 340B Program, excluding , alcohol or drug treatment entities or mental health entities receiving Public Health Service funds and including comprehensive hemophilia diagnostic treatment center, Native Hawaiian Health Centers, and urban Indian organization receiving funds under title V of the Social Security Act. | N/A | N/A |

© 2023 Knox RP et al. *JAMA Health Forum.*

| Article Citation | Article Type | Study Objective/Article Thesis | Stakeholders Discussed | Results/Analyses | Conclusions/ Recommendations | Limitations |
|---|---|---|---|---|---|---|
| H.R. REP. 102-384(II), H.R. REP. 102-384, H.R. Rep. No. 384(II), 102ND Cong., 2ND Sess. 1992, 1992 WL 239341 (1992). | Legislative History | Draft of the Medicaid Drug Rebate Amendments of 1992, Veterans Health Care Act of 1992 and committee report | Hospitals, clinics, manufacturers, pharmacies, patients | The report discusses the discontinued discounts after the Medicaid Drug Rebate Program and the need to extend these discounts to the Veterans Health Administration, the Department of Defense, and many federally funded clinics and public hospitals serving uninsured patients. | N/A | N/A |
| H.R. REP. 111-443(I), H.R. REP. 111-443, H.R. Rep. No. 443, 111TH Cong., 2ND Sess. 2010, 2010 WL 3565221 (2010). | Legislative History | Draft of the Health Care and Education Reconciliation Act of 2010 with proposed amendments to the 340B Program | Hospitals, clinics | The draft legislation discusses how qualified health benefits plans on the health insurance exchange should contract with 340B covered entities. | N/A | N/A |
| H.R. REP. 111-443(II), H.R. REP. 111-443, H.R. Rep. No. 443(II), 111TH Cong., 1ST Sess. 2010, 2010 WL 6727115 (2010). | Legislative History | Draft of the Health Care and Education Reconciliation Act of 2010 with proposed amendments to the 340B Program | Hospitals, clinics | The draft legislation discusses how qualified health benefits plans on the health insurance exchange should contract with 340B covered entities. | N/A | N/A |
| H.R. REP. 111-448, H.R. Rep. No. 448, 111TH Cong., 2ND Sess. 2010, 2010 WL 1003032 (2010). | Legislative History | Draft of the Health Care and Education Reconciliation Act of 2010 with proposed amendments to the 340B Program | Hospitals, clinics | The draft legislation proposes adding restrictions on the use of group purchasing arrangements and discounts on orphan drugs in the 340B Program. | N/A | N/A |
| S. REP. 102-401, S. Rep. No. 401, 102ND Cong., 2ND Sess. 1992, 1992 WL 232830 (1992). | Legislative History | Draft of the Veterans Health Care Act of 1992 | Hospitals, clinics, manufacturers, pharmacies, patients | The report discusses the relationship between the 340B Program and the Medicaid Drug Rebate Program and the need for these mandatory discounts. | N/A | N/A |

© 2023 Knox RP et al. *JAMA Health Forum.*

| Article Citation | Article Type | Study Objective/Article Thesis | Stakeholders Discussed | Results/Analyses | Conclusions/ Recommendations | Limitations |
|---|---|---|---|---|---|---|
| **US Department of Health & Human Services Office of the Inspector General** | | | | | | |
| US Department of Health & Human Services Office of the Inspector General. Reasonable Assumptions in Manufacturer Reporting of AMPs and Best Prices. Published Sept. 2019. Accessed Apr. 10, 2023. https://oig.hhs.gov/oei/reports/oei-12-17-00130.pdf | Government Report | The objectives of the study were: "1. To identify the extent to which drug manufacturers make reasonable assumptions when calculating average manufacturer prices (AMPs) and best prices (BPs). 2. To identify the extent to which manufacturers would like greater guidance or instruction from the Centers for Medicare & Medicaid Services (CMS) on calculating AMP and BP. 3. To describe CMS's processes for overseeing the reasonable assumptions made by drug manufacturers in the Medicaid Drug Rebate Program (MDRP)." | Manufacturers | "Reasonable assumptions that manufacturers make when calculating average manufacturer prices and best prices can have large financial ramifications for the cost of prescription drugs to Medicaid and to safety-net providers. However, under current practices, CMS never assesses the majority of these assumptions. The agency believes that its statutory authority in this area is limited." | HHS-OIG recommended that "CMS (1) issue guidance related to specific areas identified in this report—specifically, value based purchasing agreements; (2) assess the costs and benefits of implementing a targeted process to review certain assumptions; and (3) implement a system to share responses to manufacturer inquiries for technical assistance." | The study relied on self-reported data from manufacturers and the sample size was not large enough to be generalizeable. |

© 2023 Knox RP et al. *JAMA Health Forum.*

| Article Citation | Article Type | Study Objective/Article Thesis | Stakeholders Discussed | Results/Analyses | Conclusions/ Recommendations | Limitations |
|---|---|---|---|---|---|---|
| US Department of Health & Human Services Office of the Inspector General. Medicare Part D Rebates for Prescriptions Filled at 340B Contract Pharmacies. Published July 2019. Accessed Apr. 10, 2023. https://oig.hhs.gov/oas/reports/region3/31600002.pdf | Government Report | "Our objective was to determine the rebate amount that could have been generated had pharmaceutical manufacturers and Part D sponsors agreed that prescriptions for Part D beneficiaries filled at 340B contract pharmacies would generate rebates for those sponsors." | Manufacturers, pharmacies | "If the Part D prescriptions for the sponsors in our review had been filled at non-340B pharmacies, sponsors calculated that manufacturers would have paid rebates of up to $74.7 million for 554,549 claims in 2014… We also found that because there are no 340B identifiers on claims and PDE records, sponsors do not have the data to distinguish whether prescriptions dispensed at a 340B contract pharmacy were filled using 340B drugs. Therefore, the possible additional rebate amount of up to $74.7 million is for both 340B and non-340B drugs filled at a 340B contract pharmacy for Part D beneficiaries. There is an opportunity to potentially reduce Part D costs if sponsors were to negotiate similar net prices for both non-340B drugs dispensed by 340B contract pharmacies and drugs dispensed by non-340B pharmacies." | N/A | N/A |

© 2023 Knox RP et al. *JAMA Health Forum.*

| Article Citation | Article Type | Study Objective/Article Thesis | Stakeholders Discussed | Results/Analyses | Conclusions/ Recommendations | Limitations |
|---|---|---|---|---|---|---|
| US Department of Health and Human Services Office of the Inspector General. Drug Supply Chain Security: Wholesalers Exchange Most Tracing Information. Published Sept. 2017. Accessed Apr. 10, 2023. https://oig.hhs.gov/oei/reports/oei-05-14-00640.pdf | Government Report | "To describe selected wholesalers' exchange of drug product tracing information in the drug supply chain as required by the Drug Supply Chain Security Act (DSCSA)." | Manufacturers, pharmacies | "We found that selected wholesalers were exchanging drug product tracing information and about half – including the three largest wholesalers that account for more than 80 percent of drug distribution revenues – exchange all required information. Complete drug product tracing information can improve drug supply chain security by supporting FDA and other State and Federal agencies' investigations of suspect and illegitimate drug products and potential diversion." | "To ensure that all wholesalers comply with the DSCSA, we recommend that FDA offer technical assistance where appropriate. Specifically, we recommend that FDA provide technical assistance to wholesalers regarding direct purchase statements, exempt drugs, and exchanging drug product tracing information for transactions involving 340B-covered entities and 340B contract pharmacies. FDA concurred with all of our recommendations." | The study was based on wholesalers' self-reported information. |

© 2023 Knox RP et al. *JAMA Health Forum.*

| Article Citation | Article Type | Study Objective/Article Thesis | Stakeholders Discussed | Results/Analyses | Conclusions/ Recommendations | Limitations |
|---|---|---|---|---|---|---|
| US Department of Health & Human Services Office of the Inspector General. Calculation of Potential Inflation-Indexed Rebates for Medicare Part B Drugs. Published Aug. 2017. Accessed Apr. 10, 2023. https://oig.hhs.gov/oei/reports/oei-12-17-00180.pdf | Government Report | Objectives were: "1. To estimate the amount of rebates in 2015 if pharmaceutical manufacturers had been required to pay inflation-indexed rebates for Part B drugs. 2. To identify implementation issues that would need to be addressed if such rebates were required." | Manufacturers | "An ASP-based rebate program for Medicare Part B drugs could have resulted in $1.4 billion in inflation-indexed rebates in 2015 for 64 high-expenditure drugs. An AMP-based rebate program for the same 64 drugs could have resulted in $1.8 billion in inflation-indexed rebates that same year. Several implementation issues related to claims and data would need to be addressed should Congress decide to establish a rebate program under Part B." | "The results of this current study build upon our original 2011 analysis. This analysis did not take into account how implementation of a Part B rebate requirement could affect beneficiary coinsurance obligations, beneficiary access to prescription drugs, and the overall pharmaceutical marketplace. Furthermore, we did not address the operational burden of implementing such a requirement. Any consideration of a rebate program should address the following administrative issues that may hinder rebate collections: the use of Healthcare Common Procedure Coding System codes, incorrect coding conversions, unavailable drug pricing data, and difficulties in identifying 340B-purchased drugs." | The study did not verify Part B claims for accuracy, restricted its study to 64 drugs, and did not consider manufacturer responses to changes in rebates. |

© 2023 Knox RP et al. *JAMA Health Forum.*

| Article Citation | Article Type | Study Objective/Article Thesis | Stakeholders Discussed | Results/Analyses | Conclusions/ Recommendations | Limitations |
|---|---|---|---|---|---|---|
| US Department of Health & Human Services Office of the Inspector General. State Efforts to Exclude 340B Drugs from Medicaid Managed Care Rebates. Published June 2016. Accessed Apr. 10, 2023. https://oig.hhs.gov/oei/reports/oei-05-14-00430.pdf | Government Report | Study objectives were: "1. To describe States' methods for identifying claims for 340B-purchased drugs paid through Medicaid managed care organizations (MCOs). 2. To identify potential vulnerabilities in States' methods for correctly collecting rebates and preventing duplicate discounts for drugs paid through MCOs." | Manufacturers | "We found that, to identify 340B drug claims and correctly collect rebates for MCO drugs, most States use methods that identify providers using 340B-purchased drugs. However, we found that these provider-level methods may not accurately identify all individual 340B drug claims, creating a risk of duplicate discounts and forgone rebates. By contrast, we found that methods that operate at the claim level can improve accuracy in identifying 340B drug claims, and thereby, help States correctly collect rebates." | "We recommend that the Centers for Medicare & Medicaid Services (CMS) require States to use claim-level methods to identify 340B claims. … We also recommend that the Health Resources and Services Administration (HRSA) clarify its guidance on preventing duplicate discounts for MCO drugs to align with this new requirement. HRSA concurred with our recommendation." | |

© 2023 Knox RP et al. *JAMA Health Forum.*

| Article Citation | Article Type | Study Objective/Article Thesis | Stakeholders Discussed | Results/Analyses | Conclusions/ Recommendations | Limitations |
|---|---|---|---|---|---|---|
| US Department of Health & Human Services Office of the Inspector General. Part B Payments for 340B-Purchased Drugs. Published Nov 2015. Accessed Apr. 10, 2023. https://oig.hhs.gov/oei/reports/oei-12-14-00030.pdf | Government Report | Objectives were: "1. To estimate Medicare Part B expenditures for outpatient drugs purchased by covered entities in 2013. 2. To estimate the amount by which Medicare Part B payment amounts exceeded 340B ceiling prices in 2013. 3. To estimate the amount by which Medicare spending could have been reduced in 2013 if Part B had been able to share in the savings attributable to 340B discounts." | Hospitals, clinics, manufacturers | "Medicare Part B and its beneficiaries paid $3.5 billion for 340B-purchased drugs in 2013. In the aggregate, Part B payment amounts were 58 percent more than the statutorily based 340B ceiling prices that year, which allowed covered entities to retain approximately $1.3 billion. The 340B statute does not restrict how covered entities may use these funds. The three shared-savings arrangements described in this report would have resulted in Medicare Part B savings of $162 million to $1.1 billion in 2013 while still providing covered entities with incentives to purchase those drugs through the 340B Program." | "OIG has produced an extensive body of work examining the 340B Program from various angles. As stakeholders debate the nature of 340B discounts and whether statutory changes should be made to enable Medicare and/or Medicaid to share in these savings, this report presents an independent analysis to inform the ongoing discussion and to support congressional and Administration decisionmakers' efforts in striking a balance among the needs of these vital programs." | Assumptions made in model may have resulted in "underestimates of provider acquisition costs, Part B expenditures for 340B-purchased drugs, and Medicare spending reductions under shared-savings methodologies." |

© 2023 Knox RP et al. *JAMA Health Forum.*

| Article Citation | Article Type | Study Objective/Article Thesis | Stakeholders Discussed | Results/Analyses | Conclusions/ Recommendations | Limitations |
|---|---|---|---|---|---|---|
| US Department of Health & Human Services Office of the Inspector General. Recommendation Follow-up Memorandum Report: States' Collection of Rebates for Drugs Paid Through Medicaid Managed Care Organizations Has Improved, OEI-05-14-00431. Published Sept. 16, 2015. Accessed Apr. 10, 2023. https://oig.hhs.gov/oei/reports/oei-05-14-00431.pdf | Government Report | To provide updates on the status of states collecting Medicaid managed care organization rebates | Hospitals, clinics, state governments | "OIG found that nearly all (35 of 37) States that pay for drugs through MCOs collected rebates for these drugs between July 1, 2013, and June 30,2014, as required by ACA. These 35 States collected $7.5 billion in MCO rebates during that time. This represents a significant improvement compared to OIG's previous finding that only 12 of 22 States paying for drugs through MCOs were collecting rebates for those drugs in 2011… Moreover, we found that nine States collected some eligible MCO rebates, but not all of them." | "CMS should continue to work to ensure that all States are invoicing and collecting MCO rebates." | "The findings are based on survey and interview responses provided by States. We did not verify the accuracy of the rebate amounts reported by States. The rebate amounts in this report were current as of December 2014, when we completed our data collection." |

© 2023 Knox RP et al. *JAMA Health Forum.*

| Article Citation | Article Type | Study Objective/Article Thesis | Stakeholders Discussed | Results/Analyses | Conclusions/ Recommendations | Limitations |
|---|---|---|---|---|---|---|
| US Department of Health & Human Services Office of the Inspector General. Medicaid Drug Rebate Dispute Resolution Could Be Improved. Published Aug. 2014. Accessed Apr. 10, 2023. https://oig.hhs.gov/oei/reports/oei-05-11-00580.pdf | Government Report | Study objectives were: "To determine: 1. the extent to which rebates are disputed under the Medicaid drug rebate program, 2. the causes of frequently occurring types of disputes, 3. challenges that States face in resolving disputes, 4. the measures that States and drug manufacturers believe will help prevent or resolve disputes." | Hospitals, clinics, manufacturers, pharmacies | "Twenty-nine of thirty-one States that could provide data estimated that only a small percentage of rebate dollars were disputed. The 12 selected States indicated that within this small percentage, certain types of disputes occur frequently. These States reported that poor-quality claims data lead to disputes regarding unit-of-measure conversions and physician-administered drugs. In addition, States reported that poor-quality data regarding ineligible drugs lead to disputes about drugs purchased at a discount under the 340B Drug Pricing Program and terminated drugs. The 12 selected States reported that once disputes are initiated, they struggle to provide the data necessary to resolve them. States reported difficulties in providing claims data or source data to help resolve disputes. Finally, the selected States and manufacturers expressed interest in greater CMS involvement in preventing and resolving disputes." | "We recommend that to help prevent and resolve drug rebate disputes, CMS (1) work with States to improve the quality of claims data submitted by providers and pharmacies, (2) help States obtain better data on ineligible drugs, (3) facilitate States' submission of standardized claims data, and (4) establish a stronger role in dispute resolution." | Data was self-reported. Findings cannot be extrapolated nationally or across all manufacturers. |

© 2023 Knox RP et al. *JAMA Health Forum.*

| Article Citation | Article Type | Study Objective/Article Thesis | Stakeholders Discussed | Results/Analyses | Conclusions/ Recommendations | Limitations |
|---|---|---|---|---|---|---|
| US Department of Health & Human Services Office of the Inspector General. Contract Pharmacy Arrangements in the 340B Program. Published Feb. 4, 2014. Accessed Apr. 10, 2023. https://oig.hhs.gov/oei/reports/oei-05-13-00431.pdf | Government Report | To review the impact of contract pharmacy arrangements on diversion and duplicate discounts in the 340B Program. | Hospitals, clinics, manufacturers, pharmacies | "To prevent diversion in their contract pharmacy arrangements, covered entities in our study use different methods to identify 340B-eligible prescriptions; in some cases, this leads to differing determinations of 340B eligibility across covered entities… Twenty-two of thirty covered entities reported that to prevent duplicate discounts, their contract pharmacies do not dispense 340B-purchased drugs to Medicaid beneficiaries… Although 8 of 30 covered entities reported that their contract pharmacies dispense 340B-purchased drugs to Medicaid beneficiaries, 6 did not report a method to prevent duplicate discounts… Eight covered entities do not offer the discounted 340B price to uninsured patients in any of their contract pharmacy arrangements… Almost all covered entities in our study monitor their contract pharmacy arrangements, but few have retained independent auditors as recommended in HRSA guidance." | N/A | "The results of this memorandum report are limited to the 30 covered entities selected in our purposive sample, and are not representative of or generalizable to other covered entities. We did not verify the accuracy of covered entities' or administrators' interview responses for this memorandum report, nor did we review the records of covered entities or contract pharmacies to identify instances of diversion or duplicate discounts." |

© 2023 Knox RP et al. *JAMA Health Forum.*

| Article Citation | Article Type | Study Objective/Article Thesis | Stakeholders Discussed | Results/Analyses | Conclusions/ Recommendations | Limitations |
|---|---|---|---|---|---|---|
| US Department of Health & Human Services Office of the Inspector General. States' Collection of Rebates For Drugs Paid Through Medicaid Managed Care Organizations. Published Sept. 2012. Accessed Apr. 10, 2023. [https://oig.hhs.gov/oei/reports/oei-03-11-00480.pdf](https://oig.hhs.gov/oei/reports/oei-03-11-00480.pdf) | Government Report | Objectives were: "1. To determine whether State Medicaid agencies (States) have collected drug utilization data from Medicaid Managed Care Organizations (MCO), as required by the Patient Protection and Affordable Care Act (ACA). 2. To examine the processes States used to invoice and collect rebates from manufacturers for drugs dispensed to beneficiaries enrolled in MCOs. 3. To determine how many States invoiced manufacturers for rebates for drugs dispensed to MCO beneficiaries. 4. To calculate the amount States collected in rebates from manufacturers for drugs dispensed to MCO beneficiaries, as well as how much went uncollected. 5. To determine whether States that do not currently pay for drugs through MCOs will change the structures of their drug programs as a result of the new rebate requirements." | Manufacturers, state governments | "Between April 1, 2010, and June 30, 2011, 18 of the 22 States using a carve-in approach collected all the data needed to invoice manufacturers for rebates from their MCOs, 3 collected data from a portion of their MCOs, and 1 never collected any drug utilization data. All but one State that used a carve-in approach performed some type of data verification check. Twelve of the twenty-two States using a carve-in approach invoiced manufacturers and collected $1.6 billion in rebates for utilization in the second quarter of 2010 through the second quarter of 2011. However, 10 of the 22 States did not invoice manufacturers and collect rebates because, for example, they had to complete programming changes to the systems that process MCO claims. Additionally, the rebate expansion has prompted five States that used the carve-out approach to change to a carve-in approach." | "We recommend that the Centers for Medicare & Medicaid Services (CMS) follow up with the 10 States that had not collected rebates for drugs dispensed to Medicaid MCO beneficiaries and take action to enforce rebate collection if necessary." | Analysis relied on survey data. |

© 2023 Knox RP et al. *JAMA Health Forum.*

| Article Citation | Article Type | Study Objective/Article Thesis | Stakeholders Discussed | Results/Analyses | Conclusions/ Recommendations | Limitations |
|---|---|---|---|---|---|---|
| US Department of Health & Human Services Office of the Inspector General. State Medicaid Policies and Oversight Activities Related To 340B-Purchased Drugs. Published June 2011. Accessed Apr. 10, 2023. https://oig.hhs.gov/oei/reports/oei-05-09-00321.pdf | Government Report | "To describe State Medicaid agencies' policies and oversight activities related to drugs purchased under the 340B Drug Discount Program." | Hospitals, clinics, manufacturers, state governments | "Approximately half of States have written 340B policies that direct covered entities to bill Medicaid at cost for 340B-purchased drugs… States do not have necessary pricing information to create prepay edits for 340B-purchased drugs; 20 States conduct postpay reviews to identify overpayments… Over half of States developed alternatives to the Medicaid Exclusion File to identify 340B claims and prevent duplicate discounts." | HHS-OIG recommended that CMS direct states to create written 340B policies, inform states about tools to identify claims for 340B drugs, and work with HRSA to improve the Medicaid Exclusion File. It also recommended HRSA share 340B ceiling price data with states. | The study relied on self-reported unverified data. |

© 2023 Knox RP et al. *JAMA Health Forum.*

| Article Citation | Article Type | Study Objective/Article Thesis | Stakeholders Discussed | Results/Analyses | Conclusions/ Recommendations | Limitations |
|---|---|---|---|---|---|---|
| US Department of Health & Human Services Office of the Inspector General. States' Collection of Medicaid Rebates for Physician-Administered Drugs. Published June 2011. Accessed Apr. 10, 2023. https://oig.hhs.gov/oei/reports/oei-03-09-00410.pdf | Government Report | Objectives were: "1. To determine the number of States that met Federal requirements for the collection of rebates for certain physician-administered drugs by June 30, 2009. 2. To estimate the dollar amount of rebates that States requested and collected from manufacturers for all physician-administered drugs in the first and second quarters of 2009. 3. To identify issues that prevented States from collecting rebates for all physician-administered drugs that were requested from and/or owed by manufacturers in the first and second quarters of 2009." | Hospitals, clinics, manufacturers | "By June 2009, 73 percent of responding States reported meeting or exceeding the DRA's requirement to collect rebates for certain physician-administered drugs… We could not determine the financial impact of collecting rebates for physician-administered drugs because of incomplete and potentially inaccurate data provided by States… Twenty-nine States reported difficulties with nonpayment of the requested rebates for physician-administered drugs…Thirty-one States had not implemented certain steps necessary for collecting rebates on all eligible physician-administered drugs purchased by 340B entities" | HHS-OIG recommended that CMS "Take action against States that do not meet the DRA's requirement to collect rebates on physician-administered drugs. Ensure that all State agencies are accurately identifying and collecting physician-administered drug rebates owed by manufacturers. Work with States to develop guidance for implementing edits that increase the efficiency of physician-administered drug claim reviews. Work with States to administer guidance to providers and Medicare contractors about the rebate requirements for physician-administered drugs. Ensure that the crosswalk file is complete, accurate, and identifies rebateable physician-administered drugs." | Findings were based on self-reported data from only 26 states. |

© 2023 Knox RP et al. *JAMA Health Forum.*

| Article Citation | Article Type | Study Objective/Article Thesis | Stakeholders Discussed | Results/Analyses | Conclusions/ Recommendations | Limitations |
|---|---|---|---|---|---|---|
| US Department of Health & Human Services Office of the Inspector General. Memorandum Report: Payment for Drugs Under the Hospital Outpatient Prospective Payment System, OEI-03-09-00420. Published Oct. 22, 2010. Accessed Apr. 10, 2023. https://oig.hhs.gov/oei/reports/oei-03-09-00420.pdf | Government Report | To compare "Medicare payment amounts for 32 selected separately payable drugs covered under the hospital Outpatient Prospective Payment System (OPPS) to hospital acquisition costs." | Hospitals, manufacturers | "We found that, in the aggregate, Medicare payments were 31 percent higher than acquisition costs among responding 340B hospitals and 1 percent higher than acquisition costs among responding non-340B hospitals for the selected separately payable drugs. For the individual drugs, the Medicare payment amounts for more than half exceeded non-340B hospital acquisition costs. The Medicare payment amounts for the remaining drugs purchased by non-340B hospitals were below average acquisition costs, albeit by a small amount (between 0.6 percent and 11 percent)." | "The Medicare payment amount for separately payable drugs under the OPPS is based on manufacturer-reported ASPs. Because ASPs do not reflect 340B prices (which are generally lower than other sales prices), a substantial majority of the drugs purchased by 340B hospitals have Medicare payment amounts that are higher than average acquisition costs. Similarly, because non-340B hospitals do not have access to reduced prices, the ASP-based Medicare payment amounts are closer to their acquisition costs. Although for some individual drugs the Medicare payment amount was slightly below average acquisition costs at non-340B hospitals, the fact that overall payment levels exceeded costs leads us to conclude that stakeholder concerns about underpayment were unfounded." | The study based on self-reported data. |

© 2023 Knox RP et al. *JAMA Health Forum.*

| Article Citation | Article Type | Study Objective/Article Thesis | Stakeholders Discussed | Results/Analyses | Conclusions/ Recommendations | Limitations |
|---|---|---|---|---|---|---|
| US Department of Health & Human Services Office of the Inspector General. Drug Manufacturers' Noncompliance With Average Manufacturer Price Reporting Requirements. Published Sept. 2010. Accessed Apr. 10, 2023. https://oig.hhs.gov/oei/reports/oei-03-09-00060.pdf | Government Report | Objectives were: "1. To determine whether manufacturers submitted 2008 average manufacturer prices (AMP) to the Centers for Medicare & Medicaid Services (CMS) within the timeframes specified by Federal requirements. 2. To determine whether CMS has taken action against manufacturers that did not submit AMP data within the timeframes specified by Federal requirements." | Manufacturers | "In 2008, more than half of manufacturers did not fully comply with quarterly submission requirements for AMP data… In 2008, more than three-fourths of manufacturers did not fully comply with monthly submission requirements for AMP data… CMS took action against some manufacturers for failure to comply with quarterly AMP reporting requirements but took no action for failure to comply with monthly reporting requirements." | HHS-OIG recommended that CMS take action against manufacturers that submit incomplete quarterly AMP data and fail to submit monthly AMP data in a timely manner. | The study did not verify the data related to a covered outpatient drug, did not review corrected or resubmitted data, did not examine how manufacturers calculated late rebates, and did not contact manufacturers about discontinued NDCs. |
| US Department of Health & Human Services Office of the Inspector General. Review of 340B Prices. Published July 2006. Accessed Apr. 10, 2023. https://oig.hhs.gov/oei/reports/oei-05-02-00073.pdf | Government Report | "To determine whether entities participating in the 340B Drug Pricing Program pay more than the statutorily defined 340B ceiling prices and, if so, the potential reasons for price discrepancies." | Hospitals, clinics, manufacturers | "In June 2005, 14 percent of total purchases made by 340B entities exceeded the 340B ceiling prices, resulting in total overpayments of $3.9 million… The largest overpayments in our sample resulted from inappropriate handling of negative ceiling prices… Patterns in our sample suggest that overpayments varied by the volume of 340B purchases or sales associated with entities, manufacturers, and wholesalers… Inaccuracies in HRSA's ceiling prices limit its ability to monitor 340B program compliance." | "HRSA should improve its oversight of the 340B Program to ensure that entities are charged at or below the 340B ceiling price… HRSA should provide technical assistance regarding 340B Program implementation to all participating entities, manufacturers, and wholesalers… HRSA should publish guidance regarding its penny price policy… To accurately calculate 340B ceiling prices, HRSA should obtain data on consistent unit of measure and package size." | N/A |

© 2023 Knox RP et al. *JAMA Health Forum.*

| Article Citation | Article Type | Study Objective/Article Thesis | Stakeholders Discussed | Results/Analyses | Conclusions/ Recommendations | Limitations |
|---|---|---|---|---|---|---|
| US Department of Health & Human Services Office of the Inspector General. Determining Average Manufacturer Prices for Prescription Drugs Under the Deficit Reduction Act of 2005 (A-06-06-00063). Published May 30, 2006. Accessed Apr. 10, 2023. https://oig.hhs.gov/oas/reports/region6/60600063.pdf | Government Report | "Our objective was to review the requirements for, and manner in which, manufacturers determine AMPs under section 1927 of the Act." | Manufacturers | "Existing requirements for determining certain aspects of AMPs are not clear and comprehensive, and manufacturers' methods of calculating AMPs are inconsistent. OIG's previous and ongoing work, which has primarily focused on how manufacturers calculate AMP, has found that the manufacturers reviewed interpret AMP requirements differently. Specifically, our findings demonstrate the need to clarify the definition of retail class of trade and the treatment of pharmacy benefit manager rebates and Medicaid sales in AMP calculations. In addition, work related to the use of AMP by CMS and other agencies highlights the need to consider the timeliness and accuracy of manufacturer-reported AMPs. … Further, they raised additional issues related to the implementation of DRA provisions. Because the DRA expands the use of AMPs and creates new reimbursement policy implications, future errors or inconsistencies in manufacturers' AMP calculations could lead to inaccurate or inappropriate reimbursement amounts as well as rebate errors." | HHS-OIG recommended that CMS clarify guidance on AMPs, including the definition of retail class of trade and the treatment of PBM rebates and Medicaid sales and issue guidance on implementation of AMP-related reimbursement provisions of the DRA. | N/A |

© 2023 Knox RP et al. *JAMA Health Forum.*

| Article Citation | Article Type | Study Objective/Article Thesis | Stakeholders Discussed | Results/Analyses | Conclusions/ Recommendations | Limitations |
|---|---|---|---|---|---|---|
| US Department of Health & Human Services Office of the Inspector General. Deficiencies in the Oversight of the 340B Drug Pricing Program. Published Oct. 2005. Accessed Apr. 10, 2023. https://oig.hhs.gov/oei/reports/oei-05-02-00072.pdf | Government Report | "To assess the ability of the Health Resources and Services Administration (HRSA) to ensure that entities participating in the 340B Drug Pricing Program are able to purchase products at or below a statutorily established ceiling price." | Hospitals, clinics, HRSA | "Due to systemic problems with the accuracy and reliability of the Government's record of 340B ceiling prices, HRSA is unable to appropriately oversee the 340B Program… HRSA lacks the oversight mechanisms and authority to ensure that 340B entities pay at or below the 340B ceiling price… Participating entities cannot independently verify that they receive the correct 340B discount due to confidentiality provisions." | "CMS and HRSA should work together to ensure accurate and timely pricing data for the Government's official record of 340B ceiling prices… HRSA should establish detailed standards for its calculation of 340B ceiling prices… HRSA should institute oversight mechanisms to validate its 340B price calculations and the prices charged to participating entities… HRSA should seek authority to establish penalties for PHS Act violations… HRSA should provide participating entities with secure access to certain pricing data to help approximate 340B ceiling prices." | "We did not review CMS's oversight of manufacturer-reported prices required by OBRA '90. We did not audit the accuracy of manufacturers' reported prices. We relied on CMS's analysis of 340B ceiling prices for the first quarter of 2005, verifying and testing its conclusions where possible. Lastly, we did not assess whether price discrepancies or overcharges are occurring." |

© 2023 Knox RP et al. *JAMA Health Forum.*

| Article Citation | Article Type | Study Objective/Article Thesis | Stakeholders Discussed | Results/Analyses | Conclusions/ Recommendations | Limitations |
|---|---|---|---|---|---|---|
| US Department of Health & Human Services Office of the Inspector General. Deficiencies in the 340B Drug Discount Program's Database. Published June 2004. Accessed Apr. 10, 2023. https://oig.hhs.gov/oei/reports/oei-05-02-00071.pdf | Government Report | Objective was to "assess the accuracy of information contained in the Pharmacy Affairs Branch (PAB) 340B Drug Discount Program's database." | Hospitals, clinics, HRSA | "Thirty-eight percent of our sampled entities were listed as enrolled in the 340B database, but reported not participating in the program. The database also had incorrect addresses for 43 percent of sampled entities. We also found that the database does not provide essential information on the entities' billing and shipping arrangements. According to interviews with nine major pharmaceutical corporations, the extent of incorrect addresses listed in the database hinders their ability to effectively identify entities eligible for the discount program." | "We recommend that HRSA develop a strategic plan for improved management of the 340B database. We suggest this plan include: (1) a revalidation of all current information in the database (2) an annual recertification process for entities participating in the discount program (3) a separate listing of newly added or deleted entities (4) a standard reporting format for entities' addresses and (5) an additional field to designate entities with contracted pharmacy arrangement." | N/A |
| US Department of Health & Human Services Office of the Inspector General. Medicaid's Mental Health Drug Expenditures. Published Aug. 2003. Published Apr. 10, 2023. https://oig.hhs.gov/oei/reports/oei-05-02-00080.pdf | Government Report | "To measure the efficiency and economy of the Medicaid program as a purchaser of mental health drugs by comparing Medicaid to four other Federal payers." | State Medicaid programs | "The 10 State Medicaid agencies reviewed paid more than other Government purchasers for the 25 mental health drugs reviewed… As a result of price differences, the 10 State Medicaid agencies paid, on average, between $47 and $126 million more for the 25 mental health drugs than other Federal purchasers." | "To safeguard the Medicaid program from excessive payments and capitalize on potential savings, we encourage CMS to reconsider previous OIG recommendations. In past reports, the OIG has recommended that CMS work with States to pursue more efficient means of purchasing pharmaceuticals and initiate a review of the Medicaid rebate program. We also suggest that CMS share this report with the States." | N/A |

© 2023 Knox RP et al. *JAMA Health Forum.*

| Article Citation | Article Type | Study Objective/Article Thesis | Stakeholders Discussed | Results/Analyses | Conclusions/ Recommendations | Limitations |
|---|---|---|---|---|---|---|
| US Department of Health & Human Services Office of the Inspector General. Review of Hemophilia Treatment Centers' Disposition of Program Income and Patient Choice for Factor Provider for Calendar Year 2000. Published June 17, 2003. Accessed Apr. 10, 2023. https://oig.hhs.gov/oas/reports/region3/30100350.pdf | Government Report | Objectives were to: "Assess the disposition of program income earned on sales of factor at prices in excess of the 340B acquisition price; Determine how HTCs billed Medicaid for reimbursement; Evaluate the adequacy of patient choice policies; and Determine pharmacy costs and bad debt expense." | Clinics | "The HTCs generally used program income for patient care and related activities, and had choice policies in place that allowed patients to obtain the factor they needed from providers of their choice. At one of the six HTCs we visited, however, we identified the following problems:<br>• Inappropriate use of program income; and<br>• Inappropriate Medicaid billing practices, resulting in overbilling of $613,000. We believe these problems might have been prevented by improved monitoring by HRSA's Maternal and Child Health Bureau (MCHB), which oversees the HTCs." | HHS-OIG recommended that HRSA:<br>"1. Develop program guidelines, which, at a minimum, include the disposition of program funds and conflicts of interest provisions.<br>2. Continue to monitor HTCs participating in the 340B program, and increase the areas of monitoring to include the conditions described in this report as a means of ensuring that program funds are used for their intended purpose and in accordance with applicable regulations and cost principles.<br>3. Emphasize to grantees that HTCs need to adhere to federal regulations limiting Medicaid reimbursement to the acquisition cost of factor plus a reasonable dispensing fee established by the state Medicaid agency.<br>4. Work with the Centers for Medicaid and Medicare Services (CMS) to ensure that the Medicaid overpayment of approximately $613,000 identified in this report is refunded to the respective state Medicaid program." | N/A |

© 2023 Knox RP et al. *JAMA Health Forum.*

| Article Citation | Article Type | Study Objective/Article Thesis | Stakeholders Discussed | Results/Analyses | Conclusions/ Recommendations | Limitations |
|---|---|---|---|---|---|---|
| US Department of Health & Human Services Office of the Inspector General. Pharmaceutical Manufacturers Overcharged 340B-Covered Entities. Published Mar. 10, 2003. Accessed Apr. 10, 2023. https://oig.hhs.gov/oas/reports/region6/60100060.pdf | Government Report | "the objectives of our review were to determine: (1) whether pharmaceutical manufacturers sold prescription drugs to 340B-covered entities using the correct Medicaid rebate amount; and (2) the extent of any overcharges." | Manufacturers | "We estimated that five manufacturers, makers of the 11 prescription drugs in our review, overcharged 340B-covered entities $6.1 million for sales occurring during the 1-year period ending September 30, 1999. The overcharges occurred because the drug manufacturers inappropriately excluded sales to health maintenance organization (HMO) repackagers from their best price determinations, thereby increasing the prices charged to 340B entities." | "We are recommending that the Health Resources and Services Administration (HRSA) require the five drug manufacturers to identify the exact amount of the overcharges for each of the affected 340B-covered entities and apply the overcharge amounts as offsets or credits to each entity's future purchases." | N/A |
| US Department of Health & Human Services Office of the Inspector General. Cost Containment of Medicaid HIV/AIDS Drug Expenditures. Published July 2001. Accessed Apr. 10, 2023. https://oig.hhs.gov/oei/reports/oei-05-99-00611.pdf | Government Report | The objective was "To compare the amount that Medicaid reimburses for HIV/AIDS drugs to the prices paid by other government purchasers." | Medicaid | "Medicaid pays up to 33 percent more than other Federal Government drug discount programs for HIV/AIDS drugs… Differences in Federal drug pricing formulas are partially responsible for cost discrepancies… State reimbursement formulas affect the magnitude of the gap between Medicaid and other government drug purchasers… Medicaid could have saved $102 million if the 10 States surveyed purchased the 16 antiretrovirals at Federal ceiling prices." | "For the 16 HIV/AIDS drugs examined, Centers for Medicare and Medicaid Services should review the current reimbursement methodology and work with States to find a method that more accurately estimates pharmacy acquisition cost… The Centers for Medicare and Medicaid Services should initiate a review of Medicaid rebates for the 16 HIV/AIDS drugs examined." | N/A |

© 2023 Knox RP et al. *JAMA Health Forum.*

| Article Citation | Article Type | Study Objective/Article Thesis | Stakeholders Discussed | Results/Analyses | Conclusions/ Recommendations | Limitations |
|---|---|---|---|---|---|---|
| US Department of Health & Human Services Office of the Inspector General. AIDS Drug Assistance Program Cost Containment Strategies. Published Sept. 2000. Accessed Apr. 10, 2023. https://oig.hhs.gov/oei/reports/oei-05-99-00610.pdf | Government Report | The goal of the study was to "Compare the prices that State AIDS Drug Assistance Programs are paying for drugs to the Federal ceiling prices listed in the Federal Supply Schedule." | Clinics | "ADAP ceiling prices are, on average, 16 percent higher than the Federal ceiling prices… ADAPs could have saved nearly $58 million in 1999 if allowed to purchase the 10 drugs at Federal ceiling prices." | "The Health Resources and Services Administration (HRSA) should seek legislation to change the 340B ceiling price calculation to the Federal ceiling price calculation…<br>To allow ADAPs to negotiate the lowest prices possible, HRSA should seek legislation to exempt all sales to 340B covered entities from the calculation of Non-Federal Average Manufacturers Price…<br>HRSA should continue to work with rebate and non-participating ADAPs to devise ways to grant them access to up-front drug discounts." | N/A |

© 2023 Knox RP et al. *JAMA Health Forum.*

| Article Citation | Article Type | Study Objective/Article Thesis | Stakeholders Discussed | Results/Analyses | Conclusions/ Recommendations | Limitations |
|---|---|---|---|---|---|---|
| US Department of Health & Human Services Office of the Inspector General. Audit of Comprehensive Hemophilia Treatment Centers' Utilization of the Public Health Service 340B Drug Pricing Program. Published Dec. 10, 1999. Accessed Apr. 10, 2023. https://oig.hhs.gov/oas/reports/region1/19801505.pdf | Government Report | "The objective of this audit was to determine whether HTCs participating in the 340B Program were participating for all of their patients, including Medicaid beneficiaries." | Clinics, Medicaid | "Officials from the majority of the 23 participating HTCs informed us that their HTCs purchase drugs for all outpatients, including Medicaid beneficiaries, at 340B discount prices. However, officials from 6 of the 23 participating HTCs contacted stated that their entities participate (purchase outpatient drugs at the 340B discount price), but not for their Medicaid beneficiaries... we determined that a State Medicaid Agency could achieve annual savings ranging from $18,395 to $27,170 per person if it reimbursed the HTC at the 340B discount prices instead of the Medicaid rate." | "We recommend that HRSA and HCFA work together to achieve a fair and equitable resolution of the issues involving the economical purchasing, and subsequent Medicaid billing, of covered drugs by entities participating in the 340B Program." | N/A |
| US Department of Health & Human Services Office of the Inspector General. Audit of the Utilization of the Public Health Service 340B Drug Pricing Program. Published July 16, 1998. Accessed Apr. 10, 2023. https://oig.hhs.gov/oas/reports/region1/19801500.pdf | Government Report | "The objective of this audit was to determine whether eligible Public Health Service funded entities effectively utilized the 340B Program." | Hospitals, clinics | "The database indicates that approximately 66 percent (2,351 of 3,574) of eligible HRSA grantees do not participate in the 340B Program." | "We commend the HRSA for its efforts and recommend that the HRSA:<br>Continue its efforts to require eligible entities to participate in the 340B Program, and<br>Periodically inform us of the status of these efforts." | N/A |

© 2023 Knox RP et al. *JAMA Health Forum.*

| Article Citation | Article Type | Study Objective/Article Thesis | Stakeholders Discussed | Results/Analyses | Conclusions/ Recommendations | Limitations |
|---|---|---|---|---|---|---|
| US Department of Health & Human Services Office of the Inspector General. Audit of State AIDS Drug Assistance Programs' Use of Drug Price Discounts. Published Jan. 2, 1998. Accessed Apr. 10, 2023. https://oig.hhs.gov/oas/reports/region1/19701501.pdf | Government Report | "The objective of this performance audit was to determine whether HRSA ensured that ADAPs effectively utilized available discount drug pricing programs." | Clinics | "While ADAPs utilized various cost savings strategies, 34 of 53 ADAPs did not participate in the 340B Drug Pricing Program (nonparticipating ADAPs) in Fiscal Year (FY) 1996… Eight of the ten highest dollar volume nonparticipating ADAPs informed us they did not participate because the 340B implementing guidelines limiting an entity to one contract pharmacy service provider are too restrictive…" | HHS-OIG recommended that HRSA "(1) require ADAPs to develop drug purchasing and distribution mechanisms that enable participation in the 340B Drug Pricing Program unless the ADAPs demonstrate that participation is not cost efficient or not possible under ODP's current guidelines; (2) develop new guidelines to allow ADAPs to participate in the 340B Drug Pricing Program with multiple pharmacy service providers (contract or otherwise) and work with ADAPs to implement the new guidelines; (3) explore alternatives for funding reviews to deter and detect drug diversion; (4) work with HCFA to devise a mechanism to share 340B ceiling prices or rebates (without disclosing AMPS) with eligible entities which express a need for the information (e.g., perform cost effectiveness analysis and verify the receipt of appropriate rebates or 340B ceiling prices) while maintaining confidentiality; and (5) continue its efforts to finalize guidelines establishing a rebate option for ADAPs." | N/A |

© 2023 Knox RP et al. *JAMA Health Forum.*

| Article Citation | Article Type | Study Objective/Article Thesis | Stakeholders Discussed | Results/Analyses | Conclusions/ Recommendations | Limitations |
|---|---|---|---|---|---|---|
| **Government Accountability Office** | | | | | | |
| US Government Accountability Office. Drug Pricing Program: HHS Uses Multiple Mechanisms to Help Ensure Compliance with 340B Requirements. Published Dec. 14, 2020. Accessed Apr. 10, 2023. https://www.gao.gov/products/gao-21-107 | Government Report | "This report describes (1) the audit findings that HRSA issued to address covered entity noncompliance with 340B Program requirements; and (2) other efforts HRSA uses to help ensure that covered entities comply with 340B Program requirements." | Hospitals, clinics, manufacturers, pharmacies, patients | GAO's study found HRSA audits identified thousands of instances of noncompliance with 340B requirements, including eligibility, recordkeeping, duplicate discounting, and diversion. | GAO recommended HRSA provide auditors with more specific guidance in conducting audits and require covered entities demonstrate that they are eligible and apply consequences for noncompliance. | N/A |
| US Government Accountability Office. 340B Drug Discount Program: Oversight of the Intersection with the Medicaid Drug Rebate Program Needs Improvement. Published Jan. 21, 2020. Accessed Apr. 10, 2023. https://www.gao.gov/products/gao-20-212 | Government Report | "GAO was asked to provide information on the prevention of duplicate discounts. Among other things, this report examines HHS's efforts to ensure compliance with the prohibition on duplicate discounts." | Hospitals, clinics, manufacturers, pharmacies, patients | GAO found limited oversight of the intersection between the 340B Program and the Medicaid Drug Rebate Program. In particular, CMS does not review Medicaid policies to prevent duplicate discounts, HRSA audits do not review state Medicaid policies to identify duplicate discounts, and HRSA has not issued guidance on preventing duplicate discounting in Medicaid managed care programs. | "GAO is making three recommendations, namely that: 1) CMS ensure that state Medicaid programs have written policies and procedures that are designed to prevent duplicate discounts and forgone rebates; and that HRSA 2) incorporate covered entities' compliance with state policies into its audits, and 3) require covered entities to work with manufacturers regarding repayment of identified duplicate discounts in managed care." | N/A |

© 2023 Knox RP et al. *JAMA Health Forum.*

| Article Citation | Article Type | Study Objective/Article Thesis | Stakeholders Discussed | Results/Analyses | Conclusions/ Recommendations | Limitations |
|---|---|---|---|---|---|---|
| US Government Accountability Office. 340B Drug Discount Program: Increased Oversight Needed to Ensure Nongovernmental Hospitals Meet Eligibility Requirements. Published Dec. 11, 2019. Accessed Apr. 10, 2023. https://www.gao.gov/products/gao-20-108 | Government Report | "GAO was asked to provide information on 340B-participating hospitals' contracts with state and local governments. This report (1) describes any obligations in selected nongovernmental hospitals' contracts to serve low-income individuals, and (2) examines HRSA's processes to assess nongovernmental hospitals' eligibility." | Hospitals, clinics | GAO found limitations in HRSA audits of nongovernmental hospitals. In particular, GAO found HRSA does not review contracts to ensure hospital eligibility, HRSA allows contracts with retroactive dates when noncompliance is found in the contracts, and HRSA does not ensure hospital contracts include provisions on caring for 340B-specified low-income populations. | "GAO is making six recommendations, including that HRSA implement a process to verify that all nongovernmental hospitals have contracts in place, including throughout hospitals' audit periods; amend its contract reviews to include an assessment of whether contracts meet statutory requirements; and provide better guidance on contract reviews. HHS concurred with all of the recommendations except the one to implement a process to verify that all nongovernmental hospitals have contracts." | N/A |

© 2023 Knox RP et al. *JAMA Health Forum.*

| Article Citation | Article Type | Study Objective/Article Thesis | Stakeholders Discussed | Results/Analyses | Conclusions/ Recommendations | Limitations |
|---|---|---|---|---|---|---|
| US Government Accountability Office. Drug Discount Program: Federal Oversight of Compliance at 340B Contract Pharmacies Needs Improvement. Published June 21, 2018. Accessed Apr. 10, 2023. https://www.gao.gov/products/gao-18-480 | Government Report | "GAO was asked to provide information on the use of contract pharmacies. Among other things, this report: 1) describes financial arrangements selected covered entities have with contract pharmacies; 2) describes the extent that selected covered entities provide discounts on 340B drugs dispensed by contract pharmacies to low-income, uninsured patients; and 3) examines HRSA's efforts to ensure compliance with 340B Program requirements at contract pharmacies." | Hospitals, clinics, manufacturers, pharmacies, patients | GAO found that HRSA audits do not assess compliance with the prohibition on duplicate discounting at contract pharmacies, covered entities are responsible for reporting the scope of noncompliance found in audits, and HRSA does not require evidence that covered entities take corrective action following audits. | GAO recommended HRSA require covered entities register contract pharmacies for each site of the covered entity, issue guidance on preventing duplicate discounts in Medicaid managed care, incorporate review of compliance with the prohibition of duplicate discounting in Medicaid managed care in its audit process, provide guidance on the length of time covered entities must look at in identifying the scope of noncompliance, require covered entities specify their methodology for identifying noncompliance in audits, require covered entities provide evidence of correction action prior to closing audits, and provide guidance to covered entities on contract pharmacy oversight. | N/A |
| US Government Accountability Office. Medicare Part B Drugs: Action Needed to Reduce Financial Incentives to Prescribe 340B Drugs at Participating Hospitals. Published June 5, 2015. Accessed Apr. 10, 2023. https://www.gao.gov/products/gao-15-442 | Government Report | "GAO was asked to review hospitals' participation in the 340B and Medicare programs. This report (1) compares 340B hospitals with non-340B hospitals in terms of financial and other characteristics and (2) compares spending for Medicare Part B drugs at 340B hospitals, for all drugs and for oncology drugs, with spending at non- 340B hospitals." | Hospitals | GAO found that per beneficiary Medicare Part B drug spending, including oncology drug spending, was higher at 340B disproportionate share hospitals than non-340B hospitals in both 2008 and 2012. | "Congress should consider eliminating the incentive to prescribe more drugs or more expensive drugs than necessary to treat Medicare Part B beneficiaries at 340B hospitals." | N/A |

© 2023 Knox RP et al. *JAMA Health Forum.*

| Article Citation | Article Type | Study Objective/Article Thesis | Stakeholders Discussed | Results/Analyses | Conclusions/ Recommendations | Limitations |
|---|---|---|---|---|---|---|
| US Government Accountability Office. Heath Resources and Services Administration: Review of Internal Communication Mechanisms, Staffing, and Use of Contracts. Published Dec. 3, 2013. Accessed Apr. 10, 2023. https://www.gao.gov/assets/gao-14-52.pdf | Government Report | "GAO was asked to review HRSA's management and operations. This report examines (1) HRSA's internal communication mechanisms and how they are used to support the agency's mission; (2) HRSA's staffing and how the agency plans for attrition; and (3) HRSA's use of contracts to support its operations." | HRSA | HRSA's "communication methods include an annual operational planning process for allocating agency resources, workgroups that involve staff from across the agency to work on issues of a cross-cutting nature, and regular meetings between the Office of the Administrator and leaders of the agency's various organizational components." HRSA's staff grew 30 percent between 2008 and 2012 and has plans to handle attrition due to retirement, including leadership programs. In 2012, $240 million in appropriations was allocated by HRSA for contracting with short term staffers and specialists to support operations. | N/A | N/A |

© 2023 Knox RP et al. *JAMA Health Forum.*

| Article Citation | Article Type | Study Objective/Article Thesis | Stakeholders Discussed | Results/Analyses | Conclusions/ Recommendations | Limitations |
|---|---|---|---|---|---|---|
| US Government Accountability Office. Drug Pricing: Manufacturer Discounts in the 340B Program Offer Benefits, but Federal Oversight Needs Improvement. Published Sept. 23, 2011. Accessed Apr. 10, 2023. https://www.gao.gov/products/gao-11-836 | Government Report | "The Patient Protection and Affordable Care Act (PPACA) mandated that GAO address questions related to the 340B program. GAO examined: (1) the extent to which covered entities generate 340B revenue, factors that affect revenue generation, and how they use the program; (2) how manufacturers' distribution of drugs at 340B prices affects covered entities' or non-340B providers' access to drugs; and (3) HRSA's oversight of the 340B program." | Hospitals, clinics, manufacturers, pharmacies | Thirteen of 29 covered entities interviewed generated revenue from the 340B Program. Manufacturers distribution of drugs at 340B prices did not affect access for covered entities or non-covered entities according to stakeholders interviewed. HRSA's oversight was inadequate to identify diversion and overcharging. | "To ensure appropriate use of the 340B program, GAO recommends that HRSA take steps to strengthen oversight regarding program participation and compliance with program requirements." | N/A |
| US Government Accountability Office. Ryan White Care Act: Effects of Certain Funding Provisions on Grant Awards. Published Sept. 18, 2009. Accessed Apr. 10, 2023. https://www.gao.gov/assets/gao-09-894.pdf | Government Report | "GAO was asked to examine CARE Act funding provisions. This report provides information on (1) how many Part B grantees collect and use name-based HIV case counts for CARE Act funding; (2) the distribution of Part A hold- harmless funding; and (3) reductions in Part B grantees' funding due to unobligated balance provisions." | Clinics | Forty-seven of 59 part B grantees collected and used name-based HIV case counts for CARE Act funding. Part A hold-harmless funding was distributed in eligible metropolitan areas more in 2009 compared to 2004. Sixteen Part B grantees had reductions in funding due to unobligated balance provisions. | N/A | N/A |

| Article Citation | Article Type | Study Objective/Article Thesis | Stakeholders Discussed | Results/Analyses | Conclusions/ Recommendations | Limitations |
|---|---|---|---|---|---|---|
| US Government Accountability Office. Ryan White Care Act: Improved Oversight Needed to Ensure AIDS Drug Assistance Programs Obtain Best Prices for Drugs. Published Apr. 26, 2006. Accessed Apr. 10, 2023. https://www.gao.gov/assets/gao-06-646.pdf | Government Report | "GAO was asked to examine (1) coverage differences among ADAPs, (2) how the prices ADAPs reported paying for HIV/AIDS drugs compare to 340B prices, (3) how HRSA monitors the drug prices ADAPs pay, and (4) how the 340B prices compare to other selected federal drug pricing programs." | Clinics | ADAP's program design and funding vary and coverage and eligibility vary by state. Some ADAPs reported paying prices above 340B ceiling prices for drugs. HRSA does not routinely monitor if ADAPs are paying 340B prices. Some ADAPs purchasing drugs at 340B prices may more than other federal agencies. | "GAO recommends that HRSA require ADAPs to report the final prices they paid for drug purchases, net of rebates, and that HRSA routinely determine whether these prices paid are at or below the 340B prices. HRSA stated that these steps would be labor intensive and it lacks capacity to carry out such oversight. We believe there are cost-effective processes HRSA could use." | N/A |
| US Government Accountability Office. Medicare: Payment for Blood Clotting Factor Exceeds Providers' Acquisition Cost. Published Jan. 10, 2003. Accessed Apr. 10, 2023. https://www.gao.gov/assets/gao-03-184.pdf | Government Report | "GAO was asked to compare provider costs of purchasing clotting factor with Medicare's payment for it and to identify costs to providers associated with delivering clotting factor." | Clinics | Hemophilia treatment centers commonly purchase clotting factors and 340B discounts which are substantially lower than Medicare payment levels. | "GAO recommends that the Administrator of the Centers for Medicare & Medicaid Services (CMS) establish Medicare payment levels for clotting factor that are more closely related to providers' acquisition costs and then establish a separate payment for the cost of delivering clotting factor to Medicare beneficiaries. The Department of Health and Human Services (HHS) agreed with our recommendations." | N/A |

© 2023 Knox RP et al. *JAMA Health Forum.*

| Article Citation | Article Type | Study Objective/Article Thesis | Stakeholders Discussed | Results/Analyses | Conclusions/ Recommendations | Limitations |
|---|---|---|---|---|---|---|
| US Government Accountability Office. HIV/AIDS Drugs: Funding Implications of New Combination Therapies for Federal and State Programs. Published Oct. 14, 1998. Accessed Apr. 10, 2023. https://www.gao.gov/assets/hehs-99-2.pdf | Government Report | GAO was asked to “describe (1) federal and state spending on HIV and AIDS drug treatment, by major programs, over the last several years; (2) the estimated number of people with AIDS and HIV on combination drug therapy who are covered by Medicaid or other publicly funded programs, and measures that have been taken to stretch the resources in the CARE Act programs; and (3) the potential impacts of new drug therapies on federal and state government outlays.” | Clinics | The federal government paid for more than half of the cost of HIV/AIDS care in the US. Demand for care and expenditures on HIV/AIDS drugs were expected to increase related to combination therapies. ADAPs take various approaches in providing combination therapies. Some ADAPs are able to purchase HIV/AIDS drugs at discounts through the 340B Program. | N/A | N/A |
| **Google** | | | | | | |
| 340B Community Health Program. UCLA Health. Accessed Apr. 10, 2023. https://www.uclahealth.org/discover-ucla-health/about/office-community/340b-community-health-program | Hospital Webpage | To describe the hospital’s experience with the 340B Program | Hospital, patients | "- $246.3 million: Amount provided as charity care, in-kind health services, volunteer time, health education training and cost of uncompensated care for Medi-Cal patients (FY 20).<br>- $373.3 million: Cost of uncompensated care for Medicare patients (FY 20)."<br>"$75.2 million: Amount of savings realized from the 340B program in Fiscal Year 2020." | "Scaling back the 340B Program would threaten UCLA Health's capacity to offer the services described above to our patients." | N/A |

© 2023 Knox RP et al. *JAMA Health Forum.*

| Article Citation | Article Type | Study Objective/Article Thesis | Stakeholders Discussed | Results/Analyses | Conclusions/ Recommendations | Limitations |
|---|---|---|---|---|---|---|
| 340B Drug Discount Program. Beaumont. Accessed Apr. 10, 2023. https://www.beaumont.org/community/340b-drug-discount-program | Hospital Webpage | To describe the hospital's experience with the 340B Program | Hospital, patients | * "Beaumont's 340B savings are part of our community benefit contribution totaling nearly $190 million in 2017* and these dollars support the following types of health care and social service programs within communities served by Beaumont." The hospital uses these savings for school clinics, health education, diabetes prevention and management, access to healthy foods, free discharge prescriptions, among other services. | N/A | N/A |
| 340B Drug Discount Program. Maury Regional Health. Accessed Apr. 10, 2023. https://www.mauryregional.com/our-services/pharmacy-services/340b-drug-discount-program | Hospital Webpage | To describe the hospital's experience with the 340B Program | Hospital, patients | "Below is a partial summary of Maury Regional Health's outreach to the community, in part as a result of the 340B program:<br><br>Cost of charity care and bad debt: $13,851,586<br>Free health care for Maury County Jail inmates: $502,948<br>Support for Maury County school athletic trainers: $205,270<br>Additional community services: $411,137" | N/A | N/A |

© 2023 Knox RP et al. *JAMA Health Forum.*

| Article Citation | Article Type | Study Objective/Article Thesis | Stakeholders Discussed | Results/Analyses | Conclusions/ Recommendations | Limitations |
|---|---|---|---|---|---|---|
| 340B Drug Discount Program. UVA Health. Accessed Apr. 10, 2023. https://uvahealth.com/services/pharmacy/discount-program | Hospital Webpage | To describe the hospital's experience with the 340B Program | Hospital, patients | "This program is a critical component in advancing UVA Health's mission to serve our community's most vulnerable. By providing generic medications at a significant price reduction, we're able to increase access to life-saving drugs; many cost only $4 per month.<br><br>In 2018, UVA Health saved $88 million due to the 340B Program's discounts from drug manufacturers. Such reduced drug expenditures allow UVA to instead reinvest in programs to ensure economically disadvantaged patients have access to specialized care, including in locations closer to home." | N/A | N/A |
| 340B Drug Pricing Program - Saint Joseph Mount Sterling. CHI - Saint Joseph Health. Accessed Apr. 10, 2023. https://www.chisaintjosephhealth.org/chi-saint-joseph-health/hospitals-locations/saint-joseph-mount-sterling/about-us/340b-hospital-commitment | Hospital Webpage | To describe the hospital's experience with the 340B Program | Hospital, patients | "Benefits of the 340B Program<br><br>The Outpatient Infusion Center which provides comprehensive cancer care, chemotherapy and non-oncology infusion services in the patient's home community.<br>Uncompensated care provided to improve the health of our community.<br>340B Savings: $2,400,000<br>Approximate DHS percentage: 12%" | "Impact if the program is scaled back<br><br>Saint Joseph Mount Sterling would be required to scale back local infusion services as well other ambulatory services.<br>Saint Joseph Mount Sterling provides a plethora of community health improvement services which would be cut without 340B resources." | N/A |

© 2023 Knox RP et al. *JAMA Health Forum.*

| Article Citation | Article Type | Study Objective/Article Thesis | Stakeholders Discussed | Results/Analyses | Conclusions/ Recommendations | Limitations |
|---|---|---|---|---|---|---|
| 340B Drug Pricing Program jeopardized by Big Pharma. Community Healthcare System NE Kansas. Published Oct. 21, 2020. Accessed Apr. 10, 2023. https://www.chcsks.org/blog/post/340b-drug-pricing-program-jeopardized-by-big-pharma- | Hospital Webpage | To describe the hospital's experience with the 340B Program | Hospital, patients | "In fiscal year 2020, CHCS received $1.46 million in 340B support and spent $1.09 million on pharmaceuticals. CHCS assumed $1.19 million in uncompensated care and bad debt. Savings from the 340B program have helped financially support the following:<br><br>Estimated annual patient savings on prescription drugs of more than $900,000;<br>Full-time medical services in our communities (Onaga, Holton, Frankfort, Westmoreland, Centralia, and St. Marys);<br>Relationships with local pharmacies in Seneca, Holton, Onaga, St. Marys, Blue Rapids, Topeka, and Marysville;<br>Stop the Bleed training in rural northeast Kansas communities;<br>Mental Health First Aid scholarships for community members to help them learn how to assist those experiencing mental health crises; and<br>A smoking cessation program." | "Unfortunately, some major drug manufacturers have unilaterally stopped providing required 340B discounts when drugs are dispensed at community pharmacies or are asking for access to patient insurance claims and placing new reporting demands on hospitals. The companies are seeking to eliminate 340B discounts even as they continue to offer rebates to large insurance companies. All of this is being done in an effort to cripple the program for rural pharmacies and hospitals." | N/A |

© 2023 Knox RP et al. *JAMA Health Forum.*

| Article Citation | Article Type | Study Objective/Article Thesis | Stakeholders Discussed | Results/Analyses | Conclusions/ Recommendations | Limitations |
|---|---|---|---|---|---|---|
| 340B Drug Pricing Program update. Norton Rose Fulbright. Published Dec. 2020. Accessed Apr. 10, 2023. https://www.nortonrosefulbright.com/en-ca/knowledge/publications/4b511b9a/340b-drug-pricing-program-update | Commentary | To discuss certain recent developments in the 340B Program | Manufacturers, federal government | “Certain drug companies have informed hospitals that the companies will no longer provide 340B Program pricing for drugs dispensed through contract pharmacies… The pharmaceutical companies cite to duplicate discounts related to contract pharmacies as necessitating their actions. The American Hospital Association has requested HHS to direct pharmaceutical manufacturers to cease charging hospitals and other covered entities more than the 340B Program price for drugs dispensed by a contract pharmacy."<br>* "Congress in the 2010 Patient Protection and Affordable Care Act provided for an administrative dispute resolution (ADR) process for health care providers to take action against pharmaceutical companies for violations of the 340B Program statutory requirements." | N/A | N/A |

© 2023 Knox RP et al. *JAMA Health Forum.*

| Article Citation | Article Type | Study Objective/Article Thesis | Stakeholders Discussed | Results/Analyses | Conclusions/ Recommendations | Limitations |
|---|---|---|---|---|---|---|
| 340B Drug Pricing Program. Cascade Medical. Accessed Apr. 10, 2023. https://cascademedical.org/340b-drug-pricing-program | Hospital Webpage | To describe the hospital's experience with the 340B Program | Hospital, patients | The hospital used the 340B Program to:<br><br>"Reduce[] prices allowed Cascade Medical to save $30,000 in 2018. After processing fees paid to Safeway and the administrator, Cascade Medical expects to save $160,000 in 2019.<br><br>Cascade Medical uses its 340B cost savings to purchase and maintain equipment needed for patient care, treatment, diagnostics, safety or facility needs." | N/A | N/A |

© 2023 Knox RP et al. *JAMA Health Forum.*

| Article Citation | Article Type | Study Objective/Article Thesis | Stakeholders Discussed | Results/Analyses | Conclusions/ Recommendations | Limitations |
|---|---|---|---|---|---|---|
| 340B Drug Pricing Program. Labette Health. Accessed Apr. 10, 2023. https://www.labettehealth.com/about-us/340b-drug-pricing-program/ | Hospital Webpage | To describe the hospital's experience with the 340B Program | Hospital, patients | * "Some examples of how 340B works in the Labette Health community include treating uninsured patients, the unreimbursed cost of treating Medicaid and Medicare patients, community health improvement services, improving quality care through the reduction of readmissions, and expanding access to care in underserved areas. Labette Health has opened clinics, express care centers, and Hospital Outpatient Departments in areas where hospital closures have created a lack of healthcare services."<br>* "Labette Health uses a portion of their 340B savings to care for the nutritional aspects of community health…"<br>* "operates the Rector Diabetes Education and Resource Center with a portion of our 340B savings…" | N/A | N/A |

| Article Citation | Article Type | Study Objective/Article Thesis | Stakeholders Discussed | Results/Analyses | Conclusions/ Recommendations | Limitations |
|---|---|---|---|---|---|---|
| 340B Drug Pricing Program. Marshall Medical Center. Accessed Apr. 10, 2023. https://www.marshallmedical.org/about-us/340b-drug-pricing-program/ | Hospital Webpage | To describe the hospital's experience with the 340B Program | Hospital, patients | "*$84 million - our uncompensated care includes charity care, bad debt and underpayment<br>* 150,000 - residents of rural and semi rural El Dorado County in our service area<br>* $6.2 million - our approximate2019 340B savings<br>* 1,700 - employees serving the needs of our community in 22 El Dorado County sites<br>* 4 - average number of chronic diseases a patient in the Community Care Network has<br>* 77% - our population enrolled in Medicare or MediCal" | N/A | N/A |

© 2023 Knox RP et al. *JAMA Health Forum.*

| Article Citation | Article Type | Study Objective/Article Thesis | Stakeholders Discussed | Results/Analyses | Conclusions/ Recommendations | Limitations |
|---|---|---|---|---|---|---|
| 340B Drug Pricing Program. Minneapolis. Accessed Apr. 10, 2023. https://lims.minneapolismn.gov/RCA/7314 | Hospital Webpage | To describe the hospital's experience with the 340B Program | Hospital, patients | "In 2019, Abbott Northwestern Hospital save over $2.4 million in drug costs through discounted 340B drug prices. The savings allows Abbott Northwestern Hospital to provide an expanded range of health services to Minneapolis' vulnerable patients and communities.<br><br>The multidisciplinary Allina Health 340B Compliance Committee has committed to use 340B program savings to prevent opioid overdoses in Allina Health's twelve emergency departments by providing each with Naloxone kits. Additional work continues to be done to draw a direct connection of how Allina Health uses 340B savings to add new services in support of patients." | N/A | N/A |

© 2023 Knox RP et al. *JAMA Health Forum.*

| Article Citation | Article Type | Study Objective/Article Thesis | Stakeholders Discussed | Results/Analyses | Conclusions/ Recommendations | Limitations |
|---|---|---|---|---|---|---|
| 340B Drug Pricing Program. National Pharmaceutical Council. Accessed Apr. 10, 2023. https://www.npcnow.org/topics/health-spending/340b-drug-pricing-program | Report | To review criticisms of the 340B Program | Hospitals, clinics, manufacturers, pharmacies | "Despite the intended purpose of 340B pricing, there is mixed evidence on how covered entities are using 340B revenues to benefit patients and whether the program lowers healthcare costs for individuals. Researchers have found that 340B-participating entities often do not pass manufacturer discounts directly to patients. 340B hospitals charge cash-paying or uninsured patients a median of 3.8 times more than they pay to purchase 340B drugs – the same margins they make on 340B drugs from commercial insurers. Program rules may allow safety net providers to use discounted 340B pricing on medicines to cover other underfunded or uncompensated patient care, making it difficult for decision-makers and policy-makers to identify health system challenges that need to be addressed. Despite this potential to divert medication savings from the 340B program to other areas of care, large hospital systems, certain pharmacies, and other middlemen have not been shown to provide more charity care compared to facilities that do not participate in 340B." | "Thorough review and revision of program requirements, implementation, and administration may help ensure 340B benefits are flowing to underserved patients as intended. Proper oversight can help discounted 340B pricing continue as a vital part of the U.S. health care safety net." | N/A |

© 2023 Knox RP et al. *JAMA Health Forum.*

| Article Citation | Article Type | Study Objective/Article Thesis | Stakeholders Discussed | Results/Analyses | Conclusions/ Recommendations | Limitations |
|---|---|---|---|---|---|---|
| 340B Drug Pricing Program. Scripps. Accessed Apr. 10, 2023. https://www.scripps.org/locations/hospitals/scripps-mercy-hospital/340b-drug-pricing-program | Hospital Webpage | To describe the hospital's experience with the 340B Program | Hospital, patients | "Scripps Mercy Hospital contributed $70,288,342 to community benefits for Fiscal Year 2020, including:<br>$10,204,940 in charity care<br>$12,297,580 in Medi-Cal and other means-tested government programs<br>$26,192,822 in Medicare shortfall<br>$3,252,959 in bad debt<br>$3,658,208 in subsidized health services"<br><br>"One way Scripps Mercy is able to provide care to some of our most needy patients is through in-lieu of funds. In-lieu of funds are used for unfunded or underfunded patients and their post-discharge needs. Funds are used for board and care, skilled nursing facilities, long-term acute care and home health. In addition, funds are also used for medications, equipment and transportation services. $1,036,031 was spent on in-lieu of funds in 2020." | N/A | N/A |

© 2023 Knox RP et al. *JAMA Health Forum.*

| Article Citation | Article Type | Study Objective/Article Thesis | Stakeholders Discussed | Results/Analyses | Conclusions/ Recommendations | Limitations |
|---|---|---|---|---|---|---|
| 340B Drug Pricing Program. Upstate University Hospital. Accessed Apr. 10, 2023. https://www.upstate.edu/outpatient-pharmacy/programs/340b.php | Hospital Webpage | To describe the hospital's experience with the 340B Program | Hospital, patients | "Savings from participation in the 340B program have fueled pharmacy-lead community benefit initiatives such as:<br><br>Medication Assistance Program<br>Meds-To-Beds services<br>Prior Authorization services<br>In-house Retail/Specialty Pharmacy Program<br>These services aim to provide easy access to medications, affordability and increased therapy adherence for our most vulnerable patients." | N/A | N/A |

| Article Citation | Article Type | Study Objective/Article Thesis | Stakeholders Discussed | Results/Analyses | Conclusions/ Recommendations | Limitations |
|---|---|---|---|---|---|---|
| 340B Drug Pricing Program. VCU Health. Accessed Apr. 10, 2023. https://www.vcuhealth.org/locations/vcu-medical-center/patient-guide/pharmacy-services/340b-drug-pricing-program | Hospital Webpage | To describe the hospital's experience with the 340B Program | Hospital, patients | * "Discounted Medications - Qualifying patients get discharge medications at no cost and may receive refills for $4 at our pharmacies. Nearly 2,100 patients received 27,300 discounted or free medications in 2020. With 7 pharmacies, we're able to reach our patients who need this the most. * Medical-Legal Partnership - Some circumstances make health problems more difficult to manage. Qualifying patients and families can get free legal advice and representation with our Medical-Legal Partnership (MLP) Program. MLP helps patients access and maintain housing, employment, public benefits and more, so you can focus on your health. * Virginia Supportive Housing Partnership - Our medically complex patients, like those living with sickle cell disease, benefit from our partnership with Virginia Supportive Housing. The partnership provides permanent housing and supportive services for those who need it most." | N/A | N/A |

© 2023 Knox RP et al. *JAMA Health Forum.*

| Article Citation | Article Type | Study Objective/Article Thesis | Stakeholders Discussed | Results/Analyses | Conclusions/ Recommendations | Limitations |
|---|---|---|---|---|---|---|
| 340B Helps the Most Vulnerable Patients. American Association of Medical Colleges. Published Mar. 3, 2019. Accessed Apr. 10, 2023. https://www.aamc.org/news-insights/340b-helps-most-vulnerable-patients | Report | To explain how the 340B program benefits teaching hospitals, patients, and communities. | Hospitals, patients | "Across the country, savings from the 340B program fund a huge range of supports: smoking cessation, drug treatment, transportation to appointments, mobile clinics with bilingual providers, access to healthy food, and more." *, "The 340B program is one of the most effective health care programs. It represents less than 3% of total U.S. drug sales. There is no cost to taxpayers, and it allows hospitals to provide lifesaving programs to their most vulnerable patients and communities," * "340B supports another key local program, created to address how frequently patients failed to fill prescriptions or take them correctly. For the past five years, pharmacists have been visiting the homes of discharged patients to check for expired drugs, possible medication interactions, and misunderstood instructions. [...] The program has produced measurable results: participants' hospital readmission rate was 8% compared with 17% for nonparticipants, according to a 2014 study. " | N/A | N/A |

© 2023 Knox RP et al. *JAMA Health Forum.*

| Article Citation | Article Type | Study Objective/Article Thesis | Stakeholders Discussed | Results/Analyses | Conclusions/ Recommendations | Limitations |
|---|---|---|---|---|---|---|
| 340B Program at UnityPoint Health. UnityPoint Health. Accessed Apr. 10, 2023. https://govaffairs.unitypoint.org/wp-content/uploads/340B-Flyer-2021-FINAL.pdf | Hospital Webpage | To describe the hospital's experience with the 340B Program | Hospital, patients | "In 2019, the 340B Drug Discount Program resulted in significant savings for the people and communities served by UnityPoint Health:<br>• $90million in 340B program savings at UnityPoint Health<br>• $237.6 million in total Uncompensated Care<br>• $363.6 million in total Drug Spend without 340B program discounts" | N/A | N/A |

© 2023 Knox RP et al. *JAMA Health Forum.*

| Article Citation | Article Type | Study Objective/Article Thesis | Stakeholders Discussed | Results/Analyses | Conclusions/ Recommendations | Limitations |
|---|---|---|---|---|---|---|
| 340B Program in Action. Einstein Healthcare Network. Accessed Apr. 10, 2023. https://www.einstein.edu/about/community/340b-program | Hospital Webpage | To describe the hospital's experience with the 340B Program | Hospital, patients | "The 340B program helps Einstein increase access to much needed medications for our uninsured and underinsured patients. We utilize the 340B program to fill all prescriptions for inpatients discharged from the hospital regardless of their ability to pay. This insures that patients have immediate access to medication upon their return home whether or not they have any prescription insurance. We provide an initial 30 day prescription at the time of discharge. 340B savings also help support Einstein's Medication REACH program, which includes:<br>- Reconciliation of their medication lists<br>- Education with a transitions of care pharmacist that is custom to the patient's needs<br>- Resolution of access to care issues<br>- Counseling post-discharge resulting in healthier patients at home" | N/A | N/A |

© 2023 Knox RP et al. *JAMA Health Forum.*

| Article Citation | Article Type | Study Objective/Article Thesis | Stakeholders Discussed | Results/Analyses | Conclusions/ Recommendations | Limitations |
|---|---|---|---|---|---|---|
| 340B Program. Archbold. Accessed Apr. 10, 2023. https://archbold.org/340b-program | Hospital Webpage | To describe the hospital's experience with the 340B Program | Hospital, patients | "$11 million—approximate annual 340B savings that benefit Archbold patients in rural South Georgia."<br><br>"• $101.26 million—cost of uncompensated care Archbold provided uninsured and underinsured patients, regardless of their ability to pay."<br>* "Archbold's participation in the 340B program allows us to stretch limited federal resources to reduce the price of outpatient pharmaceuticals for patients, while also expanding health services to patients in the rural communities we serve. Archbold uses the savings from the 340B Program to offset the expense of healthcare services and medications at discharge for uninsured and underinsured patients. | N/A | N/A |

© 2023 Knox RP et al. *JAMA Health Forum.*

| Article Citation | Article Type | Study Objective/Article Thesis | Stakeholders Discussed | Results/Analyses | Conclusions/ Recommendations | Limitations |
|---|---|---|---|---|---|---|
| 340B Program. Indiana University Health. Published Mar. 2019. Accessed Apr. 10, 2023. https://cdn.iuhealth.org/global/340B-Program_iu-health.pdf?mtime=20191220122333&focal=none | Hospital Webpage | To describe the hospital's experience with the 340B Program | Hospital, patients | "With the 340B drug discounts, IU Health is able to provide high quality care to all our patients, regardless of income level or insurance coverage. Reduced drug costs have enabled IU Health to provide much needed healthcare services to Indiana's most vulnerable Hoosiers. Such services include:<br>• Adult and Pediatric Pulmonary care<br>• Developmental Pediatrics<br>• HIV Care<br>• Pediatric and Adult Behavioral Health Liaison service<br>• Riley Burn Unit" | "340B program as an essential part of the healthcare safety net—that does not rely on taxpayer dollars— for low-income patients and rural communities. Recently there have been efforts to scale back the 340B program which would reduce the financial assistance provided to IU Health. This would significantly jeopardize our ability to continue offer much needed services to our patients and communities." | N/A |

© 2023 Knox RP et al. *JAMA Health Forum.*

| Article Citation | Article Type | Study Objective/Article Thesis | Stakeholders Discussed | Results/Analyses | Conclusions/ Recommendations | Limitations |
|---|---|---|---|---|---|---|
| 340B Program. Oroville Hospital. Accessed Apr. 10, 2023. https://www.orovillehospital.com/patients/340bprogram | Hospital Webpage | To describe the hospital's experience with the 340B Program | Hospital, patients | * "In 2018, Oroville Hospital provided about 11 million dollars in charity care and roughly saved 50% on overall medication costs due to the 340B program." * "One key program funded by 340B saving is our Hospital Discharge medication program. This program provides vital medications for patients being discharged from Oroville Hospital that are uninsured or underinsured. …" * "Another service that benefits from the savings of 340B drug pricing program is our infusion and cancer center …. " *"We do biannual health fairs where the uninsured can receive free educational information, free health care advice, free health screenings such as COPD screening, blood pressure monitoring, oxygen saturation checks, cholesterol checks, pulse checks and blood glucose checks and free flu shots." | N/A | N/A |

© 2023 Knox RP et al. *JAMA Health Forum.*

| Article Citation | Article Type | Study Objective/Article Thesis | Stakeholders Discussed | Results/Analyses | Conclusions/ Recommendations | Limitations |
|---|---|---|---|---|---|---|
| 340B Program. UCI Health. Accessed Apr. 10, 2023. https://www.ucihealth.org/340b-program | Hospital Webpage | To describe the hospital's experience with the 340B Program | Hospital, patients | * "In fiscal 2021, it provided $77.4 million in charity care and about $95.1 million in uncompensated care, largely to Medi-Cal and uninsured patients in accordance with the organization's financial assistance policy. Since there is no county hospital, about 75% of UCI Health patients are insured by government payers. California has one of the lowest Medicaid provider reimbursement rates in the country, and the prices of specialty services are much higher than the reimbursement received."<br>* "-Colonoscopies that employ artificial intelligence<br>- Increased access to care for the medically underserved and improved community health through preventive practices" | N/A | N/A |

© 2023 Knox RP et al. *JAMA Health Forum.*

| Article Citation | Article Type | Study Objective/Article Thesis | Stakeholders Discussed | Results/Analyses | Conclusions/ Recommendations | Limitations |
|---|---|---|---|---|---|---|
| 340B Program. Valley Presbyterian Hospital. Accessed Apr. 10, 2023. https://www.valleypres.org/community/community-benefit/340b-program/ | Hospital Webpage | To describe the hospital's experience with the 340B Program | Hospital, patients | "- Access to specialty care not otherwise available to many low-income individuals in the community<br>- Clinical pharmacy services, such as disease management programs<br>- Mental health services<br>- Transportation to needy patients allowing them access to essential health care services<br>- Free vaccinations and health screening for vulnerable populations<br>- New community outreach programs" | "Scaling back the 340B Program would jeopardize VPH's capacity to offer all the services mentioned above to our patients." | N/A |

© 2023 Knox RP et al. *JAMA Health Forum.*

| Article Citation | Article Type | Study Objective/Article Thesis | Stakeholders Discussed | Results/Analyses | Conclusions/ Recommendations | Limitations |
|---|---|---|---|---|---|---|
| Analysis of the Governor's 340B Medi-Cal Proposal. Legislative Analyst's Office. Published Mar. 21, 2018. Accessed Apr. 10, 2023. https://lao.ca.gov/Publications/Report/3790 | Report | To analyze the Governor's 2018-19 budget proposal to eliminate the use of the 340B Drug Pricing Program in Medi-Cal | Hospitals, clinics, federal government, state governments | "The Governor's proposal requires the use of the federal Medicaid discount program and prohibits the use of the 340B Program for a given drug dispensed to a Medi-Cal enrollee. In support of his proposal, the Governor cites challenges in administering the federal Medicaid discount program in conjunction with the 340B Program (preventing prohibited duplicate discounts after the fact) and asserts that the proposal would result in state General Fund savings. The administration does not currently have, but is working on, an estimate of the savings that would ultimately be generated under its proposal." "We estimate that there are at least 1,500 covered entity sites in California that serve as Medi-Cal providers and dispense 340B prescription drugs to Medi-Cal patients… since the implementation of the ACA, the number of covered entity sites participating in Medi-Cal has increased dramatically. " | N/A | N/A |

© 2023 Knox RP et al. *JAMA Health Forum.*

| Article Citation | Article Type | Study Objective/Article Thesis | Stakeholders Discussed | Results/Analyses | Conclusions/ Recommendations | Limitations |
|---|---|---|---|---|---|---|
| Arwich N. Vermont & the 340B Drug Pricing Program. The University of Vermont Health Network. Accessed Apr. 10, 2023. https://gmcboard.vermont.gov/sites/gmcb/files/documents/340B%20Intro%20for%20GMCB%20Technical%20Advisory%20Group.pdf | Presentation | To discuss the impact of the 340B Program in Vermont | Hospitals, clinics, patients | "• Hospitals and other providers depend on 340B to preserve patient access to critical health care services<br>• Some hospitals use 340B dollars to provide direct aid through patient assistance programs<br>• For the UVM Health Network's Vermont hospitals in FY2019, the savings amount was over $100 million<br>• The Health Network's actual margin for Vermont in FY2019 was $41 million (2.5%)<br>• Without 340B, health care in Vermont would look very different and cost Vermonters a great deal more" | N/A | N/A |
| Bailey V. Are PBMs Unjustly Profiting from 340B Drug Pricing Program?. RevCycle Intelligence. Published June 2, 2022. Accessed Apr. 10, 2023. https://revcycleintelligence.com/news/are-pbms-unjustly-profiting-from-340b-drug-pricing-program | News | To review a brief submitted by the Community Oncology Alliance criticizing pharmacy benefit managers' financial benefits from the 340B Program | Hospitals, clinics, pharmacy benefit managers | "COA argued that a lack of oversight from the Health Resources and Services Administration (HRSA) on the use of contract pharmacies has allowed PBMs to exploit 340B savings.<br><br>In addition, the brief alleged that the profit opportunities presented by the 340B program have incentivized PBMs to drive out non-affiliated pharmacies, impacting patient and provider access to discounted drugs." | N/A | N/A |

© 2023 Knox RP et al. *JAMA Health Forum.*

| Article Citation | Article Type | Study Objective/Article Thesis | Stakeholders Discussed | Results/Analyses | Conclusions/ Recommendations | Limitations |
|---|---|---|---|---|---|---|
| Becker C. State Options for Managing the 340B Drug Pricing Program. National Conference of State Legislatures. Published July 18, 2022. Accessed Apr. 10, 2023. https://www.ncsl.org/health/state-options-for-managing-the-340b-drug-pricing-program | Report | To discuss state actions related to the 340B Program | Hospitals, clinics, manufacturers, pharmacies, patients, pharmacy benefit managers | "at least 38 states use the Medicaid Exclusion File to avoid duplicate discounts. At least 10 states prohibit pharmacy benefit managers (PBMs) and other payers from denying 340B pricing to CEs or a contract pharmacy acting on their behalf. Another strategy lawmakers may consider is to enforce 340B billing by monitoring provider network contracts…. States may also leverage 340B discounts for state departments of correction by entering into agreements with 340B providers to administer certain high-cost medications (e.g. hepatitis C or HIV anti-virals) to their inmate populations." | N/A | N/A |

© 2023 Knox RP et al. *JAMA Health Forum.*

| Article Citation | Article Type | Study Objective/Article Thesis | Stakeholders Discussed | Results/Analyses | Conclusions/ Recommendations | Limitations |
|---|---|---|---|---|---|---|
| Berberabe T. Oncology Drug Debate Heats Up Over 340B Discount Program. OncoLive. Published Nov. 22, 2013. Accessed Apr. 10, 2023. https://www.onclive.com/view/oncology-drug-debate-heats-up-over-340b-discount-program | Commentary | To discuss the debates related to the 340B Program and oncology practices | Hospitals, clinics | "Oncology drugs are coming under scrutiny because there typically is a higher spread or margin involved in their use. It's estimated that a third of US hospitals participate in the 340B program. Annual drug purchases under the program are projected to double—from $6 billion in 2010 to $12 billion in 2016. Drug discounts range from 20% to 50%. The profits for eligible hospital are enormous." * "Not surprisingly, the American Hospital Association is opposed to any efforts to scale back the 340B program, arguing that the program "is essential to helping safety-net providers stretch limited resources to better serve their communities."" | N/A | N/A |

© 2023 Knox RP et al. *JAMA Health Forum.*

| Article Citation | Article Type | Study Objective/Article Thesis | Stakeholders Discussed | Results/Analyses | Conclusions/ Recommendations | Limitations |
|---|---|---|---|---|---|---|
| Blalock E. Measuring the Relative Size of the 340B Program: 2020 Update. Berkeley Research Group. Published June 30, 2022. Accessed Apr. 10, 2023. https://www.thinkbrg.com/insights/publications/measuring-relative-size-340b-program-2020-update/ | Report | To measure the size of the 340B Program compared to other federal programs paying for prescription drugs | Hospitals, clinics, payers | "the 340B program is larger than all other federal drug programs considered, except for Medicare Part D. Despite the size of the 340B program, HRSA OPA had a budget of only $10 million in fiscal year 2020,15 compared to the $733 million budgeted for federal administration at CMS. Additionally, the data reported by HRSA OPA on the 340B program is significantly more limited than what is reported for other programs. …" | "The 340B program is now the second largest pharmaceutical program under the purview of a federal agency. As the 340B program has grown, it has come to represent an increasingly large share of branded outpatient drug sales. Nevertheless, comprehensive data on the program remains sparse and program guidance remains vague. This growth in 340B has had unintended consequences and may contribute to shifts in the site of care, which can increase costs to both payers and patients. Additionally, with no available data on how covered entities use the margin earned from 340B drugs (the difference between reimbursement and the discounted 340B price), the overall benefit of the program to patients is unclear." | N/A |

© 2023 Knox RP et al. *JAMA Health Forum.*

| Article Citation | Article Type | Study Objective/Article Thesis | Stakeholders Discussed | Results/Analyses | Conclusions/ Recommendations | Limitations |
|---|---|---|---|---|---|---|
| Cottage Health's 340B Program. Cottage Health. Accessed Apr. 10, 2023. https://www.cottagehealth.org/about/340b-program/ | Hospital Webpage | To describe the hospital's experience with the 340B Program | Hospital, patients | "The 340B program allows Cottage Health to better serve its community of patients, regardless of income level or insurance coverage. Cost savings from reduced 340B drug pricing has been invested back into hospital programs and services since the program's inception. This includes, but is not limited to, the following programs:<br><br>Medications upon discharge at low or no cost to patients with financial need<br>Patient access to financial assistance<br>Free and low-cost health screenings<br>Level I Trauma Center<br>Level II Pediatric Trauma Center<br>Pediatric multi-specialty clinics<br>Population health program initiatives to improve the overall health and wellness of our community<br>Health professions education" | N/A | N/A |

© 2023 Knox RP et al. *JAMA Health Forum.*

| Article Citation | Article Type | Study Objective/Article Thesis | Stakeholders Discussed | Results/Analyses | Conclusions/ Recommendations | Limitations |
|---|---|---|---|---|---|---|
| D.C. Circuit Upholds Prior Medicare Cuts to 340B Hospitals' Drug Payments and CMS Furthers the Cuts. Ropes & Gray. Published Aug. 5, 2020. Accessed Apr. 10, 2023. https://www.ropesgray.com/en/newsroom/alerts/2020/08/DC-Circuit-Upholds-Prior-Medicare-Cuts-to-340B-Hospitals-Drug-Payments-and-CMS-Furthers-the-Cuts | Commentary | To discuss the implications of the D.C. Circuit's opinion in *AHA v. Azar* | Hospitals, clinics, federal government | "The 2021 proposal (for OOPS reimbursement rates for CY2021) does not address the effect on SCODs reimbursement rates for 340B drugs furnished in off-campus provider- based departments paid under the Medicare physician fee schedule rather than the OPPS. In any event, it appears that SCODs reimbursement cuts for 340B Hospitals, both on- and off-campus, could be here to stay unless challenged further for later years." | N/A | N/A |

© 2023 Knox RP et al. *JAMA Health Forum.*

| Article Citation | Article Type | Study Objective/Article Thesis | Stakeholders Discussed | Results/Analyses | Conclusions/ Recommendations | Limitations |
|---|---|---|---|---|---|---|
| Davis R, French M, Shafer BM. 340B Program Compliance in a Shifting Landscape: What Challenges Do Stakeholders Face?. Quarles. Published May 24, 2022. Accessed Apr. 10, 2023. https://www.quarles.com/newsroom/publications/340b-program-compliance-in-a-shifting-landscape-what-challenges-do-stakeholders-face | Commentary | To update 340B stakeholders on contract pharmacies litigation and other changes to the 340B program | Hospitals, clinics, manufacturers, pharmacies | "If HRSA is not enjoined from taking adverse action pursuant to the May 17, 2021 letters, it could force manufacturers to begin complying and also serve as a strong basis for the court to rule in HRSA's favor in the lawsuit launched by Lilly. HRSA could then use that ruling and seek to apply it to the other lawsuits it faces.<br><br>Further complicating all of this is the fact that on March 16, 2021, a federal judge issued a preliminary injunction blocking HHS from implementing its 340B administrative dispute resolution final rule (ADR Final Rule). As this ADR Final Rule effectively gives HRSA OPA a meaningful ability to penalize non-compliant manufacturers, its validity will have a substantial impact on HRSA OPA's options in enforcing the position outlined in its letters to manufacturers." | N/A | N/A |

© 2023 Knox RP et al. *JAMA Health Forum.*

| Article Citation | Article Type | Study Objective/Article Thesis | Stakeholders Discussed | Results/Analyses | Conclusions/ Recommendations | Limitations |
|---|---|---|---|---|---|---|
| Drugmakers do not need to provide drugs to unlimited contract pharmacies, US court finds. The PharmaLetter. Published Jan. 31, 2023. Accessed Apr. 10, 2023. https://www.thepharmaletter.com/article/drugmakers-do-not-need-to-provide-drugs-to-unlimited-contract-pharmacies-court-finds | News | To cover the updates in litigation regarding 340B contract pharmacies | Hospitals, clinics, manufacturers, pharmacies | "Pharma companies participating in the 340B drug pricing program in the USA do not have to provide medicines ad nauseam to contract pharmacies.<br><br>So ruled the Court of Appeals for the 3rd Circuit on Monday in a case that has pitted pharma companies Sanofi (Euronext: SAN), AstraZeneca (LSE: AZN) and Novo Nordisk (NOV: N) against the Department of Health and Human Services (HHS)" | N/A | N/A |
| Examining HRSA's Oversight of the 340B Program. 115 Cong. 46. Published Jul. 18, 2017. Accessed Apr. 10, 2023. https://www.govinfo.gov/content/pkg/CHRG-115hhrg26929/html/CHRG-115hhrg26929.htm | Committee Report | To examine challenges in HRSA's oversight of the 340B Program. | Hospitals, clinics, manufacturers, pharmacies, patients, HRSA | Speakers discussed the low compliance rates in the 340B Program, the goals of the 340B Program related to improving care for low-income patients, and the need to reform the 340B Program. | N/A | N/A |

© 2023 Knox RP et al. *JAMA Health Forum.*

| Article Citation | Article Type | Study Objective/Article Thesis | Stakeholders Discussed | Results/Analyses | Conclusions/ Recommendations | Limitations |
|---|---|---|---|---|---|---|
| Fact Sheet: The 340B Drug Pricing Program. American Hospital Association. Accessed Apr. 10, 2023. https://www.aha.org/fact-sheets/fact-sheet-340b-drug-pricing-program | Report | To describe the 340B Program and relevant critiques | Hospitals, manufacturers | • "The 340B program generates valuable savings for eligible hospitals to invest in programs that enhance patient services and access to care. Communities in need could lose access to valuable, life-saving care without the financial support from the 340B program."<br>• "While these newly-eligible hospitals represent 54% of actively participating 340B hospitals, the drugs used by these hospitals account for only a small fraction of drugs sold through the 340B program."<br>• "Drug manufacturers are undermining the program. Some drug manufactures have unilaterally stopped providing discounts to 340B drugs in contract pharmacies, violating the 340B statute." | • "340B hospitals are committed to improving transparency. The AHA is working with its 340B member hospitals on efforts to strengthen the 340B program by increasing transparency in the program and helping 340B hospitals communicate publicly the immense value the program brings to patients and communities, such as through the AHA Good Stewardship Principles."<br><br>• "Additional transparency is needed from drug manufacturers." | N/A |

© 2023 Knox RP et al. *JAMA Health Forum.*

| Article Citation | Article Type | Study Objective/Article Thesis | Stakeholders Discussed | Results/Analyses | Conclusions/ Recommendations | Limitations |
|---|---|---|---|---|---|---|
| Federal 340B Drug Pricing Program. Central Maine Medical Center. Accessed Apr. 10, 2023. https://www.cmhc.org/cmmc/services/pharmacy/federal-340b-drug-pricing-program/ | Hospital Webpage | To describe the hospital's experience with the 340B Program | Hospital, patients | * "Savings generated by the 340B drug program help CMH fill the reimbursement gaps from insurance and also supports CMH's $5.2 Million of free care to needy community members annually." * "Reduced prescription costs: Our affiliated outpatient practice areas provide lower costs for eligible medications at CMMC's retail pharmacy and via mail order." * "Rural Areas: Bridgton and Rumford Hospitals provide full service chemotherapy and specialty infusion enabling patients to receive the care they need closer to home. * Central Maine Comprehensive Cancer Center… our integrated, multi-disciplinary cancer program combines a full spectrum of services and medical specialists to diagnose and treat cancer holistically…. * Diabetes & Endocrinology: Working collaboratively with patients, our team of board-certified endocrinologists, certified diabetes educators, registered dietitians along with other specialists create a personalized care plan for each individual." | N/A | N/A |

© 2023 Knox RP et al. *JAMA Health Forum.*

| Article Citation | Article Type | Study Objective/Article Thesis | Stakeholders Discussed | Results/Analyses | Conclusions/ Recommendations | Limitations |
|---|---|---|---|---|---|---|
| Fein AJ. New HRSA Data: 340B Program Reached $29.9 Billion in 2019; Now Over 8% of Drug Sales. Drug Channels. Published June 9, 2020. Accessed Apr. 10, 2023. https://www.drugchannels.net/2020/06/new-hrsa-data-340b-program-reached-299.html | Report | To provide an update on the size of the 340B Program | Hospitals, clinics, manufacturers | *"Discounted purchases made under the program totaled $29.9 billion in 2019—an increase of 23% from the $24.3 billion in 2018"<br>*"According to IQVIA, manufacturers' net revenues were projected to be $360 billion in 2019. Using net revenues, 340B's share in 2019 was 8.3%, or $29.9 billion ÷ $360 billion. This overall average also hides wide variation. Specialty drugs have a higher-than-average share of sales made at 340B discount prices. For example, Merck recently disclosed that one-third of Keytruda's sales come from 340B covered entities."<br>*"There is also no transparency into how 340B discounts are spent, because hospitals and their lobbyists fight any call for them to disclose or account for how they use their 340B profits. There is compelling evidence that hospitals are double-counting 340B savings against their fundamental legal and statutory community benefit obligations as non-profit organizations." | N/A | N/A |

© 2023 Knox RP et al. *JAMA Health Forum.*

| Article Citation | Article Type | Study Objective/Article Thesis | Stakeholders Discussed | Results/Analyses | Conclusions/ Recommendations | Limitations |
|---|---|---|---|---|---|---|
| Fein AJ. The 340B Program Climbed to $44 Billion in 2021—With Hospitals Grabbing Most of the Money. Drug Channels. Published Aug. 15, 2022. Accessed Apr. 10, 2023. https://www.drugchannels.net/2022/08/the-340b-program-climbed-to-44-billion.html | Commentary | To discuss the growth of the 340B Program | Hospitals, clinics, manufacturers | "Observations: Discounted purchases made under the program totaled at least $43.9 billion in 2021—an increase of 15.6% over the $38.0 billion for 2020. The compound average growth rate (CAGR) of 340B purchases was 23.8% from 2015 through 2021. Over the same period, manufacturers' net brand-name drug sales (excluding COVID-19 vaccines) grew at an average annual rate of less than 4%. According to IQVIA, the wholesale acquisition cost (WAC) list price value of 340B purchases was $93.6 billion in 2021. (source) That equates to about 14% of pharmaceutical manufacturers' total gross sales of brand-name drugs at list prices. (This figure also excludes COVID-19 vaccines.) In 2021, the list-to-340B gap—the difference between purchases at list prices and purchases at 340B discounted prices—grew to $49.7 billion (=$93.6 minus $43.9). That's $7.0 billion higher than the 2020 gap." | "The 340B Drug Pricing Program is now unambiguously the second-largest government pharmaceutical program, based on net drug spending. But unlike such programs as Medicare Part D and Medicaid, 340B lacks a regulatory infrastructure, well-developed administrative controls, and clear legislation to guide the program.<br><br>Congress has just passed the Inflation Reduction Act, which will sharply reduce pharmaceutical manufacturers' revenues. It's long past time for legislators to revisit the out-of-control and unregulated 340B program.<br><br>In the meantime, the programs' defenders may want to cool their rhetoric. A while back, lobbying group 340B Health faulted me for exaggerating the size and growth of the 340B program, because "Allegations of 'explosive 340B growth' are not supported by the numbers." Really? More recently, the group launched a website that states: "Drug companies are cutting hospitals' access to 340B savings…"" | N/A |

© 2023 Knox RP et al. *JAMA Health Forum.*

| Article Citation | Article Type | Study Objective/Article Thesis | Stakeholders Discussed | Results/Analyses | Conclusions/ Recommendations | Limitations |
|---|---|---|---|---|---|---|
| Gal A. Examining Hospital Price Transparency, Drug Profits, & the 340B Program. Community Oncology Alliance. Published Sept. 2021. Accessed Apr. 10, 2023. https://communityoncology.org/wp-content/uploads/2021/09/Moto-COA-340B_Hospital_Markups_Report.pdf | Report | To examine compliance with, and insights from, recent hospital price transparency data, with a particular emphasis on oncology and the 340B Drug Payment Program. | Hospitals | "Analyzing the 123 hospitals, we observed that the majority of them did not provide well-organized and easy-to-read datasets; instead, they seemed to be snapshots from internal billing systems."<br>" We have obtained a total of 52,180 individual negotiated prices, each reflecting a unique combination of hospital-payer-drug. None of the 123 hospitals disclosed prices for all of the 59 oncology drugs we examined, and we presume that each hospital carries a subset. The median hospital has negotiated prices for 23 drugs and, for each drug, we obtained negotiated prices from 55 hospitals."<br>"Essentially all hospitals reported commercial insurance and those represented 85 percent of prices obtained; 65 hospitals reported Medicare Advantage plans (10 percent of price data); Medicaid prices were reported by 30 hospitals and represent three percent of price data; and Medigap/other government prices were reported by 14 hospitals (one percent of data)." | "The analysis suggests that 340B drug discounts are captured by hospitals rather than being passed on. […]."<br>"» The 'spread' between the discounted 340B purchase price and the price charged to insurers or patients in 340B hospitals is 3.8 times the median.<br>» 340B hospitals are not reducing prices they charge insurers or patients when their acquisition prices decline, negating efforts to reduce prices at the manufacturer level.<br>» There is pricing inconsistency between hospitals with some pricing drugs 2.0 times more than the median (i.e., 7.6 times their acquisition price or more) and even within hospitals, charges usually vary dramatically.<br>» 340B hospitals are slow to adopt biosimilars.<br>» Most problematic, 340B hospitals charge cash-paying customers the same as the median price of insurers, i.e., 3.8 times their acquisition costs to patients paying cash. In short, to the extent 340B institutions fulfill their mission of providing lower cost care, we are not seeing it reflected in their drug prices." | "The first and most critical is that we are limited by the quality of the data the hospitals provided. We attempted to ensure the data correctly reflect specific hospital negotiated prices, but presumably errors slipped in given the complexity discussed above. We thus believe the data is best viewed on an aggregate basis, rather than focusing on individual hospital drug plans. We specifically avoided outlier data by using primarily median values. We believe the error rate in our data is low enough for median values to fairly reflect 340B hospital's negotiated drug prices. The second is that this is price data. We do not have the volumes transacted at each price. Thus, to the extent a hospital transacts most of its business at a below-median negotiated price, this would not be reflected in this data. Third, for the dataset to reflect a price point, there must be a negotiated price. To the extent a hospital provides a drug for free to a patient, we have no data point to capture that information." |

© 2023 Knox RP et al. *JAMA Health Forum.*

| Article Citation | Article Type | Study Objective/Article Thesis | Stakeholders Discussed | Results/Analyses | Conclusions/ Recommendations | Limitations |
|---|---|---|---|---|---|---|
| Gillard A, Shelby D, White K. Utilization of the 340B Drug Pricing Program in Rural Practices Policy Paper. National Rural Health Association. Published 2019. Accessed Apr. 10, 2023. https://www.ruralhealth.us/NRHA/media/Emerge_NRHA/Advocacy/Policy%20documents/2019-NRHA-Policy-Paper-Utilization-of-the-340B-Drug-Pricing-Program-in-Rural-Practices.pdf | Report | To describe how hospitals in rural areas use the 340B Program | Hospitals | "Rural residents are less likely than urban residents to have health care coverage through their employer, more likely to be low-income, and oftentimes are unable to afford coverage on their own. For hospitals that serve rural residents, this often means higher rates of uncompensated care compared to urban hospitals… Ninety-five percent of the rural hospitals have been able to maintain or provide more uncompensated care services. The most powerful benefit is that 55 percent of rural hospitals reported that the 340B savings are used to keep the doors of their facility open. … Hospitals and other rural health providers depend on the 340B contract pharmacies to provide support for low-income patients with reduced drug prices. 340B rural contract pharmacies are vital because rural hospitals are less likely to maintain in-house outpatient pharmacies." | N/A | N/A |

© 2023 Knox RP et al. *JAMA Health Forum.*

| Article Citation | Article Type | Study Objective/Article Thesis | Stakeholders Discussed | Results/Analyses | Conclusions/ Recommendations | Limitations |
|---|---|---|---|---|---|---|
| Good Stewardship of the 340B Program. Lompoc Valley Medical Center. Accessed Apr. 10, 2023. https://www.lompocvmc.com/about-us/good-stewardship-of-the-340b-program | Hospital Webpage | To describe the hospital's experience with the 340B Program | Hospital, patients | "$6.1 million: Cost of Lompoc Valley Medical Center's uncompensated care.<br>36.39%: Lompoc Valley Medical Center's Disproportionate Share Hospital (DSH) percentage. DSH hospitals such as Lompoc Valley Medical Center serve a significantly disproportionate number of low-income patients."<br><br>"* Discharge prescriptions at no charge for uninsured and homeless patients<br>* Ensuring continued local access to Hematology-Oncology services<br>* Expanding the availability of local specialty physicians and services for our patients<br>* Medication Reconciliation program to ensure accurate medication histories for all patients seen at the hospital<br>* Clinical pharmacy services to improve medication adherence and outcomes" | N/A | N/A |

© 2023 Knox RP et al. *JAMA Health Forum.*

| Article Citation | Article Type | Study Objective/Article Thesis | Stakeholders Discussed | Results/Analyses | Conclusions/ Recommendations | Limitations |
|---|---|---|---|---|---|---|
| Havens J. Health Care Alert: Top Changes to Watch For in the 340B Drug Discount Program. Vorys. Published July 13, 2018. Accessed Apr. 10, 2023. https://www.vorys.com/publication-i-Health-Care-Alert-i-Top-Changes-to-Watch-For-in-the-340B-Drug-Discount-Program | Commentary | To highlight the top 3 changes to the 340B program potentially relevant to providers | Hospitals, clinics | 1. The definition of a patient of the 340B Program remains unclear.<br>2. Critics have recommended increased reporting requirements for covered entities related to contract pharmacy arrangements.<br>3. Increased scrutiny of compliance with the duplicate discount prohibition is needed, especially under Medicaid managed care. | N/A | N/A |

© 2023 Knox RP et al. *JAMA Health Forum.*

| Article Citation | Article Type | Study Objective/Article Thesis | Stakeholders Discussed | Results/Analyses | Conclusions/ Recommendations | Limitations |
|---|---|---|---|---|---|---|
| Hoffman, AK. Supreme Court Will Determine Whether 340B Hospitals Retain Discounts on Medicare Part B Drugs. Commonwealth Fund. Published Nov. 11, 2021. Accessed Apr. 10, 2023. https://www.commonwealthfund.org/blog/2021/supreme-court-340b-hospitals-discounts-medicare-part-b | Commentary | To discuss the impact of the case before the Supreme Court, *AHA v. Becerra* | Hospitals, clinics, federal government | 1. " the case could be significant if the Court uses it to revisit legal doctrine on when agencies deserve deference in interpreting statutory language, … However, from a policy perspective with respect to drug reimbursement, the case's significance is more limited.<br>2. "Yet, the decision will affect a large number of hospitals and clinics, which is surprising as the 340B program was intended to be limited to safety-net hospitals and those serving disproportionately low-income populations."<br>3. "CMS indicated it plans to continue to pay 340B hospitals average sales price minus 22.5 percent for SCODs, even though the survey data collected on acquisition costs justifies even lower rates. This reimbursement rate reduces Medicare spending and beneficiary cost sharing for these outpatient drugs received at 340B hospitals and clinics. Yet, doing also reduces the funds available to many vulnerable hospitals that provide care to low-income and uninsured populations." | "Regardless of how the Supreme Court decides, the case raises the question of whether there is a better way to subsidize the operations of hospitals serving low-income populations than the ability to retain excess reimbursement rates on discounted outpatient drugs" | N/A |

© 2023 Knox RP et al. *JAMA Health Forum.*

| Article Citation | Article Type | Study Objective/Article Thesis | Stakeholders Discussed | Results/Analyses | Conclusions/ Recommendations | Limitations |
|---|---|---|---|---|---|---|
| Kantarjian H, Chapman R. The 340B Drug Pricing Program: Background, Concerns, and Solutions. The ASCO Post. Published Jan. 25, 2016. Accessed Apr. 10, 2023. https://ascopost.com/issues/january-25-2016/the-340b-drug-pricing-program-background-concerns-and-solutions/ | Commentary | To discuss recent criticisms of the 340B Program | Hospitals, clinics, manufacturers, pharmacies | "A major concern is that 340B hospitals provide discounted drugs to insured patients. … Hospitals are able to stretch their scarce resources because they buy drugs at discounted rates but receive reimbursement from insurers through negotiated rates, thereby accessing savings. Hospitals use the savings to treat low-income patients, offset disproportionate levels of uncompensated care, and support unprofitable public health services that other hospitals do not provide. Other concerns: (1) 340B may have caused the shift in private oncology practices to hospital settings through acquisitions of and mergers with community cancer clinics, with the goal of increasing profits from use of oncology products; (2) the number of 340B hospitals continues to expand, 340B locations (contract pharmacies dispensing 340B drugs) are increasing, and 340B sales may continue to increase; and (3) Medicaid expansion under the Affordable Care Act will allow more hospitals to treat Medicaid patients and qualify for 340B." | "The 340B Drug Pricing Program is a critical program that allows organizations caring for large proportions of vulnerable, poor, underserved, or rural populations to access badly needed health care while remaining solvent. The disproportionate amount of uncompensated care that 340B hospitals provide affirms that they deserve the 340B status and that they use their program savings well. Restricting 340B may help increase the already astounding profits of drug companies, but it will harm millions of Americans.<br><br>The 340B Drug Pricing Program is a medical lifeline that should be maintained, nurtured, and expanded—but certainly not curtailed." | N/A |

| Article Citation | Article Type | Study Objective/Article Thesis | Stakeholders Discussed | Results/Analyses | Conclusions/ Recommendations | Limitations |
|---|---|---|---|---|---|---|
| Kaplan D.A. The 340B Program is at a Crossroads. Managed Healthcare Executive. Published Aug. 24, 2022. Accessed Apr. 10, 2023. https://www.managedhealthcareexecutive.com/view/the-340b-program-is-at-a-crossroads | News | To discuss restrictions imposed by manufacturers on delivery of 340B discounted drugs to contract pharmacies | Hospitals, clinics, manufacturers, pharmacies | "Proponents of retaining the discounts say that the loss is harmful to hospitals' abilities to provide services to individuals with lower incomes and to even keep the health system's doors open. Critics of the program, most of whom are drug manufacturers, say the program has grown too large, with for-profit pharmacies and health systems reaping profits and not sharing evidence of whether the funds are used to help the intended patients." | "The swarm of controversy and litigation surrounding the 340B program isn't going away any time soon. At a minimum, the lawsuits are putting a fresh spotlight on the program and raising questions about whether it benefits patients… In addition to mandatory transparency requirements, Schwartz would like to see regulatory requirements allowing patients accessing drugs through a covered entity to have access to a sliding scale for purchases, so they don't have to pay list prices. Not all medication discounts are directly passed on to patients. "…We hope that all the stakeholders can come together and find ways to fix the program because the industry continues to be committed to this program. But it needs to work for patients." The AHA says that these actions are hurting hospitals and affecting their ability to care for patients. "It's just a very poor decision of manufacturers to enact these policies and hurt the providers and patients that needed the most," says Krishnamurthy." | N/A |

© 2023 Knox RP et al. *JAMA Health Forum.*

| Article Citation | Article Type | Study Objective/Article Thesis | Stakeholders Discussed | Results/Analyses | Conclusions/ Recommendations | Limitations |
|---|---|---|---|---|---|---|
| Kelly C. Pharma Gets 340B Appeals Court Victory But More Decisions Are Pending; Will Congress Step In?. Pink Sheet. Published Jan. 31, 2023. Accessed Apr. 10, 2023. https://pink.pharmaintelligence.informa.com/PS147663/Pharma-Gets-340B-Appeals-Court-Victory-But-More-Decisions-Are-Pending-Will-Congress-Step-In | Commentary | To discuss the litigation regarding manufacturer restrictions on 340B contract pharmacies | Hospitals, clinics, manufacturers, pharmacies | The article discusses the Third Circuit opinion permitting restrictions on contract pharmacy participation in the 340B Program. | N/A | N/A |
| Kimble C, Coutasse A. The 340B Program: Benefits and Limitations. Marshall University. Published Apr. 2018. Accessed Apr. 10, 2023. https://mds.marshall.edu/cgi/viewcontent.cgi?referer=&httpsredir=1&article=1193&context=mgmt_faculty | Presentation | "To analyze and quantify the financial impact and humanistic and challenges concerning the 340B program in the tri-state [ KY, OH, and WV] and quantify how program is meeting its intent in this area" | Hospitals, clinics | "• In 2016, hospitals provided nearly $38.3 billion in uncompensated care<br>• 45% of all Medicare acute care hospitals participate in the 340B program<br>• 2014–2016, the volume of purchases made through 340B more than doubled, expanding 125%.<br>• 340B Discount Purchases =$16.2 billion in 2016 — ($12 billion in 2015)<br>• 340B program grew at a compound annual growth rate of 31% (2013 to 2016).<br>• Average savings of 25% –50% through negotiated ceiling / sub-ceiling price" | "1. 340B does provide benefits to Vulnerable & Underserved patient by:<br>• Expanded services<br>• Increased access to healthcare.<br>• Increased access to prescription drugs<br>2. This type of benefit, while indirect, has fulfilled the intent of the 340B program at its inception." | N/A |

© 2023 Knox RP et al. *JAMA Health Forum.*

| Article Citation | Article Type | Study Objective/Article Thesis | Stakeholders Discussed | Results/Analyses | Conclusions/ Recommendations | Limitations |
|---|---|---|---|---|---|---|
| Kirby VB, Botta EM, Steward WT. The Role of the 340B Drug Pricing Program in HIV-Related Services in California. Accessed Apr. 10, 2023. https://www.californiaaidsresearch.org/files/340B-HIV-Rapid-Response-Report-FINAL.pdf | Report | To characterize the current use of the 340B Drug Pricing Program by health care entities that serve people living with HIV (PLWH) and vulnerable to HIV in California, and to assess possible changes to current HIV-related services and programs that may occur if a proposal by California's Governor Jerry Brown to eliminate the use of 340B discounts on Medi-Cal transactions is enacted. | Hospitals, clinics | "From March to April 2018, we interviewed 7 key informants across California. … 340B savings supported the provision of medications for free or at low cost to uninsured individuals, including PLWH, and savings reinvested by health care entities supported a range of services and staff positions geared towards increasing patient engagement, maintaining strong clinic infrastructure, and raising the standard of care. All of these activities are particularly crucial for PLWH (people living with HIV). Informants expressed a desire to continue participating in the 340B Program despite the significant administrative burden required to manage program participation and maintain compliance. Several informants expressed interest in working with the State to improve 340B functionality and avoid compliance issues." | "Our findings strongly support maintaining access to robust 340B savings as a vital source of support for comprehensive HIV-related services." | N/A |

© 2023 Knox RP et al. *JAMA Health Forum.*

| Article Citation | Article Type | Study Objective/Article Thesis | Stakeholders Discussed | Results/Analyses | Conclusions/ Recommendations | Limitations |
|---|---|---|---|---|---|---|
| Kolenich E. Delegate asks Bon Secours to explain how it's spending millions in drug savings. Richmond Times-Dispatch. Published Jan. 27, 2023. Accessed Apr. 10, 2023. https://richmond.com/news/state-and-regional/govt-and-politics/delegate-asks-bon-secours-to-explain-how-its-spending-millions-in-drug-savings/article_9baf83e6-9e44-11ed-8b04-57cf91a3a790.html | News | To review a proposed bill that would require hospitals to disclose how they use 340B revenues | Hospitals, clinics | "The bill calls for nonprofit hospitals that use the 340B program to estimate their savings, to show how they use those savings to benefit the community around the hospital and to make a commitment to oversight, ensuring the hospital system follows the law… The delegate modeled the bill off the American Hospital Association, which asks hospitals using 340B to commit to its good stewardship principles, which are the same three requirements made in the bill.<br><br>Six other hospitals in the state, including Virginia Commonwealth University Health, already made such a commitment. Currently, the federal government does not require hospitals to explain their savings and reinvestment, but scrutiny of the 340B program has grown since September." | N/A | N/A |

© 2023 Knox RP et al. *JAMA Health Forum.*

| Article Citation | Article Type | Study Objective/Article Thesis | Stakeholders Discussed | Results/Analyses | Conclusions/ Recommendations | Limitations |
|---|---|---|---|---|---|---|
| LaMattina J. Senate Bill 355 Would Divert Patient Savings to For-Profit Businesses. Connecticut Examiner. Published Apr. 30, 2022. Accessed Apr. 10, 2023. https://ctexaminer.com/2022/04/30/senate-bill-355-would-divert-patient-savings-to-for-profit-businesses/ | Letter to the Editor | To discuss the potential impact of a Senate bill on the 340B Program | Hospitals, clinics, pharmacies, patients | 1. "340B is now the second largest federal drug program behind only Medicare part D to the tune of $38 million in 2020. … Some hospitals in Connecticut do business through pharmacies in Texas and California. How is that benefitting Connecticut patients?"<br>2. "Even contracts at Connecticut's own Hartford Healthcare and Yale New Haven Health have skyrocketed according to data from HRSA … In 2011, Yale had one contract pharmacy and in 2022 has 266. In 2016, Hartford Health Care had 7 contracts and today has 148.<br>3. These are profitable relationships. A report from Berkley Research Group showed that the average profit margin on 340B medicines commonly dispensed through contract pharmacies is an estimated 72 percent, compared with just 22 percent for non-340B medicines dispensed through independent pharmacies.<br>4. Legislation like SB 355 has been rejected in Vermont and Maine and is still being scrutinized in California. Arkansas passed a variation of the bill, and it is now tied up in litigation. " | N/A | N/A |

© 2023 Knox RP et al. *JAMA Health Forum.*

| Article Citation | Article Type | Study Objective/Article Thesis | Stakeholders Discussed | Results/Analyses | Conclusions/ Recommendations | Limitations |
|---|---|---|---|---|---|---|
| Lin JK, Li P, Doshi J, Desai S. How a Federal Policy for Pharmacoequity Keeps Falling Short. Penn Leonard Davis Institute of Health Economics. Published Sept. 9, 2022. Accessed Apr. 10, 2023. https://ldi.upenn.edu/our-work/research-updates/how-a-federal-policy-for-pharmacoequity-keeps-falling-short/ | Commentary | To discuss the limitations of the 340B Program | Hospitals, clinics, manufacturers, pharmacies | "Such actions may curb the excesses of larger wealthy hospital systems; unfortunately, they also damage smaller safety net institutions. More than 130 rural hospitals closed over the past 10 years, with the number of closures hitting a high in 2020. In a survey sponsored by an advocacy group called 340B Health, rural hospitals reported that pharmaceutical company restrictions led to losses of $500,000 to $1 million per year. Blunt cuts to the 340B Program could make it even harder for struggling hospitals to stay open. Indeed, other researchers recently found, in contrast with 340B pharmacy contracts with hospitals, pharmacy contracts with safety net clinics have grown in areas with higher poverty rates." | "The 340B Program has great potential to help the most vulnerable Americans, but it is in need of reform. There is no shortage of opinions for how to do so, ranging from restricting the types of institutions that can be eligible to providing more explicit regulations on who gets to use the money and how—for instance by requiring that pharmacies and providers pass along some of the discounts to low-income patients struggling with co-pays. The tricky part will be getting any potential reform right so that the benefits are targeted to patients and institutions that need it most." | N/A |

© 2023 Knox RP et al. *JAMA Health Forum.*

| Article Citation | Article Type | Study Objective/Article Thesis | Stakeholders Discussed | Results/Analyses | Conclusions/ Recommendations | Limitations |
|---|---|---|---|---|---|---|
| Macherelli LE, Rosebush LH, Wagner MN. Discounted Drugs under the 340B Program. BakerHostetler. Published Nov. 21, 2022. Accessed Apr. 10, 2023. https://www.bakerlaw.com/alerts/discounted-drugs-under-340B-program | Commentary | To discuss litigation related to the 340B Program and contract pharmacies | Hospitals, clinics, manufacturers, pharmacies | "Pharmaceutical manufacturers argue that the 340B Program is not meant to provide discounted drugs to community pharmacies that contract with hospitals.<br>HHS argues that under the 340B Program, HHS has the authority to require drug companies to offer discounts for drugs purchased by safety-net hospitals and dispensed by community pharmacies.<br>Outcome will affect the size and scope of the 340B Program—potentially saving pharmaceutical manufacturers from providing broad discounts on drugs with no self-imposed restrictions or shifting the ability to obtain these drug discounts to community pharmacies." | "The implications of the court's decision in this case will reach providers, manufacturers, pharmacies and patients. If the court rules in favor of the drugmakers, it has the potential to dramatically limit the size and scope of the 340B program. However, if it were to find in favor of HHS, the 340B program would likely continue its expansive reach and continue to grow, serving patients across the country." | N/A |

© 2023 Knox RP et al. *JAMA Health Forum.*

| Article Citation | Article Type | Study Objective/Article Thesis | Stakeholders Discussed | Results/Analyses | Conclusions/ Recommendations | Limitations |
| --- | --- | --- | --- | --- | --- | --- |
| Martin R, Hasan S. Growth of the 340B Program Accelerates in 2020. IQVIA. Published Mar. 31, 2021. Accessed Apr. 10, 2023. https://www.iqvia.com/locations/united-states/blogs/2021/03/growth-of-the-340b-program-accelerates-in-2020 | Commentary | To assess the impact of COVID-19 on the growth of 340B program | Hospitals, clinics | 1. * "Sales for the 340B Program hit $80.1B in 2020, an 18.1% year-on-year growth versus 2019, and very close to a forecast made by Nephron Research in October 20202. … 340B sales growth was over four and a half times the overall pharma market growth rate of 4.0%. Since 2017, 340B sales have grown 76%."<br>2. Growth occurred in specialty drug products (particularly in disease areas with high-priced specialty products) and mail-order pharmacies.<br>3. "In healthcare, COVID-19 is thought to have caused a shortfall of a billion diagnosis visits in 2020, drove a 3,000% increase in telehealth visits (albeit from a relatively small baseline), and caused treatment to shift from hospitals to offsite facilities" | N/A | N/A |

© 2023 Knox RP et al. *JAMA Health Forum.*

| Article Citation | Article Type | Study Objective/Article Thesis | Stakeholders Discussed | Results/Analyses | Conclusions/ Recommendations | Limitations |
|---|---|---|---|---|---|---|
| Martin R. 340B Program Continues to Grow While Contract Pharmacy Restrictions Take Effect. IQVIA. Published Apr. 5, 2022. Accessed Apr. 10, 2023. https://www.iqvia.com/locations/united-states/blogs/2022/04/340b-program-continues-to-grow-while-contract-pharmacy-restrictions-take-effect | Report | To provide "estimates of the 340B program's size and growth as part of an ongoing program-related series of article" | Hospitals, clinics, manufacturers, pharmacies, patients | "In 2021, 340B program sales reached $93.6 billion (Figure 1), versus total pharmaceutical sales of $668.3 billion (see Analysis Methods for further details about the data and methodology used). 340B sales grew 15.9% year-on-year in 2021 and total sales grew 6.6%, versus 18.1% and 4.0% growth in 2020, respectively…. Disease areas with specialty products, such as targeted oncology, immunology, and anti-arthritics including well-known blockbuster biologic products, continued to show some of the highest 340B growth in 2021… For the nine manufacturers with restrictions in place for some or all of 2021, retail and mail 340B sales fell 32% and 20%, respectively. For sales among all other manufacturers, retail and mail 340B sales grew 16% and 44%, respectively. 340B sales in the hospital and clinic channels were similar for the two manufacturer groups. 340B sales in the mass merchandizer channel fell 60% for manufacturers with restrictions in 2021." | N/A | N/A |

© 2023 Knox RP et al. *JAMA Health Forum.*

| Article Citation | Article Type | Study Objective/Article Thesis | Stakeholders Discussed | Results/Analyses | Conclusions/ Recommendations | Limitations |
|---|---|---|---|---|---|---|
| Masia N. 340B Drug Pricing Program: Analysis Reveals $40 Billion in Profits in 2019. 340B Reform. Published May 2021. Accessed Apr. 10, 2023. https://340breform.org/wp-content/uploads/2021/05/AIR340B-Neal-Masia-Report.pdf | Report | To measure and evaluate the growth of the 340B Program | Hospitals, clinics, manufacturers, pharmacies | 1) "We estimate that provider profits have more than doubled, from $20.2 billion in 2015 to $40.5 billion in 2019." 2) "while physician-administered products have shown steady growth, profits from self-administered drugs have been much greater" 3) "Oncology's share of 340B sales has increased from 37.5% in 2015 to 52.4% in 2019, and oncology-generated 340B profits increased from 19.1% of total 340B provider to 32.6% over the same period" 4) "If instead we calculate pharmacy shares as 15% of total revenue, pharmacies would have captured roughly $7 billion in 2019." | "The 340B program has grown exponentially over the past decade and has become a major source of funding for thousands of grantees and hospitals. Our estimates provide a new, somewhat more precise way to estimate the profits associated with the program and how they have changed over time. It is up to policymakers to determine whether these changes are consistent with the original program's intent." | N/A |

© 2023 Knox RP et al. *JAMA Health Forum.*

| Article Citation | Article Type | Study Objective/Article Thesis | Stakeholders Discussed | Results/Analyses | Conclusions/ Recommendations | Limitations |
|---|---|---|---|---|---|---|
| McCaughan M. The 340B Drug Discount Program. Health Affairs. Published Sept. 14, 2017. Accessed Apr. 10, 2023. https://www.healthaffairs.org/do/10.1377/hpb20171024.663441/full/ | Report | To describe the 340B Program and its critiques | Hospitals, clinics, | 1. " Critics argue that the program has strayed far from providing access to drugs for safety-net providers and their patients and has instead become a new funding stream for public hospitals, including those that provide relatively limited amounts of uncompensated care."<br>2. "The post-ACA expansion of the 340B program has coincided with a shift in treatment patterns for oncology, from community-based private practices to hospital outpatient departments. Many community oncologists see a cause-and-effect relationship, arguing that they can't compete with the margins available to 340B-eligible hospitals, which can purchase high- price cancer therapies at deeply discounted prices."<br>3. "340B pricing encourages providers to choose a higher-cost agent, even when a lower-cost therapy is available, because the spread will be larger and the profit margin therefore higher." | N/A | N/A |

© 2023 Knox RP et al. *JAMA Health Forum.*

| Article Citation | Article Type | Study Objective/Article Thesis | Stakeholders Discussed | Results/Analyses | Conclusions/ Recommendations | Limitations |
|---|---|---|---|---|---|---|
| Mulligan K. The 340B Drug Pricing Program: Background, Ongoing Challenges and Recent Developments. University of Southern California Leonard D. Schaeffer Center for Health Policy & Economics. Published Oct. 14, 2021. Accessed Apr. 10, 2023. https://healthpolicy.usc.edu/research/the-340b-drug-pricing-program-background-ongoing-challenges-and-recent-developments/ | Report | To evaluate the impact and challenges of the 340B Program | Hospitals, clinics, manufacturers, pharmacies | "The 340B Drug Pricing Program allows eligible healthcare clinics and hospitals ("covered entities") to purchase outpatient drugs at a 20-50% discount.<br>This program is unusual among federal programs in that it involves a mandatory transfer of resources from one group of private entities (manufacturers/wholesalers) to another (providers).<br>Between 2000 and 2020, the number of covered entity sites participating in the 340B program increased from 8,100 to 50,000. Hospitals comprised just over 60% of sites in 2020.<br>Estimated discounted purchases through the program have increased from about $4 billion per year in 2007-2009 to $38 billion in 2020.<br>The 340B program faces ongoing controversy in several areas: limited program oversight, transparency in how covered entities use funds generated by the program, and the growth in participation by contract pharmacies and DSH hospitals." | "Over the last 30 years, the growth of the 340B program coupled with a lack of legislative reforms has created a challenging situation for private stakeholders and left the terms guiding the transfer of billions of dollars in value annually up to private actors and the courts. Until Congress can pass legislation that effectively modifies the 340B program or its oversight, manufacturers and covered entities, particularly hospitals, will likely continue to test the bounds of the program. There are valid concerns with the 340B program related to transparency, diversion, and duplicate discounts, particularly through contract pharmacies and hospitals. However, the 340B Program has also provided significant resources to many providers and allowed them to better serve millions of patients. Congressional reform efforts will need to take both perspectives into account to sustainably modify the program." | N/A |

© 2023 Knox RP et al. *JAMA Health Forum.*

| Article Citation | Article Type | Study Objective/Article Thesis | Stakeholders Discussed | Results/Analyses | Conclusions/ Recommendations | Limitations |
|---|---|---|---|---|---|---|
| New White Paper: The Impact of the 340B Program on Drug Prices Charged by Manufacturers and Covered Entities. Leavitt Partners. Published Dec. 1, 2022. Accessed Apr. 10, 2023. https://leavittpartners.com/the-impact-of-the-340b-program-on-drug-prices-charged-by-manufacturers-and-covered-entities/ | Report | To provide insight on the impact of the 340B Program on drug pricing | Manufacturers | "The 340B program is a mandatory program for pharmaceutical manufacturers wishing to participate in the Medicaid drug rebate program. Today, the program has more than 53,000 participating covered entities and the total amount of drugs purchased at the 340B ceiling price under the program is almost $44 billion (Drug Channels). Drug list prices, like other prices in health care, are increasing. The research for this white paper suggests that 340B is one of many factors putting upward pressure on launch prices. Covered entities can generate savings from the prices charged for 340B drugs. There are no requirements for hospitals for how they use the savings, so pricing for services vary. Lack of comprehensive data across the program limits insights on pricing and discount strategies." | N/A | N/A |

© 2023 Knox RP et al. *JAMA Health Forum.*

| Article Citation | Article Type | Study Objective/Article Thesis | Stakeholders Discussed | Results/Analyses | Conclusions/ Recommendations | Limitations |
|---|---|---|---|---|---|---|
| NorthBay Health: Good Stewardship of the 340B Program. NorthBay Health. Accessed Apr. 10, 2023. https://www.northbay.org/about/340B-Program.cfm | Hospital Webpage | To describe the hospital's experience with the 340B Program | Hospital, patients | *"provided $132.4 million worth of uncompensated care in 2019"<br>* "Expanded medical care to remote service areas;<br>* Provided Covid-19 vaccine clinics for the community;<br>* Maintained a wealth of current services and procedures so that uninsured patients do not need to travel long distances for medical care;<br>* Improved access and increased patient care for the uninsured, under-insured and other vulnerable populations;<br>* Provided procedures and services to patients at a loss;<br>* Developed new service lines such as the Transition of care clinic and Hypertension clinic to ensure patients are discharged with access to appropriate medication to prevent re-admissions; and<br>* Provided a patient assistance program for retail prescriptions for uninsured and under-insured." | N/A | N/A |

© 2023 Knox RP et al. *JAMA Health Forum.*

| Article Citation | Article Type | Study Objective/Article Thesis | Stakeholders Discussed | Results/Analyses | Conclusions/ Recommendations | Limitations |
|---|---|---|---|---|---|---|
| O'Brien J.M. After 30 years of 340B, it's time for data and an honest conversation. STAT News. Published Oct. 26, 2022. Accessed Apr. 10, 2023. https://www.statnews.com/2022/10/26/after-30-years-of-340b-time-for-data-honest-conversation/ | News | To evaluate the development of the 340B Program | Hospitals, clinics, | "Research on 340B suggests a complicated story, with the program's growth in spending boosting hospitals' bottom lines. Press accounts have highlighted the importance that 340B profits play in health center acquisitions, rather than providing care to underserved communities. More recent articles have pointed out that some highly profitable hospital systems are using poor neighborhoods to generate 340B revenue while investing profits into hospitals in affluent communities."<br><br>There is an need for additional transparency requirements. | "The data released on 340B to date indicate there are fundamental questions about this program that need to be answered. I commend every team of researchers that has attempted to peer into 340B's murky waters, because it is a big challenge. But it's critical to answer these questions because, as my colleagues and I often say, good policy requires thorough research.<br><br>If hospitals, contract pharmacies, and third-party administrators have more data that can add more detail to this discussion, the research community must be given access to it so their work can restore confidence that the program is helping those it is supposed to help.<br><br>It's time to have an honest conversation and recognize that the 340B program has grown far beyond its intended purpose. Protecting the charity care it provides requires measuring and ferreting out the profiteering the law has enabled. An important first step is more transparency into how 340B works and who is truly benefiting from it." | N/A |

© 2023 Knox RP et al. *JAMA Health Forum.*

| Article Citation | Article Type | Study Objective/Article Thesis | Stakeholders Discussed | Results/Analyses | Conclusions/ Recommendations | Limitations |
|---|---|---|---|---|---|---|
| Orwig S. The 340B drug pricing controversy, explained. Advisory Board. Published Dec. 12, 2017. Accessed Apr. 10, 2023. https://www.advisory.com/daily-briefing/2017/12/12/340b-explained | Commentary | To help the reader "to understand the 340B program, how it works, who it serves, why it's controversial, and what reimbursement cuts could mean for providers." | Hospitals, clinics, patients | "CMS says this reimbursement cut will lower out-of-pocket drug costs for Medicare patients. The agency estimates that patients will save $320 million in 2018 on coinsurance alone, since Medicare will be paying less for the drugs. However, because the cut targets only reimbursement, it would not affect how drug manufacturers set prices, nor would it change prices paid by wholesalers or providers.<br><br>On a more technical level, the rule also introduces new coding requirements for 340B hospitals starting in 2018. Covered entities that are subject to the reimbursement cut must add the modifier "JG" to claims when the drugs were purchased at the 340B rate. Covered entities that are exempt from the new cuts must add the modifier "TB" when the drugs were purchased at 340B rates. Presumably, CMS hopes to use the data to better determine how 340B influences Part B prescribing and thus Medicare spending." | N/A | N/A |

© 2023 Knox RP et al. *JAMA Health Forum.*

| Article Citation | Article Type | Study Objective/Article Thesis | Stakeholders Discussed | Results/Analyses | Conclusions/ Recommendations | Limitations |
|---|---|---|---|---|---|---|
| Our 340B Program. UCSF Benioff Children's Hospitals. Accessed Apr. 10, 2023. https://www.ucsfbenioffchildrens.org/about/our-340b-program | Hospital Webpage | To describe the hospital's experience with the 340B Program | Hospital, patients | The hospital uses the 340B Program to: "Ensure Medicaid and uninsured patients can access specialty clinics that provide lifesaving treatments ….<br>* Provide specialized care for thousands of children with life-challenging medical conditions, such as cancer, organ failure, genetic diseases, heart defects and epilepsy…<br>* Ensure that Medicaid and uninsured patients can access clinics that treat illnesses such as HIV, hepatitis C and asthma, which disproportionately impact vulnerable patients.<br>* Subsidize costly chemotherapy and immunotherapy for Medicaid and uninsured patients…<br>* Provide financial assistance to patients who can't afford their prescriptions…<br>* Enable low-income and homeless individuals in our community to access specialty care that is otherwise not available to them….<br>* Sponsor outreach events to Bay Area seniors to review the Part D plans that will be most cost effective for each senior and to maximize their existing drug benefits." | N/A | N/A |

© 2023 Knox RP et al. *JAMA Health Forum.*

| Article Citation | Article Type | Study Objective/Article Thesis | Stakeholders Discussed | Results/Analyses | Conclusions/ Recommendations | Limitations |
|---|---|---|---|---|---|---|
| Our 340B Story. UCSF Health. Accessed Apr. 10, 2023. https://www.ucsfhealth.org/about/our-340b-story | Hospital Webpage | To describe the hospital's experience with the 340B Program | Hospital, patients | The 340B Program allows UCSF to: "Underwriting the cost of promising new treatments, such as CAR T-cell therapy for cancer and autoimmune disease Expanding access to specialty programs that perform lifesaving treatments, including organ transplants, complex cancer care, immunotherapies (such as bone marrow transplants), neurological treatments and neurosurgery, cardiovascular care and cardiothoracic surgery Sponsoring educational outreach events to help older adults in the Bay Area choose the most cost-effective Medicare Part D plans and maximize their existing drug benefits Subsidizing pharmacists who provide HIV management in clinics that largely serve Medicaid patients Employing patient assistance coordinators dedicated to removing financial barriers for our patients Providing chemotherapy and immunotherapy for Medi-Cal and uninsured patients at infusion centers and clinics, based on financial need Running a food bank that provides groceries to patients experiencing food insecurity." | "At UCSF Health, we are deeply committed to our mission to care, heal, teach and discover. In the face of declining reimbursements over the years, UCSF Health has resisted reducing mission-critical services by using 340B program funding to maintain services essential to supporting uninsured and underinsured patients. Manufacturers have removed 340B pricing at contract pharmacies, directly affecting patients who participate in our community benefits program. Not only can these patients no longer afford high-cost medications that they need, but UCSF may also lose the funding required to provide or subsidize a wide range of essential services that thousands of patients rely on every year." | N/A |

© 2023 Knox RP et al. *JAMA Health Forum.*

| Article Citation | Article Type | Study Objective/Article Thesis | Stakeholders Discussed | Results/Analyses | Conclusions/ Recommendations | Limitations |
|---|---|---|---|---|---|---|
| Paul DP, Ludado MC, Ruley M, Sayre H, Coutasse A. The 340B Program, Contract Pharmacies and Hospitals: An Examination of the First 25 Years of their Increasingly Complex Relationship. Northeast Business & Economics Association Proceedings. Published 2018. Accessed Apr. 10, 2023. https://mds.marshall.edu/cgi/viewcontent.cgi?article=1204&context=mgmt_faculty | Report | To examine how the 340B Program has affected hospitals and contract pharmacies since its inception, and what might be required to better meet the needs of its intended target, low-income Americans. | Hospitals, pharmacies | "The federal 340B Drug Pricing Program has provided access to reduced price prescription drugs to over 35,000 individual healthcare facilities and sites certified by the U.S. Department of Health and Human Services (HHS), and clinics have served more than 10 million people in all 50 states, plus commonwealths and U.S. territories. The 340B program has increased profits for hospitals through contract pharmacies because they have still received the same reimbursement but acquired drugs at a lower rate." | "The federal 340B Drug Pricing Program has continued to grow since being signed into law in 1992. Although it was originally designed to serve the needs of low-income Americans, it appears to have morphed into a mechanism for healthcare providers to enrich their bottom lines regardless of the income level of the population they serve, although the latter conclusion is disputable, depending on one's point of view. Additional, more comprehensive data obtained using a better cost accounting system for providers and analysis by impartial parties will be necessary if the questions are to be resolved." | N/A |

© 2023 Knox RP et al. *JAMA Health Forum.*

| Article Citation | Article Type | Study Objective/Article Thesis | Stakeholders Discussed | Results/Analyses | Conclusions/ Recommendations | Limitations |
|---|---|---|---|---|---|---|
| Pearson E, Frakt A. 340B is a well-intentioned drug discount program gone awry. STAT News. Published Mar. 22, 2018. Accessed Apr. 10, 2023. https://www.statnews.com/2018/03/22/340b-drug-discount-program-gone-awry/ | Commentary | To discuss the limitations of the 340B Program and the need for reforms | Hospitals, clinics, manufacturers | "Several reforms to the program have been proposed that could help limit the unintended consequences and encourage hospitals to use the drug discounts to advance the program's goals. For example, members of Congress have suggested that hospitals and clinics be held accountable for all cost savings accrued under the 340B program. Sen. Chuck Grassley introduced a bill to require hospitals and clinics to report both total costs of 340B drugs and total revenue from insurance companies for those same drugs. Others have suggested that all profit from the 340B program should be reinvested into the high-risk, high-need populations that warranted eligibility in the first place. Finally, the House Committee on Energy and Commerce proposed revising the eligibility for the 340B program so that it admits only organizations that serve large populations of high-risk, high-need patients, ensuring the intent of the program is intact." | "The goal of assisting hospitals and clinics that provide care to vulnerable populations is noble. Corruption does not negate potential, nor does reform equate to failure. Long-term sustainability of the 340B program requires timely action that seeks to restore the program to its original intent." | N/A |

© 2023 Knox RP et al. *JAMA Health Forum.*

| Article Citation | Article Type | Study Objective/Article Thesis | Stakeholders Discussed | Results/Analyses | Conclusions/ Recommendations | Limitations |
|---|---|---|---|---|---|---|
| Pipes SC. How one bad law drives hospital consolidation and high health care costs. The Dallas Morning News. Published Jan. 28, 2023. Accessed Apr. 10, 2023. https://www.dallasnews.com/opinion/commentary/2023/01/28/how-one-bad-law-drives-hospital-consolidation-and-high-healthcare-costs/ | News | To discuss the impact of the 340B Program | Hospitals, clinics | "While it's clearly not fulfilling its intended purpose, the 340B program is driving up health care costs by incentivizing hospital mergers. As the law is written, a hospital can get those up-to-half-off discounts at any facility it operates, including satellite clinics. And the discounts strengthen incentives for hospitals to resell drugs to middle-class and well-off patients who have generous insurance coverage.<br><br>All this has encouraged acquisitions to the point that now, the 10 largest health care systems in the United States control nearly one-quarter of all hospitals. In short, large hospital systems are exploiting the law to sweep ever-larger swaths of the health care system into 340B, including facilities in affluent areas. The number of hospitals and clinics enrolled in the program increased by an astonishing 517% from 2000 to 2020. Hospital income is up accordingly: From 2013 to 2018, the 10 largest health systems saw total patient revenue increase 82%, from $505 billion to $918 billion, according to research from Deloitte Insights." | "The only solution is for Congress to revise the law, enacting safeguards that return 340B to its intended purpose.<br><br>A good place to start would be cracking down on eligibility. Program access should be limited to health care facilities that actually serve low-income patients. Next, 340B hospitals should have to use their discounts to benefit the target population and document how they're doing so." | N/A |

© 2023 Knox RP et al. *JAMA Health Forum.*

| Article Citation | Article Type | Study Objective/Article Thesis | Stakeholders Discussed | Results/Analyses | Conclusions/ Recommendations | Limitations |
|---|---|---|---|---|---|---|
| Pollack R. 340B drug pricing program helps advance health for patients, communities. The Hill. Published Oct. 10, 2022. Accessed Apr. 10, 2023. https://thehill.com/opinion/congress-blog/3681858-340b-drug-pricing-program-helps-advance-health-for-patients-communities/ | News | To discuss the benefits of the 340B Program | Hospitals, clinics, patients | "Examples of the 340B program expanding access to care include: 340B helps Henry Ford Health in Michigan embed pharmacists in primary care and specialty clinics to treat chronic diseases and provide additional medication services for all patients… 340B helps Meadville Medical Center in Pennsylvania offer oncology services so cancer patients in rural areas do not need to travel long distances for treatment, as well as offering dental and behavioral health services at their rural health clinics. 340B helps Mount Carmel Health System in Ohio go beyond the walls of its hospital to serve its community and patients through programs such as Street Medicine, which provides free urgent medical care to underinsured or uninsured community members. 340B helps Johns Hopkins Hospital in Maryland provide low-income patients with free and discounted outpatient drugs and other services, including telephone consultations, home visits and transportation services." | "Particularly in this era of rising drug prices, 340B has been core to helping hospitals expand access to comprehensive health services, including lifesaving prescription drugs for those who need them but may not be able to afford them. It is in everyone's best interest to keep the 340B program strong so that our nation's patients and communities can continue to receive the high-quality care and services on which they depend." | N/A |

© 2023 Knox RP et al. *JAMA Health Forum.*

| Article Citation | Article Type | Study Objective/Article Thesis | Stakeholders Discussed | Results/Analyses | Conclusions/ Recommendations | Limitations |
|---|---|---|---|---|---|---|
| Protect 340B Drug Pricing Program. Wisconsin Hospital Association. Published Jan. 2019. Accessed Apr. 10, 2023. https://www.wha.org/Home/Common-PDFs/340BIssuePaper2-6-19 | Report | To describe the impact of the 340B Program in Wisconsin | Hospitals, clinics, patients | “* The 340B program is an important program that helps hospitals extend care in their communities. * The program costs the federal government nothing and actually saves money in Medicare by allowing for lower reimbursements to critical access hospitals. * While Congress has considered additional oversight, hospitals already devote significant resources to ensure program integrity compliance.” | “* 340B continues to be an important program that stretches scarce federal resources for more than 70 hospitals in Wisconsin and helps them extend important services in their communities. * Participants in the 340B program already devote significant resources, including conducting internal audits, toward compliance with federal 340B program rules. * The Wisconsin Hospital Association and its members are committed to good stewardship of taxpayer dollars and strongly support reversing the cuts made in the 2018 OPPS rule while protecting access to the 340B program for all participating hospitals.” | N/A |
| Renfrow J. 340B program may hinder access to costly drugs as hospitals exploit discounts: CRE. Fierce Healthcare. Published Apr. 19, 2019. Accessed Apr. 10, 2023. https://www.fiercehealthcare.com/hospitals-health-systems/340b-hospitals-reducing-uninsured-access-to-quality-healthcare-cre | News | To describe a report from the Center for Regulatory Effectiveness finding “the 340B drug discount program may have actually hurt patient access to costly drugs as a growing number of hospitals take advantage of the program” | Hospitals, clinics, patients | “the number of uninsured, impoverished patients without access to medical care and dental care grew between 2009 and 2015, even as the overall number of 340B hospitals more than tripled” “Research also suggests that providers are not reinvesting the savings from 340B discounts in lowering the cost of care” | “The group concludes that the 340B program is responsible for three negative outcomes: increased inequality in the healthcare system, changed treatment protocols to maximize hospital’s drug profits and worsened medical outcomes for the impoverished and uninsured.” | N/A |

© 2023 Knox RP et al. *JAMA Health Forum.*

| Article Citation | Article Type | Study Objective/Article Thesis | Stakeholders Discussed | Results/Analyses | Conclusions/ Recommendations | Limitations |
|---|---|---|---|---|---|---|
| Report to Congress: Overview of the 340B Drug Pricing Program. Medicare Payment Advisory Commission. Published May 2015. Accessed Apr. 10, 2023. https://www.medpac.gov/wp-content/uploads/import_data/scrape_files/docs/default-source/reports/may-2015-report-to-the-congress-overview-of-the-340b-drug-pricing-program.pdf | Report/Presentation | To provide an overview of current issues in the 340B Program applicable to Medicare | Hospitals, manufacturers, HRSA | "In 2013, 65 percent of hospital sites and 37 percent of nonhospital sites carved in Medicaid patients (i.e., they provided 340B drugs to Medicaid patients)." "The Health Resources and Services Administration (HRSA), which manages the program, estimates that covered entities saved $3.8 billion on outpatient drugs through the program in fiscal year 2013….." • "The 340B program has grown substantially during the past decade. Covered entities and their affiliated sites spent over $7 billion to purchase 340B drugs in 2013, three times the amount spent in 2005. The number of hospital organizations … participating in 340B grew from 583 in 2005 to 1,365 in 2010 and to 2,140 in 2014." •"From 2004 to 2013, Medicare spending in nominal dollars for Part B drugs at hospitals that participate in 340B grew from $0.5 billion to $3.5 billion, or 543 percent. Hospitals in the 340B program accounted for 22 percent of Medicare spending for Part B drugs at all Medicare acute care hospitals in 2004, growing to 48 percent in 2013." | N/A | N/A |

© 2023 Knox RP et al. *JAMA Health Forum.*

| Article Citation | Article Type | Study Objective/Article Thesis | Stakeholders Discussed | Results/Analyses | Conclusions/ Recommendations | Limitations |
|---|---|---|---|---|---|---|
| Roberts B. The Controversy Surrounding 340B Program. American Legislative Exchange Council. Published Feb. 28, 2022. Accessed Apr. 10, 2023. https://alec.org/article/the-controversy-surrounding-340b-program/ | Commentary | To discuss ongoing controversies related to the 340B Program and related state actions | Hospitals, clinics, manufacturers, pharmacies | "Last year Arkansas passed legislation mandating drug manufacturers sell to all contract pharmacies at the 340(B) rate and preventing claims modifier requirements. Essentially the state was expanding the 340(B) program beyond what is in the federal statute, which does not address contract pharmacies. Despite several lawsuits to clarify the requirements of the federal statute and the issue of contract pharmacies, other states have tried to pass similar legislation this year Requiring manufacturers to sell to contract pharmacies at the 340(B) discount is price-fixing. Unfortunately, many legislators on both sides of the aisle don't see it that way. Government fixed prices of prescription drugs will not benefit the patients. Instead, it will cost us cures, innovation, and research from the pharmaceutical industry. Find out more about that here." | "History tells us time and again that government solutions to problems rarely have the intended effect. Legislators understandably worry about the cost of prescription drugs and making sure their constituents have access to the medicines they need; however, fixing the price of drugs is not the way to accomplish that goal. Opportunities for meaningful reform exist for policymakers to consider methods to provide rebates directly to patients, rather than pharmacies. As to 340(B), policymakers should look to the supply chain and ways to keep companies from hoarding the savings derived from discount programs. Affordable medicine is essential for the well-being of society, but a careful balance must be struck to guarantee that manufacturers will continue to invest and produce." | N/A |

© 2023 Knox RP et al. *JAMA Health Forum.*

| Article Citation | Article Type | Study Objective/Article Thesis | Stakeholders Discussed | Results/Analyses | Conclusions/ Recommendations | Limitations |
|---|---|---|---|---|---|---|
| Rogers H. Overview of the 340B Drug Discount Program. Congressional Research Service. Published Oct. 14, 2022. Accessed Apr. 10, 2023. https://crsreports.congress.gov/product/pdf/IF/IF12232 | Report | To detail the 340B Program and recent legal and political challenges | Hospitals, clinics, manufacturers, pharmacies | GAO has made "two main recommendations for oversight and improvement. First, GAO recommends that HRSA increase its oversight of covered entities, and particularly DSHs, to ensure they meet program eligibility requirements. Second, GAO suggests that HRSA and the Centers for Medicare & Medicaid (CMS) enhance their oversight of the 340B and Medicaid rebate programs, respectively, to ensure that covered entities are not receiving duplicate discounts on 340B-covered drugs."<br><br>Litigation is ongoing regarding the use of contract pharmacies in the 340B Program. | "Congress could amend the 340B statute to clarify the role contract pharmacies should play in the 340B Program. … Congress could amend the statute to clarify how covered entities may use the profits attributable to 340B drugs. Congress could also require DSHs seeking to become covered entities to more clearly demonstrate their eligibility for 340B pricing, including by detailing the scope of care the hospitals provide to underserved patients who are ineligible for Medicare or Medicaid.<br>Congress could also amend the 340B statute to increase HRSA's authority to regulate the Program and its participants. In its FY2023 Budget Justification, HRSA proposed a statutory amendment to provide the agency with rulemaking authority in order to strengthen its oversight of the Program." | N/A |

© 2023 Knox RP et al. *JAMA Health Forum.*

| Article Citation | Article Type | Study Objective/Article Thesis | Stakeholders Discussed | Results/Analyses | Conclusions/ Recommendations | Limitations |
|---|---|---|---|---|---|---|
| Rosenthal M. 340B Program: Important, but Weaknesses Cited. Pharmacy Practice News. Published Jan. 31, 2018. Accessed Apr. 10, 2023. https://www.pharmacypracticenews.com/Policy/Article/02-18/340B-Program-Important-but-Weaknesses-Cited/46797?sub=31D69684AEB1E89FB835F42F5E7AFCABF2EB378D903AF6B27750490CC64ED30&enl=true | Commentary | To evaluate the strengths and weaknesses of the 340B Program | Hospitals, clinics, federal government | * "A federal court limited HRSA's regulatory authority in 2014 to three areas: resolving disputes relating to compliance with the program's requirements; imposing civil monetary penalties against manufacturers that intentionally overcharge a coverer entity; and defining standards for calculating 340B drug prices."<br>- "Much of the controversy is due to the scope of 340B. In 1992, 29 million people were enrolled in Medicaid, and the Medicaid program cost $120 billion. In 2016, more than 72 million people were enrolled in Medicaid, costing more than $575 billion. The 340B program kept pace with Medicaid growth, not only in the number of covered entities and contract pharmacies but also in the amount they saved.<br>Legislating more change | "Sen. Bill Cassidy, MD (R-La.), introduced the HELP ACT (Helping Ensure Low-income Patients have Access to Care and Treatment), which would increase the program's transparency by requiring facilities to report how much charity care is provided at outpatient clinics and the insurance status of patients who receive 340B medications. [...] Congressmen Larry Bucshon, MD (R-Ind.) and Scott Peters (D-Ca.) introduced the 340B PAUSE Act, which pauses new enrollment of safety net hospitals for two years."<br>- "Examples in the E&C report include mobile medical clinics, preventive services, pharmacist counseling, community health centers and other services that would not be possible if these health systems did not receive a discount on certain medications. Still, the report recommended increased transparency to allow more accurate accounting of the full scope of the program's use and benefits, among other oversight enhancements." | N/A |

© 2023 Knox RP et al. *JAMA Health Forum.*

| Article Citation | Article Type | Study Objective/Article Thesis | Stakeholders Discussed | Results/Analyses | Conclusions/ Recommendations | Limitations |
|---|---|---|---|---|---|---|
| Rural Hospitals' Experience with the 340B Drug Pricing Program. University of North Carolina \| The Cecil G. Sheps Center for Health Services Research. Accessed Apr. 10, 2023. https://www.shepscenter.unc.edu/product/rural-hospitals-experience-with-the-340b-drug-pricing-program/ | Abstract | "two surveys were conducted, one of rural hospitals eligible for the program but not participating, and the other focusing on participating hospitals." | Hospitals | N/A | N/A | N/A |
| Salib V. Unpacking the 340B Drug Pricing Program, Its Impact on the Pharmaceutical Industry. Pharma News Intelligence. Published Nov. 9, 2022. Accessed Apr. 10, 2023. https://pharmanewsintel.com/features/unpacking-the-340b-drug-pricing-program-its-impact-on-the-pharmaceutical-industry | News | To describe a report from the Commonwealth Fund on the 340B Program | Hospitals, clinics, manufacturers | "The 340B Program has resulted in a loss of profit for many in the pharmaceutical industry, leaving drugmakers and industry leaders frustrated and critical of the program. The Commonwealth Fund states that the value of the discounts from the 340B program in 2021 was nearly $44 billion, a 16% increase from the previous year. Having to provide medications at reduced costs has cut the profit margins for drugmakers.<br><br>While the losses for drugmakers and manufacturers have incited criticism, it is thought that pharmacies contracted by eligible entities have profited. The number of contract pharmacies went from 1,700 in 2010 to 31,000 in 2021." | "Many pharmaceutical industry members are calling for reform to ensure that large healthcare facilities and chain pharmacies are not abusing the program. The first proposed solution by PhRMA is to ensure the patients are being put first.<br><br>Additionally, the organization calls for the government to provide more explicit guidelines on who will benefit from the programs. … Additionally, the pharmaceutical industry is calling for additional transparency about how healthcare facilities will be using the discount provided by the 340b program. PhRMA believes that there should be reporting requirements to hold these key players accountable. | N/A |

© 2023 Knox RP et al. *JAMA Health Forum.*

| Article Citation | Article Type | Study Objective/Article Thesis | Stakeholders Discussed | Results/Analyses | Conclusions/ Recommendations | Limitations |
|---|---|---|---|---|---|---|
| Schur C, Slifkin RT. Rural Hospital Participation in the 340B Drug Discount Program. University of North Carolina \| The Cecil G. Sheps Center for Health Services Research. Published Aug. 2007. Accessed Apr. 10, 2023. https://www.shepscenter.unc.edu/product/rural-hospital-participation-in-the-340b-drug-discount-program/ | Abstract | To understand the perspectives of pharmacy directors at participating and nonparticipating hospitals to understand their reasons for participation or non-participations, the challenges faced, the financial impact of the program, and which specific program features present barriers to its broader implementation | Hospitals, clinics, pharmacies | "Despite widespread efforts at dissemination about the program, over half of the responding pharmacy directors at non-participating hospitals (56%) were not aware their hospitals were eligible to participate in the program. … eligible rural hospitals do not seem to acquire all of the information they need to understand, evaluate, and implement the program.<br><br>The proportion of eligible hospitals participating in the 340B program appears to rise with revenue, from 28 percent of hospitals with less than $50 million participating in the program, to 61 percent of hospitals with over $100 million in annual revenue. The distribution of eligible rural hospitals is also quite skewed geographically, with a disproportionate share in the South. The average monthly savings for rural participating hospitals is approximately $19,700 on total outpatient drugs; some hospitals reported saving an average of 24 percent of the pharmacy budget. About 96 percent of all respondents stated that they were satisfied with the discount they received." | N/A | N/A |

© 2023 Knox RP et al. *JAMA Health Forum.*

| Article Citation | Article Type | Study Objective/Article Thesis | Stakeholders Discussed | Results/Analyses | Conclusions/ Recommendations | Limitations |
|---|---|---|---|---|---|---|
| Shusterman M. Shusterman: Time to reform the 340B drug pricing program. Rep. Melissa Shusterman. Published Apr. 26, 2022. Accessed Apr. 10, 2023. https://www.pahouse.com/Shusterman/InTheNews/Opinion/?id=124613 | Commentary | To advocate for reform of the 340B Program | Hospitals, clinics, manufacturers, pharmacies | "This program was supposed to help the poor, underinsured and uninsured but, clearly, it is not. Inadequate oversight and a lack of transparency have allowed this once beneficial program to become a part of our country's overspending on a floundering health care system.<br><br>With these 340B hospitals making more money and giving away less in charity care, the actual cost of the program's lack of regulation, coupled with its unprecedented massive growth, is felt by both uninsured and insured patients across the board." | "The 340B program had the intention to assist struggling hospitals that help people in need of care, but it's clear we need reform as soon as possible. The ongoing legal battles and massive expansion of the program have created an urgent need to reform 340B through congressional action." | N/A |

© 2023 Knox RP et al. *JAMA Health Forum.*

| Article Citation | Article Type | Study Objective/Article Thesis | Stakeholders Discussed | Results/Analyses | Conclusions/ Recommendations | Limitations |
|---|---|---|---|---|---|---|
| Southwick R. Hospitals cheer court ruling on 340B drug payments: 'An important victory'. Chief Healthcare Executive. Published Sept. 30, 2022. Accessed Apr. 10, 2023. https://www.chiefhealthcareexecutive.com/view/hospitals-cheer-court-ruling-on-340b-drug-payments-an-important-victory- | Commentary | To describe the legal battles over CMS' repayments of 340B reimbursements related to the Supreme Court's decision in *AHA v. Becerra* | Hospitals, federal government | "However, the high court sent the case to the lower federal court for a remedy. The high court case focused on the years 2018 and 2019, but hospitals have fumed because the Medicare cuts have continued through 2022.<br><br>U.S. District Judge Rudolph Contreras of the District of Columbia issued a ruling Wednesday ordering the health department to stop the Medicare cuts for 2022."<br><br>"Hospitals participating in the 340B program have said the reductions in drug payments are hurting their ability to provide care in underserved communities in urban and rural areas. Hospital leaders also said the reduced rates are adding to their already troubled finances. | N/A | N/A |

© 2023 Knox RP et al. *JAMA Health Forum.*

| Article Citation | Article Type | Study Objective/Article Thesis | Stakeholders Discussed | Results/Analyses | Conclusions/ Recommendations | Limitations |
|---|---|---|---|---|---|---|
| Stewardship of the 340B Drug Pricing Program. Baptist Health. Accessed Apr. 10, 2023. https://www.baptist-health.com/stewardship-of-the-340b-drug-pricing-program/ | Hospital Webpage | To describe the hospital's experience with the 340B Program | Hospital, patients | Baptist Health has used 340B Program revenue to:<br>"Free health screenings (blood sugar, blood pressure, body mass index, cholesterol) available to the public that benefit low-income communities and underserved populations.<br>Expanding care that might not otherwise be available to rural communities through rural health clinics.<br>Extended hours at clinics, giving residents an alternative to the emergency room. That, in turn, reduces costs and gives patients one-stop care.<br>Resources and education to help put patients with chronic diseases like diabetes and heart disease on a path to a healthier, more active lifestyle.<br>Virtual healthcare options that offer care online anytime, anywhere.<br>New technology, upgraded equipment and new or renovated facilities." | "Scaling back the 340B Drug Discount Program program could mean a reduction or elimination of the patient services, educational courses, support groups and programs at the participating Baptist Health hospitals mentioned above." | N/A |

© 2023 Knox RP et al. *JAMA Health Forum.*

| Article Citation | Article Type | Study Objective/Article Thesis | Stakeholders Discussed | Results/Analyses | Conclusions/ Recommendations | Limitations |
|---|---|---|---|---|---|---|
| The 340B Drug Discount Program in Review: How Abuse of the 340B Program is Hurting Patients. Community Oncology Alliance. Published Sept. 2017. Accessed Apr. 10, 2023. https://communityoncology.org/wp-content/uploads/2018/06/COA_340B-PatientStories_FINAL.pdf | Report | To describe the harms of abuses of the 340B Program to patients | Hospitals, clinics, patients | 1) Hospitals may not use 340B funds for indigent patients without reporting requirements 2) 340B revenue may support all hospital operations 3) Hospitals may be using 340B for their priorities, not patient priorities 4) Hospitals may be eligible for the 340B Program because one affiliated site is eligible 5) 340B may contribute to oncology care shifting to hospital settings instead of community care settings. | N/A | N/A |

© 2023 Knox RP et al. *JAMA Health Forum.*

| Article Citation | Article Type | Study Objective/Article Thesis | Stakeholders Discussed | Results/Analyses | Conclusions/ Recommendations | Limitations |
|---|---|---|---|---|---|---|
| The 340B Drug Discount Program: A Review and Analysis of the 340B Program. BIO. Published 2013. Accessed Apr. 10, 2023. https://www.bio.org/sites/default/files/legacy/bioorg/docs/340B%20White%20Paper%20FINAL.pdf | Report | "This white paper examines the history and original intent of the program as well as highlights key findings to help policymakers ensure that the 340B program meets its stated purpose and to provide a roadmap for next steps to be considered." | Hospitals, clinics, patients, federal government | "• Adequate funding for the Health Resources and Services Administration (HRSA) is needed to ensure it is appropriately resourced to oversee the 340B program in support of the efforts it has already begun.<br>• Continued oversight of the 340B program is needed to ensure that the program is consistent with its statutory purpose.<br>• Improved transparency is necessary to help advance the program's goals and ensure that resources are being directly used to reduce drug costs for uninsured indigent patients.<br>• Full and transparent accounting for all cost-savings derived from the 340B program should be required to ensure that they are used to reduce drug costs for uninsured indigent patients.<br>• Clearer definition of the term "patient"…<br>• Clarification of hospital eligibility criteria…<br>• 340B guidance should follow formal notice and comment rulemaking…" | "Because of the potentially serious consequences that could evolve from these and other findings, Congress should conduct a thorough examination of the 340B program to ensure it is meeting its original goals." | N/A |

© 2023 Knox RP et al. *JAMA Health Forum.*

| Article Citation | Article Type | Study Objective/Article Thesis | Stakeholders Discussed | Results/Analyses | Conclusions/ Recommendations | Limitations |
|---|---|---|---|---|---|---|
| The 340B Drug Discount Program: Benefits and Challenges for Title X. National Family Planning & Reproductive Health Association. Published Apr. 2013. Accessed Apr. 10, 2023. https://www.nationalfamilyplanning.org/document.doc?id=797 | Report | To describe the benefits and challenges of the 340B Program for Title X clinics | Clinics, patients | "Fluctuations in prices make it difficult for health centers to stock and budget accurately. The frequent price changes also challenge health centers' ability to make timely adjustments to their sliding-fee scales…<br>Participation in the 340B program also comes with several logistical challenges. Title X health centers often use multiple purchasing systems because some methods are only available directly from the manufacturer. Health centers have reported some difficulties organizing the stock needed to meet patient needs because of differing drug purchasing rules. Safeguarding against drug diversion is also an ongoing concern that results in health centers having to maintain separate inventories to monitor which patients access 340B drugs." | "limiting 340B coverage based on insurance status would force covered entities to abandon established relationships with many underinsured and low-income patients in need of affordable drugs. Such a change would be detrimental to the patients and the providers that benefit from the 340B program. NFPRHA and its coalition partners will continue to fight to protect the 340B program, which greatly benefits Title X health centers and their patients." | N/A |

© 2023 Knox RP et al. *JAMA Health Forum.*

| Article Citation | Article Type | Study Objective/Article Thesis | Stakeholders Discussed | Results/Analyses | Conclusions/ Recommendations | Limitations |
|---|---|---|---|---|---|---|
| The 340B Drug Pricing Program and Medicaid Drug Rebate Program: How They Interact. MACPAC. Published May 2018. Accessed Apr. 10, 2023. https://www.macpac.gov/wp-content/uploads/2018/05/340B-Drug-Pricing-Program-and-Medicaid-Drug-Rebate-Program-How-They-Interact.pdf | Report | "This issue brief begins by providing background on the history and mechanics of the Medicaid Drug Rebate Program and 340B. It then describes the issues that state Medicaid programs face in coordinating prescription drug benefits with 340B. It concludes with an overview of two other issues related to 340B: (1) whether covered entities may be using the 340B program to generate revenue, and (2) concerns about whether federal oversight is adequate to monitor the rapidly growing program." | Hospitals, clinics, pharmacies | 1) The complexities of duplicate discounting raise challenges for compliance with 340B and Medicaid.<br>2) "The ability of 340B covered entities to generate revenue from the program without passing along the discount to low-income or uninsured individuals has led some observers to conclude that 340B has moved away from its original mission of serving at-risk populations and has become a funding stream for some providers"<br>3) There are concerns about HRSA's ability to oversee the 340B Program. | N/A | N/A |

© 2023 Knox RP et al. *JAMA Health Forum.*

| Article Citation | Article Type | Study Objective/Article Thesis | Stakeholders Discussed | Results/Analyses | Conclusions/ Recommendations | Limitations |
|---|---|---|---|---|---|---|
| The 340B Drug Pricing Program. Munson Healthcare. Accessed Apr. 10, 2023. https://www.munsonhealthcare.org/about-the-system/advocacy/federal-issues/the-340b-drug-pricing-program | Hospital Webpage | To describe the hospital's experience with the 340B Program | Hospital, patients | "$16.8 million: Amount provided as charity care, in-kind health services, volunteer time, and health education training (FY 16)"<br>"$15.4 million: Cost of uncompensated care for Medicare patients (FY 16)"<br>"These savings also allow us to provide the following community benefits (as of 2016):<br>* Financial Navigators for Cancer Patients: …<br>* Community Health Education: …<br>* Community-Based Clinical Services: …<br>* Healthcare Support Services: ….<br>* Transportation Services: ….<br>* Family Support Services …<br>* Health Professions Education: ….<br>* Community Building Activities: ….<br>* Research….<br>* Charity Care..." | N/A | N/A |

© 2023 Knox RP et al. *JAMA Health Forum.*

| Article Citation | Article Type | Study Objective/Article Thesis | Stakeholders Discussed | Results/Analyses | Conclusions/ Recommendations | Limitations |
|---|---|---|---|---|---|---|
| The 340B Program: How Arbitrage, Opportunism and Opacity Undermine the Program's Original Purpose. Janssen. Published 2022. Accessed Apr. 10, 2023. https://transparencyreport.janssen.com/340b%20report | Commentary | To discuss abuses of the 340B Program | Hospitals, clinics, manufacturers, pharmacies | Hospital, clinic, and contract pharmacy participation in the 340B Program has grown immensely. Manufacturers argue that "Covered entities are currently using the program to reap significant financial windfalls using a complex web of large for-profit pharmacies and PBMs. Arbitrage, opportunism and opacity have supercharged the financial windfall benefiting certain companies and unsustainable expansion of 340B." | "Janssen supports the original intent of the 340B program. Congress created a balanced program designed to allow manufacturers to restore the discounts that they had traditionally provided to safety net providers, before enactment of the Medicaid Drug Rebate Program, while protecting against duplicate discounts and diversion. As the program has grown exponentially in recent years, we believe steps must be taken to ensure that duplicate discounts and diversion are limited. More broadly, we think that reform of the program should require that the benefit of discounts be made directly available to patients at the pharmacy counter. … A lack of transparency in the program limits stakeholders' ability to monitor the program for diversion and duplicate discounts, which are illegal. We believe that this greater transparency will make the program more sustainable." | N/A |

© 2023 Knox RP et al. *JAMA Health Forum.*

| Article Citation | Article Type | Study Objective/Article Thesis | Stakeholders Discussed | Results/Analyses | Conclusions/ Recommendations | Limitations |
|---|---|---|---|---|---|---|
| The Commonwealth Fund. The Federal 340B Drug Pricing Program: What It Is, and Why It's Facing Legal Challenges. Published Sept. 8, 2022. Accessed Apr. 10, 2023. https://www.commonwealthfund.org/publications/explainer/2022/sep/federal-340b-drug-pricing-program-what-it-is-why-its-facing-legal-challenges | Report | To address the controversies regarding the 340B Program | Hospitals, clinics, manufacturers, pharmacies | Manufacturers have critiqued the growth of the 340B Program. Providers have said these critiques are unfounded. Policymakers have sought to protect the 340B Program. | N/A | N/A |

© 2023 Knox RP et al. *JAMA Health Forum.*

| Article Citation | Article Type | Study Objective/Article Thesis | Stakeholders Discussed | Results/Analyses | Conclusions/ Recommendations | Limitations |
|---|---|---|---|---|---|---|
| The Impact of the 340B Program on Drug Prices Charged by Manufacturers and Covered Entities. Health Management Associates. Published Dec. 8, 2022. Accessed Apr. 10, 2023. https://www.healthmanagement.com/blog/the-impact-of-the-340b-program-on-drug-prices-charged-by-manufacturers-and-covered-entities/ | Commentary | To provide insight on the impact of the 340B Program on drug pricing | Manufacturers | "The 340B program is a mandatory program for pharmaceutical manufacturers wishing to participate in the Medicaid drug rebate program. Today, the program has more than 53,000 participating covered entities and the total amount of drugs purchased at the 340B ceiling price under the program is almost $44 billion (Drug Channels). Drug list prices, like other prices in health care, are increasing. The research for this white paper suggests that 340B is one of many factors putting upward pressure on launch prices. Covered entities can generate savings from the prices charged for 340B drugs. There are no requirements for hospitals for how they use the savings, so pricing for services vary. Lack of comprehensive data across the program limits insights on pricing and discount strategies." | N/A | N/A |
| The Value of the 340B Drug Pricing Program to the Community We Serve. Pomona Valley Hospital Medical Center. Published Dec. 27, 2018. Accessed Apr. 10, 2023. https://www.pvhmc.org/blog/2018/december/the-value-of-the-340b-drug-pricing-program-to-th/ | Hospital Webpage | To describe the hospital's experience with the 340B Program | Hospital, patients | The hospital has used 340B savings to provide expanded access to prescription drugs, outpatient chemotherapy treatments, clinical pharmacy services, transportation services, and free vaccinations for vulnerable populations. | N/A | N/A |

© 2023 Knox RP et al. *JAMA Health Forum.*

| **Article Citation** | **Article Type** | **Study Objective/Article Thesis** | **Stakeholders Discussed** | **Results/Analyses** | **Conclusions/ Recommendations** | **Limitations** |
|---|---|---|---|---|---|---|
| Tribble JS, Featherston E. As Big Pharma and Hospitals Battle Over Drug Discounts, Patients Miss Out on Millions in Benefits. Kaiser Health News. Published Nov. 16, 2021. Accessed Apr. 10, 2023. https://kffhealthnews.org/news/article/340b-big-pharma-hospitals-battle-over-drug-discounts-patients-miss-out-on-millions-in-benefits/ | News | To discuss impact of disputes between manufacturers, hospitals, and pharmacies related to the 340B Program on patients | Hospitals, clinics, manufacturers, pharmacies, patients | "U.S. District Court Judge Sarah Evans Barker in Indianapolis wrote that manufacturers believe they are “at the mercy of a system run amok” and that the program “can no longer be held together and implemented fairly” solely through the agency’s guidance and inconsistent messaging."<br>- "CVS reported in an August financial filing that operating income increased by a third between March and June compared with a year ago and noted that 340B business contributed to that increase but provided no further detail. [...]."<br>- "Drugmakers said they continue to participate in the program by sending direct discounts to the hospitals but have eliminated some or all of the discounts passed through contract pharmacies because they didn’t trust the transactions, according to emails the companies sent to KHN and InvestigateTV. Novartis, which announced last year that it would sell drugs at a discount only for pharmacies within 40 miles of a hospital, said there is a “complete absence of transparency” in the contracts between hospitals and pharmacies." | N/A | N/A |

© 2023 Knox RP et al. *JAMA Health Forum.*

| Article Citation | Article Type | Study Objective/Article Thesis | Stakeholders Discussed | Results/Analyses | Conclusions/ Recommendations | Limitations |
|---|---|---|---|---|---|---|
| Twenter P. 21st drugmaker curbs 340B drug discounts, nonprofit says. Becker's Hospital Review. Published Feb. 1, 2023. Accessed Apr. 10, 2023. https://www.beckershospitalreview.com/pharmacy/20th-drugmaker-curbs-340b-drug-discounts-nonprofit-says.html | News | To update readers on manufacturers' responses to the contract pharmacy litigation | Hospitals, clinics, manufacturers, pharmacies | "340B Health said these are the 20th and 21st drugmakers to narrow discounted drugs to 340B hospitals, which care for an uninsured community. The others are AbbVie, Amgen, AstraZeneca, Bausch Health, Biogen, Boehringer Ingelheim, Bristol Myers Squibb, Eli Lilly, Exelixis, Gilead, GlaxoSmithKline, Johnson & Johnson, Merck, Novartis, Novo Nordisk, Pfizer, Sanofi, UCB and United Therapeutics, the organization said." | N/A | N/A |

© 2023 Knox RP et al. *JAMA Health Forum.*

| Article Citation | Article Type | Study Objective/Article Thesis | Stakeholders Discussed | Results/Analyses | Conclusions/ Recommendations | Limitations |
|---|---|---|---|---|---|---|
| Understanding the 340B Program. CHOC. Accessed Apr. 10, 2023. https://www.choc.org/about/340b-program/ | Hospital Webpage | To describe the hospital's experience with the 340B Program | Hospital, patients | "The 340B program supports our goal of meeting the needs of the community by increasing access to quality pediatric healthcare resources to Orange County families, especially low-income and medically-underserved children. Savings generated from the 340B program enables us to provide vital services to the community at low or no cost, including but not limited to:<br><br>Behavioral health services, including 18 inpatient mental health beds<br>Bedside discharge prescription services to reduce preventable hospital readmission rates<br>Clinical pharmacy services to patients with serious medical conditions to improve medication adherence and outcomes<br>Subsidies for chemotherapy and other specialty medications for Medicaid and uninsured patients requiring continuation of care<br>Promising new treatments, such as gene therapies and CAR-T cell treatments<br>New programs, including the Thompson Autism Center" | N/A | N/A |

© 2023 Knox RP et al. *JAMA Health Forum.*

| Article Citation | Article Type | Study Objective/Article Thesis | Stakeholders Discussed | Results/Analyses | Conclusions/ Recommendations | Limitations |
|---|---|---|---|---|---|---|
| US Health Resources Services Administration.340B Drug Pricing Program; Administrative Dispute Resolution. Fed Regist. 2022;87(229):73516-73527. | Federal Register Notice | To propose reforms to the administrative dispute resolution process in the 340B Program | Hospitals, clinics, manufacturers | This notice sets forth proposed reforms to the ADR process | N/A | N/A |

© 2023 Knox RP et al. *JAMA Health Forum.*

| Article Citation | Article Type | Study Objective/Article Thesis | Stakeholders Discussed | Results/Analyses | Conclusions/ Recommendations | Limitations |
|---|---|---|---|---|---|---|
| USC Arcadia Hospital 340B Good Stewardship Principles. USC Arcadia Hospital. Accessed Apr. 10, 2023. https://www.uscarcadiahospital.org/For-Patients-Visitors-Vendors/340B-Program.aspx | Hospital Webpage | To describe the hospital's experience with the 340B Program | Hospital, patients | "The 340B Program enables patients to access needed prescription medications regardless of income level or insurance coverage and enables Methodist Hospital to provide a variety of needed treatments and services, including:<br><br>Medications upon discharge at no cost to patients who might otherwise find such medication unaffordable<br>Access to financial assistance with the purchase of specialty medications<br>Clinical pharmacy services to patients with serious medical conditions to improve medication adherence and outcomes<br>Access to specialty services and clinical expertise<br>Free health screenings in local settings accessible to homeless and low-income populations<br>Opioid stewardship program to help affected patients and families<br>No-cost nicotine replacement therapy for patients trying to quit smoking" | "In 2018, USC Arcadia Hospital provided charity care and in-kind health services valued at $18 million. The hospital's cost of uncompensated care provided to Medi-Cal (Medicaid) and Medicare patients was $27 million during 2018. Key medicines provided through the 340B program at Methodist Hospital include contrast media, tPA, Lexiscan, Isuprel, Vasopressin, and Propofol. Scaling back the 340B Program would put at risk Methodist Hospital's ability to provide many of the services and medications described above to uninsured and under-insured patients." | N/A |

© 2023 Knox RP et al. *JAMA Health Forum.*

| **Article Citation** | **Article Type** | **Study Objective/Article Thesis** | **Stakeholders Discussed** | **Results/Analyses** | **Conclusions/ Recommendations** | **Limitations** |
|---|---|---|---|---|---|---|
| Value of the 340B Program. Saint Agnes Medical Center. Published 2018. Accessed Apr. 10, 2023. https://www.samc.com/assets/documents/fresno-saint-agnes-medical-center-narrative.pdf | Hospital Webpage | To describe the hospital's experience with the 340B Program | Hospital, patients | The 340B Program allows the hospital to maintain vital healthcare services and support and nourish community health programs.<br><br>"Saint Agnes Medical Center qualifies for the 340B program, which provides significant care to the vulnerable population, including $27,055,279 in total community benefit during the last fiscal year." | "IMPACT IF THE PROGRAM WAS SCALED BACK:<br>* Reduction of Provider Network and Urgent Care Centers: These two essential pieces of our organization form the basis for comprehensive and cost-effective care for our patient population. Reduction in urgent care access will result in increased and more costly visits to the Emergency Department.<br>* Potential reduction to profession education support in an area that is already medically underserved<br>* Negative impact on patient care due to lessened resources to reinvest in facilities, equipment, workforce and other strategic initiatives." | N/A |

© 2023 Knox RP et al. *JAMA Health Forum.*

| Article Citation | Article Type | Study Objective/Article Thesis | Stakeholders Discussed | Results/Analyses | Conclusions/ Recommendations | Limitations |
|---|---|---|---|---|---|---|
| Value of the 340B Program. St. Peter's Health Partners. Accessed Apr. 10, 2023. https://www.sphp.com/assets/documents/communitybenefit/sphp-comm-benefits-340b-plan_flyer.pdf | Hospital Webpage | To describe the hospital's experience with the 340B Program | Hospital, patients | "In fiscal year 2020, SPHP provided more than $60 million in total community benefit. These efforts included preventive services; community partnerships; the preparation of future health care professionals; uncompensated care, research and clinical trials; programs that impact well-being and health status; and efforts to meet the health needs of the uninsured, low-income and other vulnerable populations." * "Along with our annual $19.7 million in uncompensated care … Samaritan Hospital uses its 340B savings to help care for all patients, regardless of their ability to pay. This includes free care for uninsured patients for services including cancer care and infusion services; free vaccines; mental health services; community health programs including prostate and cardiac and vascular screenings; and medication management and prescription assistance. Through our prescription assistance program we were able to assist 1,846 individuals in fiscal year 2020 obtain needed medications…" | N/A | N/A |

© 2023 Knox RP et al. *JAMA Health Forum.*

| Article Citation | Article Type | Study Objective/Article Thesis | Stakeholders Discussed | Results/Analyses | Conclusions/ Recommendations | Limitations |
|---|---|---|---|---|---|---|
| What Is The 340B Drug Pricing Program?. Azalea Health. Accessed Apr. 10, 2023. https://www.azaleahealth.com/blog/340b/ | Hospital Webpage | To describe health system experiences with 340B | Hospital, patients | "Without these funds, CAHs, Community Hospitals, and their associated outpatient facilities would need to either find new revenue streams or new federal funding (potentially from taxpayers), turn away uninsured or under-insured patients, cut patient care programs or worst-case scenario, close and discontinue patient services." | N/A | N/A |

© 2023 Knox RP et al. *JAMA Health Forum.*

| Article Citation | Article Type | Study Objective/Article Thesis | Stakeholders Discussed | Results/Analyses | Conclusions/ Recommendations | Limitations |
|---|---|---|---|---|---|---|
| What is the 340B Program?. University of Utah Health. Accessed Apr. 10, 2023. https://healthcare.utah.edu/about/340b-program | Hospital Webpage | To describe the hospital's experience with the 340B Program | Hospital, patients | The 340B Program is used for:<br>1. Uncompensated and charity care provided.<br>2. "Providing patients access to medications through our Medication Support Services (MSS) program. Patients who qualify for the MSS program receive their medications free of charge from U of U Health pharmacies."<br>3. "Subsidizing health care services that operate at a loss, including mental health, substance abuse, psychiatry, crisis intervention, primary care, HIV, air ambulance, and other outpatient specialty services."<br>4. "Providing free or discounted clinical services to the public, including skin cancer screenings, physical exams, diabetes screenings, and mental health assessments."<br>5. Expanding telehealth and translation services. | N/A | N/A |

© 2023 Knox RP et al. *JAMA Health Forum.*

| Article Citation | Article Type | Study Objective/Article Thesis | Stakeholders Discussed | Results/Analyses | Conclusions/ Recommendations | Limitations |
|---|---|---|---|---|---|---|
| What You Need to Know About the 340B Drug Pricing Program. Chronic Care Policy Alliance. Published July 12, 2021. Accessed Apr. 10, 2023. https://chroniccarealliance.org/what-you-need-to-know-about-the-340b-drug-pricing-program/ | Report | To evaluate changes the 340B Program | Hospitals, clinics, patients | "340B Saw 400% Growth in Less Than a Decade. Between 2012 and 2020, the 340B program grew over 400%, with total 340B sales at discounted prices approaching $38 billion in 2020 alone. Unfortunately, the growth in 340B sales did not translate into better treatment access for patients. Bad actors are taking advantage of a program that allows qualifying entities to buy Rx drugs and treatments at a discounted rate with no obligation to pass on the savings to patients. …. Qualifying hospitals have been acquiring independent community healthcare practices at an alarming rate to consolidate care into costlier hospital settings. In turn, this leaves uninsured and indigent patient populations who rely on 340B to access their medicines with fewer care options.<br>A small number of 340B hospitals provide the vast majority of care to patients. Just 24% of all 340B hospitals provide 80% of the total charity care provided by hospitals in the program. This means deserving patients have fewer care options, and the care options they do have are overburdened." | "For vulnerable, 340B eligible patients with cancer, HIV, or any other complex disease, the question must be asked: is 340B still providing affordable access to necessary treatments and medications as it was intended to, or is it only serving the hospitals that make up the program?<br><br>As lawmakers look for ways to improve healthcare equity and prescription drug access, they should bring greater oversight and transparency to 340B and modernize the program to better serve patients." | N/A |

| Article Citation | Article Type | Study Objective/Article Thesis | Stakeholders Discussed | Results/Analyses | Conclusions/ Recommendations | Limitations |
|---|---|---|---|---|---|---|
| Whitner J. What the 340B Program Means to an Ohio Health Center's Patients. Association of Clinicians for the Underserved. Published Aug. 9, 2022. Accessed Apr. 10, 2023. https://clinicians.org/340b-what-it-means-to-health-center-patients/ | Hospital Webpage | To describe the hospital's experience with the 340B Program | Hospital, patients | "P1H provides discounted medication pricing to all uninsured patients with incomes at 200% or less of the Federal Poverty Level. In 2021 we saved our patients millions on prescription medications through the 340B program. … PrimaryOne Health uses the remaining 340B savings to expand access to the following services that we would otherwise not be able to provide or only in limited capacity: substance use disorder and addiction services, language assistance / interpreter services, physical therapy, dental care, clinical pharmacy, vaccines, and nutrition." | N/A | N/A |

© 2023 Knox RP et al. *JAMA Health Forum.*

| Article Citation | Article Type | Study Objective/Article Thesis | Stakeholders Discussed | Results/Analyses | Conclusions/ Recommendations | Limitations |
|---|---|---|---|---|---|---|
| Why the 340B Drug Discount Program Must Survive. Greene County Health Care. Published Sept. 15, 2020. Accessed Apr. 10, 2023. https://gchcinc.org/why-the-340b-drug-discount-program-must-survive/ | Hospital Webpage | To describe the hospital's experience with the 340B Program | Hospital, patients | * "For Community Health Centers, 340B discounts make medications and services affordable for their 30 million patients."<br>* "With contract pharmacies, our population can now access low-cost medicines they need in their home county. We make 340B available to all uninsured patients and to all patients who get their medications at our contract pharmacies. Without 340B, we would not be able to serve our patients in this manner."<br>*"Greene County Health Care uses these savings to support programs that increase access to care in our centers, including medication assistants who access free medications from the indigent programs of pharmaceutical companies and getting other free medications and donations including PPE. The savings also support other programs that increase access to care here in Eastern North Carolina." | N/A | N/A |

© 2023 Knox RP et al. *JAMA Health Forum.*

| Article Citation | Article Type | Study Objective/Article Thesis | Stakeholders Discussed | Results/Analyses | Conclusions/ Recommendations | Limitations |
|---|---|---|---|---|---|---|
| Winegarden W. Addressing the Problems of Abuse in the 340B Drug Pricing Program. Pacific Research Institute. Published Dec. 2017. Accessed Apr. 10, 2023. https://www.pacificresearch.org/wp-content/uploads/2017/12/340B-Study_FINAL.pdf | Report | To describe problems related to abuse of the 340B Program and possible solutions | Hospitals, clinics, manufacturers, pharmacies | 1) The ACA resulted in increases in participation and sales in the 340B Program.<br>2) Recent expansions of the 340B Program are not reaching targeted populations.<br>3) The 340B Program is encouraging the use of higher-priced medicines and incentivizing provider consolidation.<br>340B Encourages Covered Entities to Prescribe Higher Priced Medicines | "The best politically feasible reform would return the scope of the 340B program to its original purpose of serving uninsured and low-income patients, and improve the oversight and administration of the program. Specifically, Congress and the Administration should update the law governing the 340B program so that it clearly defines who qualifies for the discount; which should only include those patients who are truly in need. …<br>Additionally, since the program is intended to help the vulnerable populations, measures should be adopted that ensure that the 340B savings are passed along to the uninsured patients when filling their prescriptions at the covered entities or their contract pharmacies. …<br>More stringent oversight practices should also be implemented to eliminate the proliferation of abuse in the program." | N/A |

© 2023 Knox RP et al. *JAMA Health Forum.*

| Article Citation | Article Type | Study Objective/Article Thesis | Stakeholders Discussed | Results/Analyses | Conclusions/ Recommendations | Limitations |
|---|---|---|---|---|---|---|
| Yood K, Kraus E, Thompson T. Contract Pharmacies and the 340B Drug Discount Program: New Litigation and an Advisory Opinion Point to Ongoing Skirmishes on the 340B Battlefield. Sheppard Mullin. Published Jan. 8, 2021. Accessed Apr. 10, 2023. https://www.sheppardhealthlaw.com/2021/01/articles/drug-and-pharmaceutical-law/contract-pharmacies-340b-program-litigation/ | Report | To review the ongoing litigation related to 340B contract pharmacies | Hospitals, clinics, manufacturers, pharmacies | "First, advisory opinions are not final agency action or final order, nor do they have the force or effect of law. Therefore, whereas a court judgement would be binding upon the litigants, Ad. Op. 20-06 is not. Second, Ad. Op. 20-06 does not address a key aspect of the litigation – enforcement against manufacturers who fail to abide by HRSA's past guidance and the conclusions reached in Ad. Op. 20-06. So while HHS through Ad. Op. 20-06 advises manufacturers that they may not refuse to offer the ceiling price to covered entities, even when the covered entity at issue uses distribution systems involving contract pharmacies, Ad. Op. 20-06 is silent as to the enforcement mechanism that HHS will use to compel compliance." | "AHA noted that they now expect HRSA to, "take swift and decisive action to halt these pernicious tactics from drug companies and ensure that 340B drugs remain available and accessible to vulnerable communities across the country." AHA also called for HRSA to, "ensure that hospitals are made whole as a result of being denied appropriate discounts since these illegal practices began earlier this year."<br>Based upon the above and the positions taken by the other Plaintiffs, if HRSA does not proceed with action beyond the release of Ad. Op. 20-06, it seems unlikely that the 340B Program Litigation will be dropped anytime soon." | N/A |

© 2023 Knox RP et al. *JAMA Health Forum.*

| Article Citation | Article Type | Study Objective/Article Thesis | Stakeholders Discussed | Results/Analyses | Conclusions/ Recommendations | Limitations |
|---|---|---|---|---|---|---|
| Yood K. A Year of Living Dangerously: 2020 and Maneuvers on the 340B Drug Pricing Program Battlefield. National Law Review. Published Sept. 30, 2020. Accessed Apr. 10, 2023. https://www.natlawreview.com/article/year-living-dangerously-2020-and-maneuvers-340b-drug-pricing-program-battlefield | Commentary | To discuss ongoing issues with the 340B Program | Hospitals, clinics, manufacturers | "if HHS refuses to take action against the drugmakers, the strident rhetoric of the AHA and other 340B Program stakeholders points to a willingness by such organizations to take matters into their own hands by taking drugmakers to court and lobbying Congress to give HRSA more enforcement authority over the 340B Program and, specifically, the ability to enforce HRSA's contract pharmacy guidance….<br><br>Congress, who share our view that this behavior is not only unlawful but also harmful to patients in need and to the dedicated health professionals who care for them. We reiterate our call on Secretary Azar to act now." | N/A | N/A |

© 2023 Knox RP et al. *JAMA Health Forum.*
