# Supplementary material for: Outcomes of the 340B Drug Pricing Program: A Scoping Review
Source: JAMA Health Forum. 2023 Nov 22;4(11):e233716. doi: 10.1001/jamahealthforum.2023.3716 (PMC10665972; doi:10.1001/jamahealthforum.2023.3716)
Supplement: Supplement 2. — Data Sharing Statement [file jamahealthforum-e233716-s002.pdf]

Data Sharing Statement

Data
Data available: Yes
Data types: Data (not involving human participants)
How to access data: Data will be presented in the Appendices.
When available: With publication

Supporting Documents
Document types: None

Additional Information
Who can access the data: The data will be available to everyone
Types of analyses: For any purpose
Mechanisms of data availability: Without investigator support
Any additional restrictions: None
